# A *Jacob/Nsmf* Gene Knockout Results in Hippocampal Dysplasia and Impaired BDNF Signaling in Dendritogenesis

**DOI:** 10.1371/journal.pgen.1005907

**Published:** 2016-03-15

**Authors:** Christina Spilker, Sven Nullmeier, Katarzyna M. Grochowska, Anne Schumacher, Ioana Butnaru, Tamar Macharadze, Guilherme M. Gomes, PingAn Yuanxiang, Gonca Bayraktar, Carolin Rodenstein, Carolin Geiseler, Angela Kolodziej, Jeffrey Lopez-Rojas, Dirk Montag, Frank Angenstein, Julia Bär, Wolfgang D’Hanis, Thomas Roskoden, Marina Mikhaylova, Eike Budinger, Frank W. Ohl, Oliver Stork, Ana C. Zenclussen, Anna Karpova, Herbert Schwegler, Michael R. Kreutz

**Affiliations:** 1 Research Group Neuroplasticity, Leibniz Institute for Neurobiology, Magdeburg, Germany; 2 Institute of Anatomy, Medical Faculty, Otto-von-Guericke University, Magdeburg, Germany; 3 Department of Experimental Obstetrics and Gynaecology, Medical Faculty, Otto-von-Guericke University, Magdeburg, Germany; 4 Department of Systems Physiology of Learning, Leibniz Institute for Neurobiology, Magdeburg, Germany; 5 Special Laboratory Neurogenetics, Leibniz Institute for Neurobiology, Magdeburg, Germany; 6 Functional Neuroimaging Group, Deutsches Zentrum für Neurodegenerative Erkrankungen (DZNE), and Special Laboratory for Noninvasive Brain Imaging, Leibniz Institute for Neurobiology, Magdeburg, Germany; 7 University Medical Center Hamburg-Eppendorf, Center for Molecular Neurobiology, ZMNH, Emmy-Noether Group 'Neuronal Protein Transport', Hamburg, Germany; 8 Institute of Biology, Otto von Guericke University, Magdeburg, Germany; 9 University Medical Center Hamburg-Eppendorf, Center for Molecular Neurobiology, ZMNH, Leibniz Group 'Dendritic Organelles and Synaptic Function', Hamburg, Germany; Katholieke Universiteit Leuven, BELGIUM

## Abstract

Jacob, the protein encoded by the *Nsmf* gene, is involved in synapto-nuclear signaling and docks an N-Methyl-D-Aspartate receptor (NMDAR)-derived signalosome to nuclear target sites like the transcription factor cAMP-response-element-binding protein (CREB). Several reports indicate that mutations in *NSMF* are related to Kallmann syndrome (KS), a neurodevelopmental disorder characterized by idiopathic hypogonadotropic hypogonadism (IHH) associated with anosmia or hyposmia. It has also been reported that a protein knockdown results in migration deficits of Gonadotropin-releasing hormone (GnRH) positive neurons from the olfactory bulb to the hypothalamus during early neuronal development. Here we show that mice that are constitutively deficient for the *Nsmf* gene do not present phenotypic characteristics related to KS. Instead, these mice exhibit hippocampal dysplasia with a reduced number of synapses and simplification of dendrites, reduced hippocampal long-term potentiation (LTP) at CA1 synapses and deficits in hippocampus-dependent learning. Brain-derived neurotrophic factor (BDNF) activation of CREB-activated gene expression plays a documented role in hippocampal CA1 synapse and dendrite formation. We found that BDNF induces the nuclear translocation of Jacob in an NMDAR-dependent manner in early development, which results in increased phosphorylation of CREB and enhanced CREB-dependent *Bdnf* gene transcription. *Nsmf* knockout (ko) mice show reduced hippocampal *Bdnf* mRNA and protein levels as well as reduced pCREB levels during dendritogenesis. Moreover, BDNF application can rescue the morphological deficits in hippocampal pyramidal neurons devoid of Jacob. Taken together, the data suggest that the absence of Jacob in early development interrupts a positive feedback loop between BDNF signaling, subsequent nuclear import of Jacob, activation of CREB and enhanced *Bdnf* gene transcription, ultimately leading to hippocampal dysplasia.

## Author Summary

Kallmann syndrome (KS) is a neurodevelopmental disorder that is characterized by a migration deficit of GnRH neurons that fail to migrate into the hypothalamus during embryonic development. The affected patients suffer from idiopathic hypogonadotropic hypogonadism associated with anosmia or hyposmia. Mutations in NSMF (NMDA Receptor Synaptonuclear Signaling And Neuronal Migration Factor) have been associated with KS. In previous work we could show that Jacob, the protein encoded by the *Nsmf* gene, is involved in NMDA receptor synaptonuclear signaling. Jacob operates as a mobile hub that docks NMDA receptor-derived signalosomes to nuclear target sites and thereby plays a role in activity-dependent gene transcription. We found that mice that are deficient for the *Nsmf* gene do not present phenotypic characteristics related to KS. Instead, these mice exhibit hippocampal dysplasia with a reduced number of synapses and simplification of dendrites, reduced plasticity at CA1 synapses and deficits in hippocampus dependent learning. Brain-derived neurotrophic factor (BDNF) induces the nuclear translocation of Jacob in an NMDAR-dependent manner in early development, which results in increased phosphorylation of CREB and enhanced CREB-dependent *Bdnf* gene transcription. *Nsmf* knockout mice show reduced hippocampal *Bdnf* mRNA and protein levels as well as reduced pCREB levels during dendritogenesis. Moreover, BDNF application can rescue the morphological deficits in hippocampal pyramidal neurons devoid of Jacob. Taken together, the data suggest that the absence of Jacob in early development interrupts a positive feedback loop between BDNF signaling, subsequent nuclear import of Jacob, activation of CREB and enhanced *Bdnf* gene transcription, ultimately leading to hippocampal dysplasia.

## Introduction

Jacob is a protein that shuttles to the nucleus in response to activation of synaptic and extrasynaptic GluN2B-containing N-methyl-D-aspartate receptors (NMDARs) [1] and that following long-distance transport and nuclear import, encodes and transduces the synaptic and extrasynaptic origin of NMDAR signals to the nucleus [2]. Extracellular-signal-regulated kinase (ERK1/2)-binding and ERK-dependent phosphorylation of a crucial serine at position 180 in Jacob encodes synaptic but not extrasynaptic NMDAR activation. A stable trimeric complex with proteolytically cleaved fragments of the neurofilament α-internexin is formed which protects Jacob and active ERK against phosphatase activity during retrograde transport. In the nucleus this signalosome-like complex enhances 'plasticity-related' and 'CREB-dependent' gene expression as well as synaptic strength [2]. In stark contrast, following extrasynaptic NMDAR activation nuclear import of Jacob results in sustained dephosphorylation and transcriptional inactivation of the transcription factor CREB (CREB shut-off), loss of synaptic contacts, retraction of dendrites and eventually cell death [1,2]. In addition Jacob couples pathological Amyloid-β signaling to the nucleus via extrasynaptic GluN2B-NMDAR activation [3,4] and several lines of evidence suggest that the protein is involved in both neuronal plasticity as well as neurodegeneration.

However, some reports indicate that the mouse orthologue of Jacob called nasal embryonic luteinizing hormone-releasing hormone (LHRH) factor (NELF) is essential for the migration of GnRH-expressing cells from the olfactory bulb to the hypothalamus during neuronal development [5,6]. These studies suggest that the protein functions as an extracellular guidance molecule on growth cones, which is essential for axon growth and routing of GnRH positive cells along vomeronasal olfactory-derived axons and eventually migration to the hypothalamus [5,6]. Several subsequent publications have tried to establish a link between mutations in the *NSMF* gene that encodes for Jacob/NELF and Kallmann syndrome (KS) [7–14]. One of these reports even suggests a monogenic causation of KS by a point mutation in the *NSMF* gene [13]. KS is a rare neurodevelopmental disorder that is characterized by a migration deficit of GnRH neurons that fail to migrate into the hypothalamus during embryonic development [15]. Mutations in several genes have been postulated to cause hypogonadism, delayed or absent puberty and almost invariably infertility [15]. Besides hypogonadotropic hypogonadism, KS patients exhibit anosmia or severe hyposmia and variable other phenotypes that depend upon the type of gene mutation that might underlie the condition [14].

Unfortunately, a number of discrepancies between reports on Jacob and NELF (see above and [16]) exist that obscure the interpretation of previous results. In brief, apart from reported differences in the subcellular localization and function also the originally published open reading frame of *Nelf* did not match to those of *Jacob* [5,1]. Most studies on loss of function phenotypes in either development or adulthood have been performed with *in vitro* preparations and utilized antisense oligonucleotides [5,6] or shRNA protein knockdown of Jacob/NELF [1,2]. Thus, deletion of the gene *in vivo* could potentially clarify some of the controversial issues. We therefore first sought to determine whether inactivation of the *Nsmf* gene results in phenotypes related to KS. Second, since very little is known about the role of Jacob in hippocampal development we examined whether hippocampal circuitry and function is affected by the lack the activity-dependent nuclear import of the protein in *Jacob/Nsmf* ko mice.

## Results

### *Jacob/Nsmf* ko mice show normal fertility and no indication of severe hypogonadotropic hypogonadism

In order to delete Jacob in mice a targeting construct was generated by flanking the first three exons of the *Nsmf* gene with loxP sites to allow for Cre-mediated deletion in all tissues (S1A Fig). Knockout was verified by genomic PCR (S1B Fig), RT-PCR (S1C Fig) and by western blotting (S1D Fig). Western Blots showed that the Jacob protein is expressed at high levels in mouse hippocampus and cortex, but is also present in other brain regions such as the striatum, hypothalamus and olfactory bulb. In heterozygous animals, protein expression is already markedly reduced and in ko mice the Jacob protein is not detectable (S1D Fig).

*Jacob/Nsmf* ko mice are viable and fertile, homozygous (-/-), heterozygous (+/-) and wild-type (wt, +/+) breeding pairs show comparable litter size and a Mendelian ratio as expected (S1E Fig). Body weights were registered upon weaning (20–22 days) and at the age of 4 months. No differences could be observed among the genotypes (S1E Fig).

**Fig 1:**
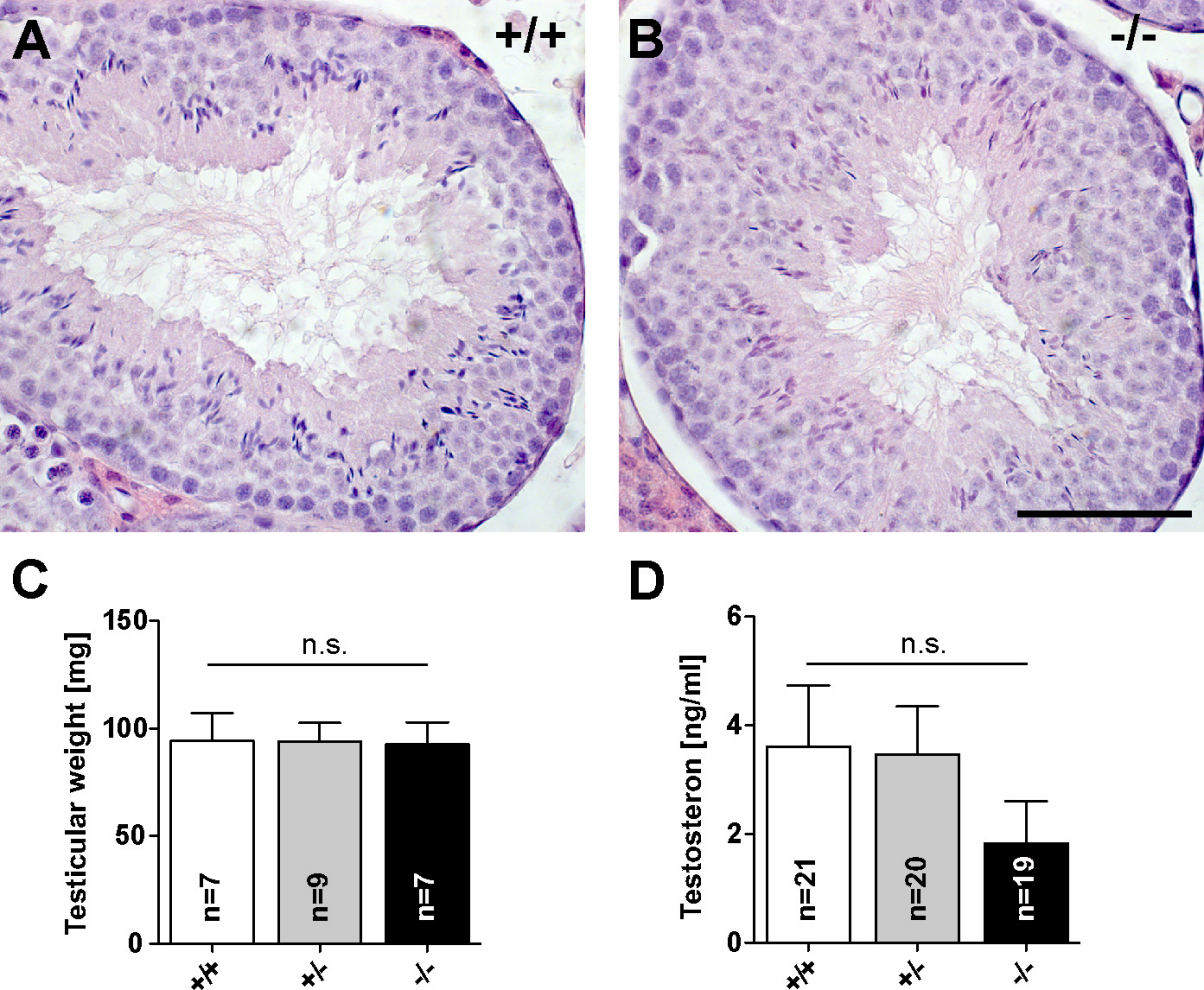
Fertility parameters of male *Jacob/Nsmf* ko mice. (A, B) Testicular morphology in adult wt (A, +/+) and *Jacob/Nsmf* ko (B, -/-) mice testes stained by hematoxylin and eosin protocol. WT and ko mice showed no differences in appearance of different stages of differentiation from spermatogonia to sperm cells and comparable lumina. (C) Testicular weight of wt (+/+), heterozygous (+/-) and *Jacob/Nsmf* ko (-/-) mice. (D) Testosteron serum levels were determined in mice of all genotypes by ELISA. Unpaired t-test; *p<0.05. Scale bar in B is 100μm.

We next investigated whether a gonadal defect evoked by the lack of Jacob expression is present in male and female mice. We found that the morphology and weight of *Jacob/Nsmf* ko and wt testes were unaltered. Hematoxylin and eosin staining revealed a comparable testicular anatomy (Fig 1A and 1B). *Jacob/Nsmf* ko and wt mice showed no differences in appearance of various stages of differentiation from spermatogonia to sperm cells. All stages of spermatogenesis were observed within the tubules. Furthermore, no differences between genotypes were observed in chromosome preparations (S2 Fig). Individual chromosomes can be distinguished during mitosis of spermatogonia (S2A and S2E Fig). In diakinesis-metaphase I, the tetrads of the homologous chromosomes can be seen (S2B and S2F Fig). In metaphase II the separation of the chromatids of each chromosome are visible (S2C, S2D, S2G and S2H Fig). Additionally, testes of both genotypes were similar in mean size and weight (Fig 1C). To exclude impaired infertility due to hormonal abnormalities, we additionally measured testosterone serum levels in all three genotypes. In male ko mice testosterone serum levels were slightly but not statistically significant reduced (Fig 1D).

**Fig 2:**
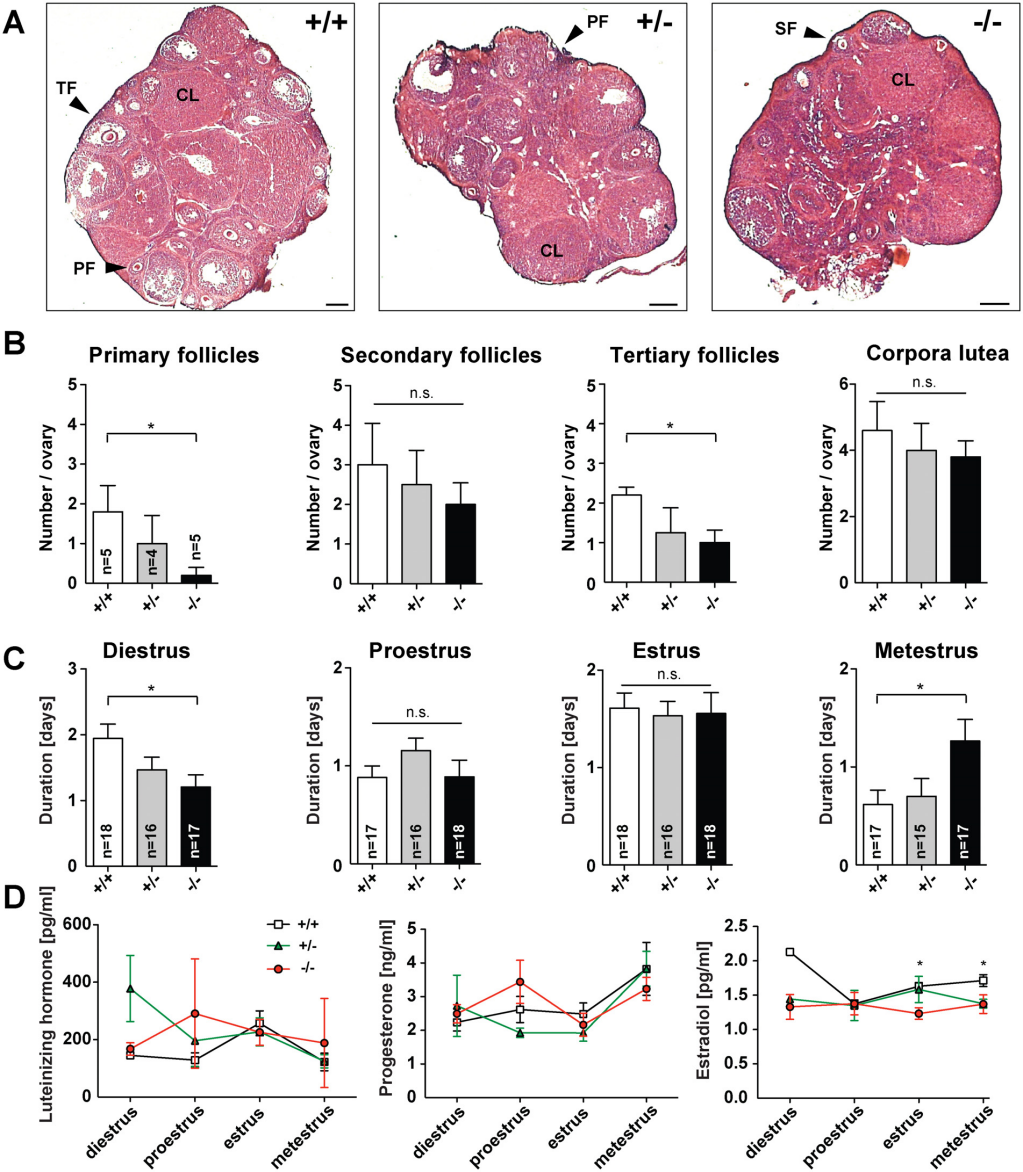
Fertility parameters of female *Jacob/Nsmf* ko mice. (A) Representative pictures of ovarian tissues from wt, heterozygous and homozygous *Jacob/Nsmf* females (PF, primary follicle; SF, secondary and TF, tertiary follicle; CL, corpora lutea). (B) Morphological analysis revealed significant lower numbers of primary and tertiary follicles in ovaries from homozygous females when compared to wt females. No statistical differences could be observed among all groups in the number of secondary follicles and corpora lutea (unpaired t-test; *p<0.05). (C) The average duration (days) of each of the four estrous cycle stages in wt, heterozygous and *Jacob/Nsmf* ko females was determined. Significant differences could be detected in the duration of diestrus and metestrus of *Jacob/Nsmf* ko females when compared to wt females (unpaired t-test; *p<0.05). (D) Hormonal changes throughout the estrous cycle of wt, heterozygous and homozygous *Jacob/Nsmf* females. Mice were sacrificed in different estrous cycle phases; diestrus (n = 3), proestrus (n = 3), estrus (n = 8) and metestrus (n = 3). Graphs display hormonal plasma level of Luteinizing hormone (LH), Progesteron (P4) and Estrogen (E2) in all cycle phases. Hormone levels were compared between all three genotypes. Significant differences in plasma E2 levels could be found between Jacob-deficient and wt females in estrus and metestrus, while no alterations in LH and P4 plasma levels could be observed among all groups throughout the estrous cycle. Unpaired t-test; *p<0.05. Scale bar in A is 200 μm.

In the next set of experiments, *Jacob/Nsmf* heterozygous and homozygous females were examined in comparison to wild-type (wt) littermates regarding reproductive organs, estrous cycle phases and sex hormones. Analysis of each estrous cycle phase revealed that Jacob deficiency prolonged the duration of the metestrus phase (Fig 2C) while shortening the diestrus phase. Statistically significant differences were found in both phases between *Jacob/Nsmf* ko and wt female mice. By contrast, no differences could be observed in the duration of the proestrus and estrus phase among all groups (Fig 2C). An adequate folliculogenesis is a prerequisite for ovulation. To study the influence of Jacob deficiency on follicle development and ovulation we determined the number of the different follicle stages and corpora lutea. Fig 2A shows representative pictures of ovarian tissue from all genotypes. We found statistically significant lower numbers of primary and tertiary follicles in ko females when compared to wt females (Fig 2B). In addition, the number of secondary follicles but not the number of corpora lutea was slightly reduced in Jacob deficient females compared to wt animals (Fig 2B).

Hormonal fluctuations during the estrous cycle are indispensable to ensure proper folliculogenesis, ovulation and preparation of the endometrium for implantation. Since the lack of Jacob affects the number of primary and tertiary follicles we sought to study whether these effects are associated with alterations in hormonal levels. Plasma levels of Luteinizing hormone and Progesterone were comparable among all genotypes throughout the estrous cycle (Fig 2D), while Estradiol levels were significantly reduced in the estrus and metestrus phase when Jacob was absent (Fig 2D). Taken together the data suggest that a *Jacob/Nsmf* gene ko has a modest effect on reproductive parameters in female mice but does not lead to hypogonadotropic hypogonadism or subfertility (see S1E Fig).

### Jacob-deficient mice do not show signs of anosmia or hyposmia and have a normal organization of the bulbus olfactorius

Since anosmia or hyposmia is observed in about 60% of patients with IHH and a distinctive feature for KS [17] we next tested whether Jacob-deficient mice display deficits in olfactory behavior. Synthetic 2,5-Dihydro-2,4,5 trimethylthiazoline (TMT), a predator cue isolated from red fox anal secretion, is frequently used to induce unconditioned fear in rodents. Diethylphthalate (DEP) was used as a solvent for dilution of TMT and served as blank control in odor exposure experiments. *Jacob/Nsmf* ko and wt mice were individually exposed for 15 min to TMT or DEP (S3 Fig and S1 Table). The exposition to TMT or DEP induced similar behavioral effects in mice from both genotypes (S1 Table). Freezing levels were significantly higher in mice exposed to TMT, indicating that the predator odor was smelt (S3 Fig). In contrast, mice treated with DEP showed significantly higher levels in sniffing, grooming and scratching, more rearing in the center area and leaning on the wall and higher locomotor activity (S1 Table). However, no differences between genotypes were found in any of the other analyzed parameters except that *Jacob/Nsmf* ko mice produced a higher number of fecal boli during the odor exposition (S1 Table).

**Fig 3:**
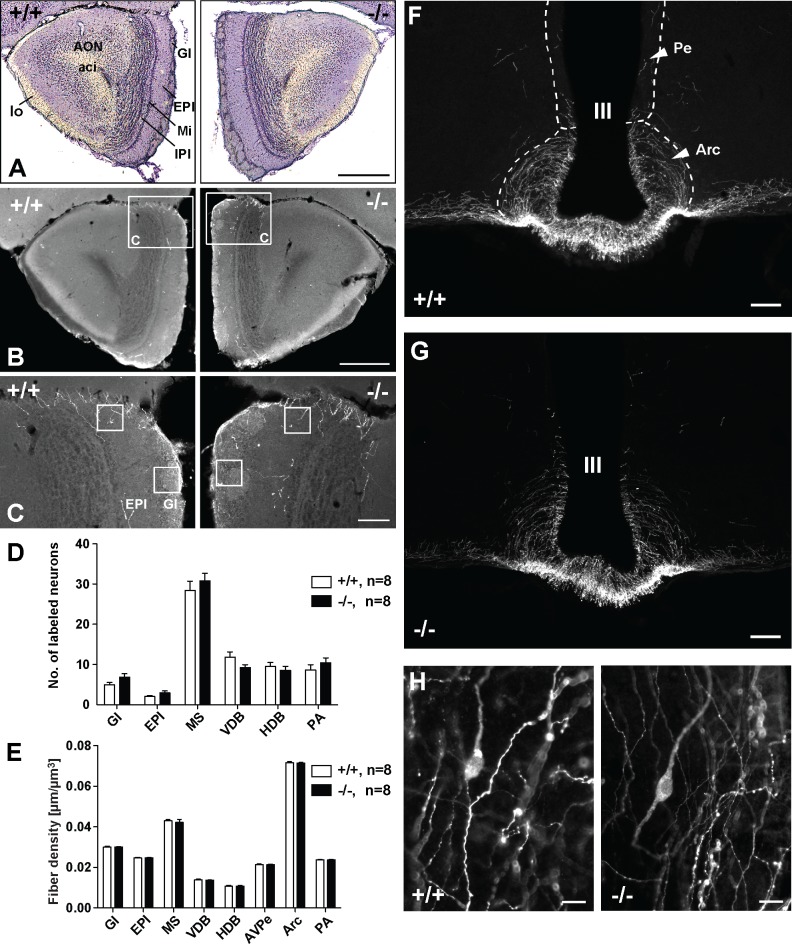
Organization of the olfactory bulb and hypothalamus in wt and Jacob-deficient mice. (A-C) Microphotographs showing the morphology of the olfactory bulb (BO) of wt (+/+) and *Jacob/Nsmf* ko (-/-) mice. (A) Nissl stained sections display the general morphology and subregions of the olfactory bulb (Bregma 2.8 mm, 50x magnification, scale bar 500 μm; Gl, stratum glomerulosum; EPl, stratum plexiforme externum, Mi, mitral cell layer, IPl, internal plexiform layer, AON, anterior olfactory nucleus, aci, anterior commissure, intrabulbar part, lo, lateral olfactory tract). (B) shows sections of both genotypes stained against GnRH (50x magnification, scale bar 500 μm). The frames in (B) indicate the image sections shown in (C). In (C) (200x magnification, scale bar 100 μm) the sample fields are marked, which were used to analyze the fiber densities in Gl and EPl. There are no differences in general morphology between wt and Jacob-deficient mice. (D, E) Quantification of GnRH-IR neurons (D) and fiber densities (E) in brains of wt (+/+) and *Jacob/Nsmf* ko (-/-) mice. (D) The number of GnRH-positive neurons was estimated in the olfactory bulb (Gl, EPl), in the medial septum (MS), ventral diagonal (VDB) and horizontal diagonal band (HDB) of Broca and preoptic area (PA). (E) The density of GnRH-IR fibers was analyzed in the same regions and furthermore in anteroventral paraventricular hypothalamic nucleus (AVPe) and nucleus arcuatus hypothalami (Arc). No significant differences were found between wt and *Jacob/Nsmf* ko mice (data are reported as mean ± SEM, two-way repeated measures ANOVAs were performed using REGION as within-subject factor and MOUSE LINE as between-subject factor. Post hoc analyses were performed using unpaired t-tests (Welch’s test) with Bonferroni-Holm adjustment). (F-H) Microphotographs showing the distribution of GnRH-IR neurons and fibers in the hypothalamic area of wt and *Jacob/Nsmf* ko mice. (F) and (G) display the periventricular (Pe) and arcuate hypothalamic nuclei (Arc) around the third ventricle (III) (Bregma -1.70 mm, scale bars are 100 μm). (H) shows exemplary GnRH-IR neurons in the medial preoptic area (PA) of wt and *Jacob/Nsmf* ko mice (Bregma 0.5 mm, scale bars are 20 μm).

Along these lines we found that the general organization of the bulbus olfactorius is normal in *Jacob/Nsmf* ko mice (Fig 3A–3C). No gross abnormalities in cell distribution and layering as well as the number of GnRH-immunoreactive (IR) neurons and fiber density in stratum glomerulosum (Gl) and stratum plexiforme externum (EPl) of the olfactory bulb were apparent (Fig 3A–3D).

### The number of GnRH-positive neurons and fibers are normal in the brain of *Jacob/Nsmf* ko mice

A hallmark of KS is a migration deficit of GnRH-expressing neurons from the olfactory bulb to the hypothalamus and a reduced number of GnRH-immunopositive fibers in the hypothalamus. In order to determine if *Jacob/Nsmf* ko and wt littermates show differences in markers of the hypothalamic-pituitary-gonadal axis we investigated both genotypes for the number of GnRH-IR neurons in Gl and EPl of olfactory bulb (Fig 3A–3C), in the medial septum (MS), ventral diagonal (VDB), horizontal diagonal band (HDB) of Broca and preoptic area (PA) (Fig 3F–3H and 3D for quantification). A two-way repeated-measures ANOVA showed no significant interaction of REGION and GENOTYPE (F(1.90, 25.54) = 1.49, p>0.05) and no significant effect of the between-subject factor GENOTYPE (F(1,14) = 0.41, p>0.05). Thus, *Jacob/Nsmf* ko and wt mice show a comparable number of GnRH-IR neurons and no difference in the number of GnRH-expressing neurons in the hypothalamus. In addition GnRH-IR fiber densities were estimated in Gl, EPl, MS, VDB, HDB, PA, anteroventral paraventricular hypothalamic nucleus (AVPe) and nucleus arcuatus hypothalami (Arc) (Fig 3E). Again, a two-way repeated-measures ANOVA showed no significant interaction for the region and genotype examined (F(1.80, 25.17) = 0.16, p>0.05) and also no significant effect of the genotype on these measures (F(1,14) = 0.47, p>0.05).

### General brain morphology is not severely altered in Jacob-deficient mice and ko mice show no major abnormalities in general behavior

We next asked whether a *Jacob/Nsmf* gene knockout affects brain organization and behavior. Nissl staining of mice brain sections revealed no obvious differences and indications of gross abnormalities like for instance in the hippocampus between both genotypes (S4 Fig). MRI of the intact brain, however, revealed a small but statistically significant increase in the volume of the striatum (S5 Fig). The volume of other brain regions was not affected by the genotype (S5 Fig).

Jacob-deficient mice did not exhibit clear signs for visual and auditory sensory deficits and motor behavior in the rotarod was not significantly altered in ko animals as compared to wt independently of the sex of the mice. Ko animals seemed to stay longer on the rotarod at slow velocities (S6A Fig). Subsequent behavioral testing in the open field revealed a hyperactive phenotype of ko mice compared to wt littermates independent of the sex. Within the 15 min test period in the open field *Jacob/Nsmf* ko mice spent significantly more time with locomotor activity (distance and speed, S6B and S6C Fig) than wt mice. A social interaction test showed no significant behavioral differences, presenting only a trend towards a more aggressive behavior and more frequent anogenital sniffing in *Jacob/Nsmf* ko mice compared to wt mice (S2 Table). Taken together the data indicate that *Jacob/Nsmf* ko mice are hyperactive when tested in the open field but no apparent sensory-motor deficits were found.

### *Jacob/Nsmf* ko mice show impairments in hippocampus-dependent learning and hippocampal CA1 LTP

**Fig 4:**
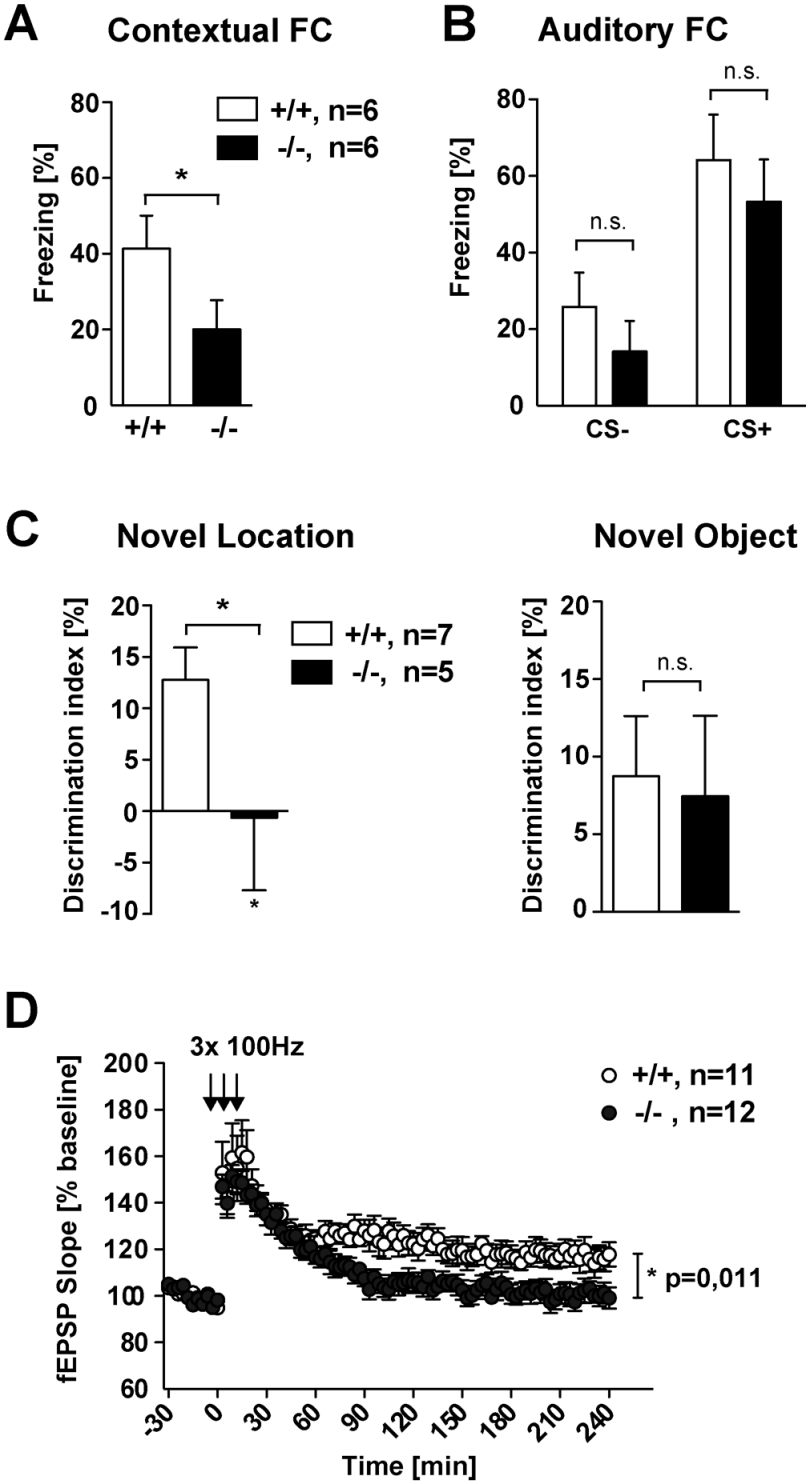
*Jacob/Nsmf* ko mice show impairments in hippocampus-dependent learning and have impaired hippocampal CA1 LTP. (A, B) Contextual fear retrieval (A), but not auditory fear retrieval (B), is impaired in *Jacob/Nsmf* ko mice (n = 6 for each genotype, Student’s t-test, *P<0.05, values are mean + SEM). (C) Novel object location recognition but not novel object recognition is impaired in *Jacob/Nsmf* ko mice. Discrimination index for the novel object location is reduced in *Jacob/Nsmf* ko mice when compared to wt mice. *Jacob/Nsmf* ko mice performed as good as wt mice in the novel object recognition test (n = 7 wt, n = 5 ko, *P<0.05, values are mean + SEM). (D) Expression of LTP after 3 times high frequency stimulation (HFS) trains lasting 1s at 100Hz was impaired in *Jacob/Nsmf* ko mice in comparison to wt mice (*p = 0.011 < 0.05, data are represented as mean ± SEM).

We therefore next addressed whether conditioned behavior and cognitive function might be impaired in Jacob-deficient mice. To this end, we tested mice of both genotypes in auditory-discrimination learning, a cortex-dependent learning task [18], and contextual fear conditioning, a behavioral paradigm that is sensitive to alterations in hippocampal circuitry and function [19]. Interestingly, *Jacob/Nsmf* ko mice exhibited normal auditory discrimination learning with a normal learning curve and performance over the course of the experiment (S7 Fig). In stark contrast, Jacob-deficient mice were clearly impaired in contextual fear conditioning, a learning task that has been associated with the induction of Hebbian plasticity at hippocampal CA1 synapses [19] (Fig 4A). Cued fear conditioning, that is independent of hippocampal function [19], was not affected in ko mice (Fig 4B). Furthermore *Jacob/Nsmf* ko mice were also clearly impaired in object recognition memory (Fig 4C), a behavioral paradigm that is also sensitive to hippocampal dysfunction.

In previous work we found that Jacob rapidly translocate to the nucleus after induction of NMDAR-dependent LTP but not long-term depression (LTD) at hippocampal CA1 synapses [20]. We therefore tested here whether *Jacob/Nsmf* ko mice exhibit normal Schaffer collateral LTP. In these experiments, we found a clear decay of the field excitatory postsynaptic potential (fEPSP) slope already 30–40 minutes after the induction of LTP (Fig 4D), although basal synaptic transmission (S8A Fig), paired-pulse facilitation and the input-output curve were not shifted as compared to wt controls (S8B and S8C Fig). Late phase LTP was almost absent in *Jacob/Nsmf* ko mice despite a similar induction of early LTP like in wt mice (Fig 4D). Collectively, these data indicate that deletion of the *Nsmf* gene in development results in an impairment of hippocampal function and reduced synaptic plasticity of hippocampal CA1 synapses in adulthood.

### *Jacob/Nsmf* ko mice exhibit hippocampal dysplasia

**Fig 5:**
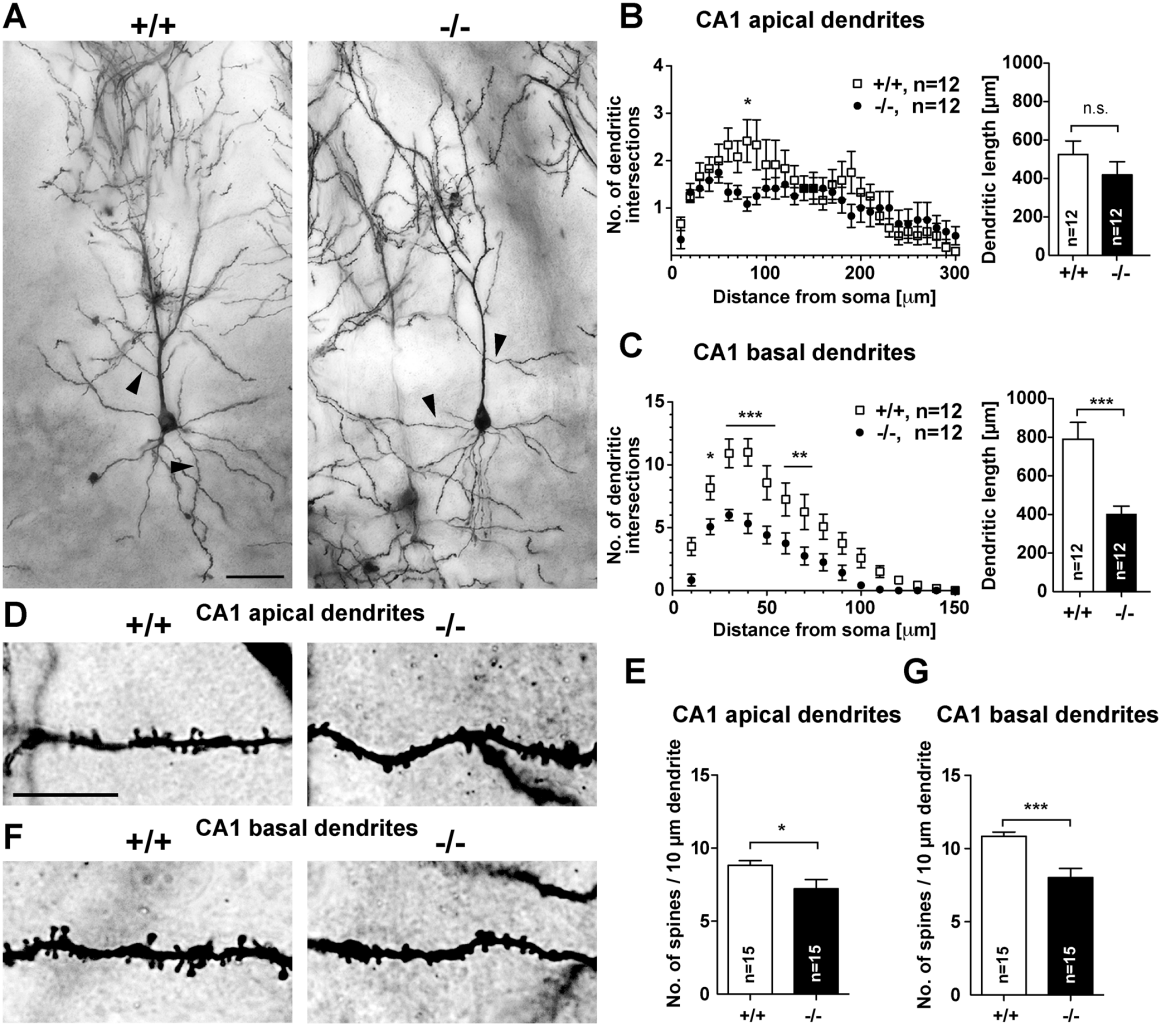
Morphology of Golgi-stained pyramidal neurons in the hippocampal CA1 region of wt and *Jacob/Nsmf* ko mice. (A) Dendritic branching was examined using Sholl analysis in wt (+/+) and ko (-/-) pyramidal neurons (n = 12 neurons, 2–4 slices of 4 adult, 12 weeks old animals were analyzed). (B) Apical dendrites of ko mice seem to be less branched compared to wt mice although the entire dendritic lengths do not differ significantly. (C) Basal dendrites of *Jacob/Nsmf* ko mice are significantly less branched, i.e. have fewer intersections per Sholl-segment in particular 10–110 μm apart from the soma compared to wt mice. Also, basal dendrites of ko mice are significantly shorter in CA1. Arrowheads in A correspond to enlarged images D and F. (D, F) Enlarged areas showing the dendritic spine distribution and morphology of Golgi-stained pyramidal neurons in the CA1 region (apical dendrites D, E, and basal F, G) in wt (+/+) and *Jacob/Nsmf* ko mice (-/-). Both second-order apical (E) and basal (G) dendrites of ko mice bear less spines compared to wt mice. Scale bar in A is 50 μm, scale bar in D is 20 μm. Data are represented as mean ± SEM (ANOVA or Student´s t-test, * p<0.05, **p<0.01, ***p<0.001).

We next wondered whether the observed functional deficits in adult mice could be a result of structural alterations and therefore analyzed synapto-dendritic cytoarchitecture of CA1 pyramidal neurons using the Golgi-Cox method. A subsequent Sholl analysis of Golgi-Cox stained cells revealed a clear reduction of dendritic complexity in CA1 neurons of *Jacob/Nsmf* ko mice (Fig 5). Apical and more prominently basal dendrites were affected (Fig 5A–5C). Jacob-deficient mice exhibit shorter dendrites and much lower number of branches for a given dendritic segment. This rather prominent phenotype prompted us to investigate the density of spine synapses. Interestingly, spine density was clearly reduced in basal and apical dendrites in secondary branches of CA1 neurons (Fig 5D–5G). Further evidence for structural deficits in hippocampal development in the *Jacob/Nsmf* ko mice came from TIMM-staining that revealed an enlargement of the dentate gyrus (DG) projection as compared to wt mice (S9A–S9E Fig). In addition, we found an altered catecholaminergic innervation of the hippocampus with a clear increase in the number of tyrosine-hydoxylase positive fibres in the CA3 but not CA1 region or DG in *Jacob/Nsmf* ko mice (S9F–S9J and S10 Figs). In contrast, catecholaminergic innervation in the striatum and ventral tegmental area was normal in ko mice compared to wt littermates as shown by tyrosine-hydroxylase staining (S11 Fig). Thus, the analyses suggest that structural abnormalities in the hippocampus might underlie the functional deficits observed in adult *Jacob/Nsmf* ko mice.

### Mouse hippocampal primary neurons from *Jacob/Nsmf* ko mice show a simplified dendritic tree and a reduced number of spines during neuronal development

**Fig 6:**
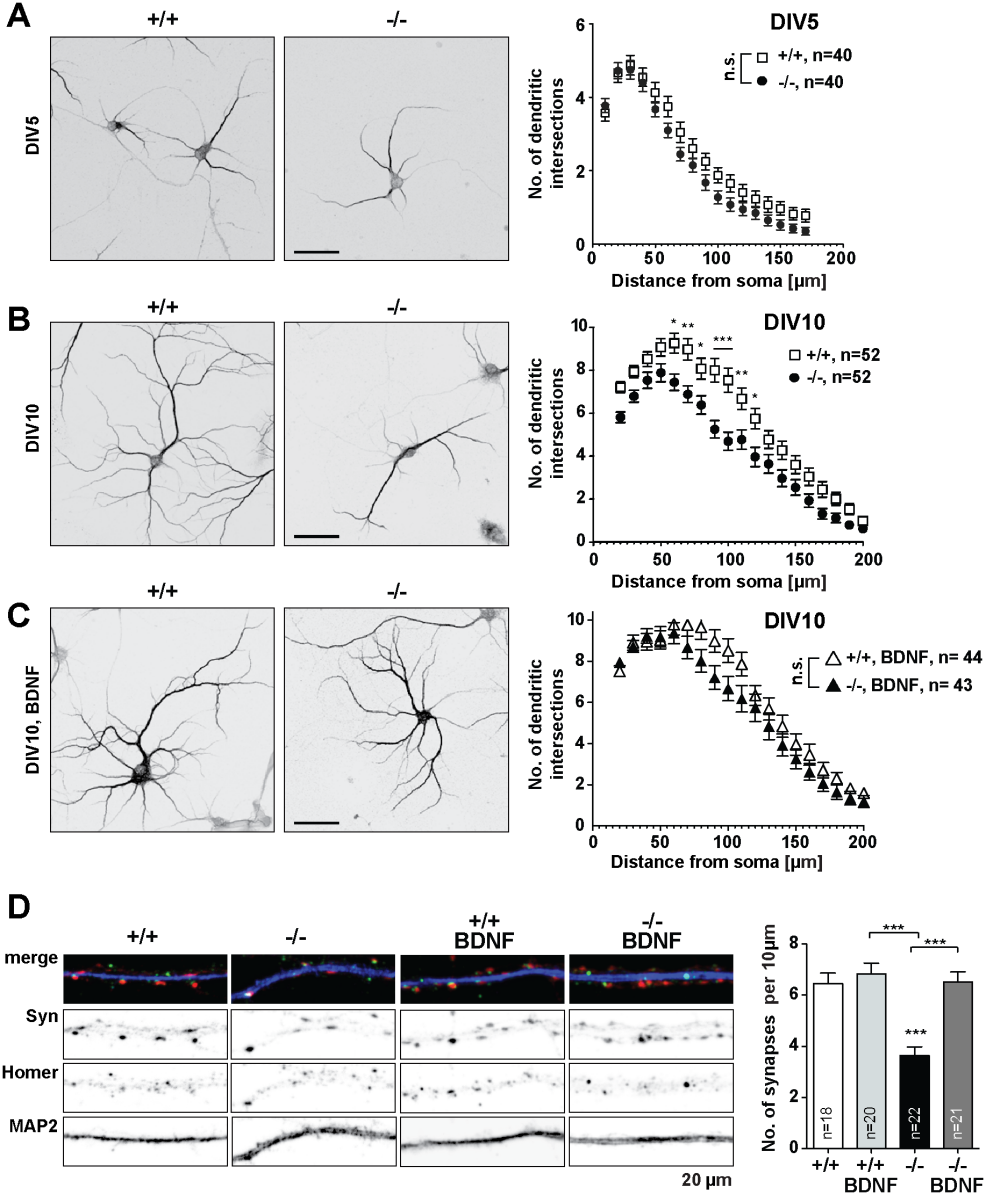
*Jacob/Nsmf* ko mouse hippocampal neurons display a simplified dendritic tree which is rescued by chronic BDNF (100 ng/ml) application (at DIV2 and at DIV6). (A, B) Representative micrographs of wt and *Jacob/Nsmf* ko hippocampal neurons immunostained with MAP2 at (A) DIV5 and (B) DIV10. For Sholl analysis the number of dendritic intersections of wt and *Jacob/Nsmf* ko hippocampal neurons was plotted against the distance. (A) At DIV5 wt and *Jacob/Nsmf* ko hippocampal neurons display no difference in the arborization (n = 40 for wt and n = 40 for *Jacob/Nsmf* ko). (B) At DIV10 *Jacob/Nsmf* ko hippocampal neurons display a simplified dendritic tree as compared to wt neurons. A two-way repeated measures ANOVA revealed that there was both a main effect of genotype F(1,102) = 16.41, p<0.001, ηp2 = 3.5, as well as a main effect of distance F(1,18) = 152.2, p<0.001, ηp2 = 44.37. In addition, the interaction of these two variables was also significant (F(1,18) = 2.27, p = 0.002, ηp2 = 0.66). Post hoc Bonferroni tests showed that there are significant differences in dendritic arborization regarding distances at 60 μm away from the soma p<0.05; at 70 μm, p<0.01; at 80 μm, p<0.05; at 90 μm, p<0.001; at 100 μm, p<0.001; at 110, p<0.01; at 120, p<0.05. (C) Chronic application of BDNF (100 ng/ml, DIV2 and DIV6) rescues the dendritic defect of *Jacob/Nsmf* ko hippocampal neurons, as there is no significant difference when compared to wt neurons. (D) *Jacob/Nsmf* ko neurons display a reduced number of synaptic contacts as compared to wt controls. Representative micrographs of DIV15 wt and *Jacob/Nsmf* ko hippocampal neurons primary distal dendrites immunostained with MAP2 (blue), Homer1 (green) and Synaptophysin (Syn, red), following treatment with BDNF (100 ng/ml) at DIV 2 and DIV 6. Co-localization of synaptic puncta per 10 μm was quantified. Chronic BDNF treatment of *Jacob/Nsmf* ko neurons rescues the synaptic phenotype observed, as no significant differences appear between wt controls and the BDNF-treated groups. Two-way repeated measures ANOVA with Bonferroni posttest revealed that there was both a main effect of genotype F(1,77) = 15.48, p<0.001 as well as a main effect of treatment F(1,77) = 17.10, p<0.001. ***p<0.001. Scale bars in A, B, C = 50μm. Dendritic segment in D is 20μM.

The data outlined above suggest that neuronal development of hippocampal pyramidal neurons might be compromised in *Jacob/Nsmf* ko mice. To further confirm this phenotype we investigated in the next set of experiments dendrite development and synaptogenesis in mouse hippocampal primary cultures. A Sholl analysis revealed that dendrite development is also impaired in hippocampal primary neurons deriving from *Jacob/Nsmf* ko mice. In comparison to wt controls, Jacob-deficient neurons exhibited a shorter neurite length and less branching of Microtubule-associated protein 2 (MAP2)-positive dendrites at day *in vitro* (DIV) 10 but not at DIV 5 (Fig 6A and 6B). Moreover, the number of synaptic contacts was reduced in later development (DIV15) in *Jacob/Nsmf* ko neurons as evidenced by staining with the postsynaptic marker Homer1 and the presynaptic marker Synaptophysin (Fig 6D). Thus, synaptogenesis is impaired in hippocampal primary neurons devoid of Jacob. Interestingly, a similar analysis of cortical primary neurons from wt and *Jacob/Nsmf* ko mice revealed no significant differences in dendrito- and synaptogenesis between both genotypes (S12 Fig).

### BDNF mRNA and protein levels are significantly lower in *Jacob/Nsmf* ko mice during dendritogenesis and BDNF promoter activity is attenuated in primary mouse hippocampal *Jacob/Nsmf* ko neurons

**Fig 7:**
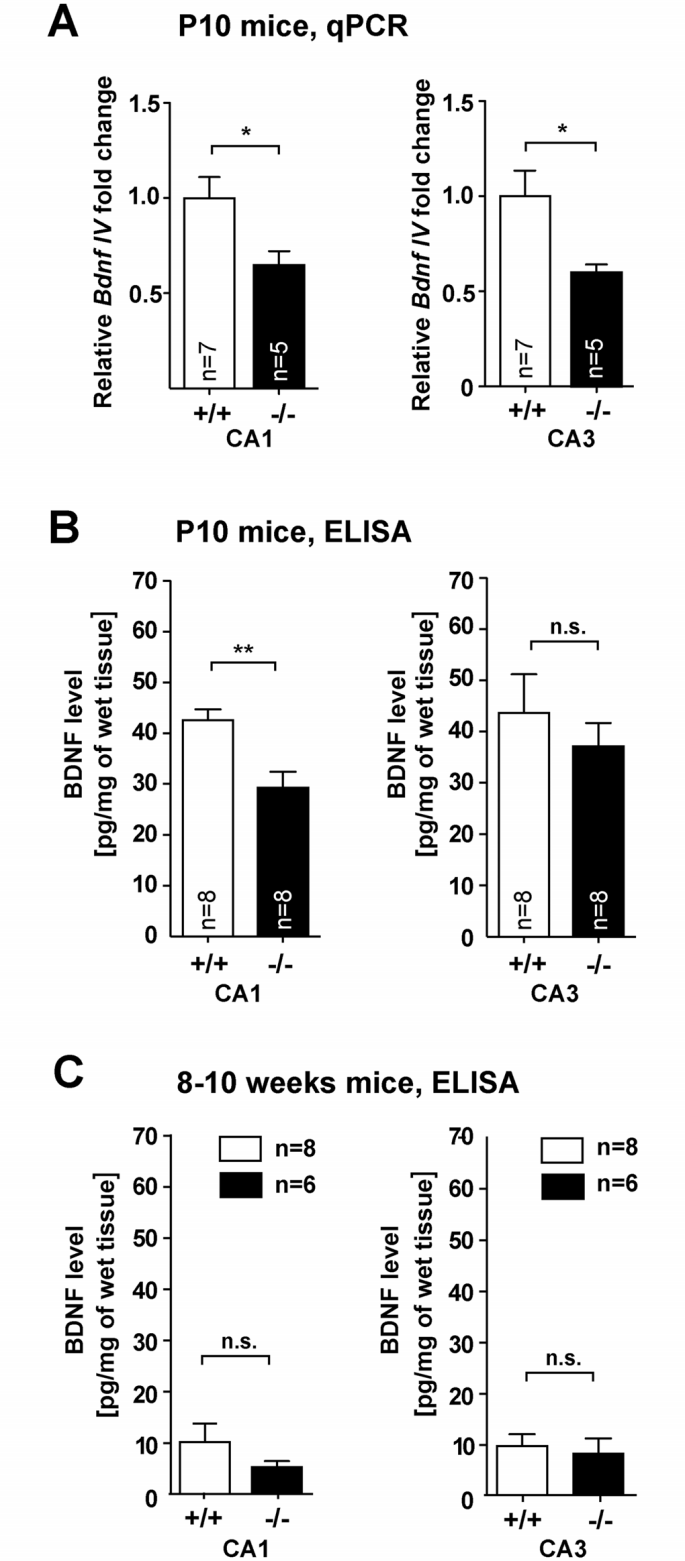
Subregion- and age-dependent changes in *Bdnf* mRNA expression and BDNF protein levels in *Jacob/Nsmf* ko mice. (A) Transcript levels of *Bdnf* exon IV in CA1 and CA3 regions of hippocampus in P10 *Jacob/Nsmf* ko (-/-) mice exhibit a decrease compared to wt (+/+) mice. (B, C) BDNF-ELISA analysis of CA1 and CA3 tissue samples from P10 and 8–10 weeks old (B) *Jacob/Nsmf* ko (-/-) and wt (+/+) mice revealed significant lower levels of BDNF only in CA1 region of P10 mice. (C) The expression levels for both regions in mice at the age of 8–10 weeks are similar. (*p < 0.05, **p < 0.01, two-tailed unpaired t-test, values represent mean ± SEM).

Collectively these data point out a profound hippocampal dysplasia and impaired synapto-dendritic development in *Jacob/Nsmf* ko mice. We therefore sought to unravel the underlying mechanisms. In previous work we have been able to show that Jacob regulates transcriptional activity of CREB and CREB-dependent *Bdnf* gene transcription [2]. BDNF has been implicated in spinogenesis and dendrite development [21]. We therefore proceeded to examine hippocampal mRNA and protein levels of BDNF in wt and *Jacob/Nsmf* ko mice during development. Quantitative PCR experiments revealed decreased *Bdnf* exon IV expression in CA1 and CA3 regions from samples of P10 *Jacob/Nsmf* ko mice (Fig 7A). ELISA-assays confirmed significantly lower BDNF levels in Jacob-deficient mice at P10 in CA1 and a trend towards reduced levels in CA3 (Fig 7B). CA1 BDNF levels appear to be slightly but not statistically significant reduced in adulthood (Fig 7C). We therefore next asked whether *Bdnf* promoter activity might be differentially regulated in *Jacob/Nsmf* ko neurons at P10. To this end, we performed a reporter gene assay where mouse primary hippocampal neurons were transfected with a GFP construct fused to the *Bdnf-* exon1/2 promoter which contains a Cre-site and is regulated by Jacob [2] and then applied BDNF stimulation. In these experiments we observed that the relative BDNF-induced increase in GFP expression was clearly attenuated in Jacob-deficient neurons as compared to wt neurons (S13 Fig), indicating that a positive feedback loop involved in *Bdnf* gene expression might be interrupted following *Jacob/Nsmf* gene knockout.

### BDNF supplementation to the culture medium rescues the *Jacob/Nsmf* ko phenotype in mouse hippocampal primary neurons

Published work suggests that BDNF might have a positive impact on dendrite development and spinogenesis of hippocampal primary neurons [21]. We next performed *in vitro* experiments in which hippocampal primary neurons derived from *Jacob/Nsmf* ko mice were cultured in the presence of BDNF. Bath application of 100 ng/ml BDNF at DIV2 and DIV6 increased the complexity of the dendritic tree of *Jacob/Nsmf* ko neurons at DIV 10 (Fig 6C) as compared to the ko controls grown without BDNF. No effect of this treatment was seen in control wt neurons (Fig 6C). Similarly, we found an increase in the number of spine synapses following BDNF supplementation at DIV2 and 6 in *Jacob/Nsmf* ko but not in wt neurons at DIV15 (Fig 6D). Control experiments with administration of NGF and boiled BDNF yielded no or very little effect on dendrito- and synaptogenesis (S14 Fig). Of note, quantitative immunoblotting revealed that TrkB as well as pTrkB levels are not altered as compared to wt in hippocampal lysates from adult Jacob-deficient mice (S15 Fig). BDNF application could also not rescue the LTP phenotype in adult *Jacob/Nsmf* ko mice (S16 Fig). Collectively the data suggest that specifically BDNF-signaling pathways appear to be sensitized to BDNF administration in Jacob-deficient neurons in comparison to wt, and enhancing BDNF levels corrects, at least in part, the deficit in synapto-dendritic development in cultured *Jacob/Nsmf* ko neurons.

### BDNF induces nuclear translocation of phosphorylated (p)Jacob in an NMDAR-dependent manner

Previous reports have claimed that Jacob/NELF is a secreted protein that associates with the cell surface of neurons and might function as a guidance molecule [5,6] (but see [16]). To exclude the possibility that Jacob might have an additional function as a guidance molecule on top of synapse-to-nucleus communication that might account for the findings described above we performed live-staining of rat hippocampal primary neurons with a Jacob antibody. These experiments revealed no specific staining above the background signal resulting from the secondary antibody (S17 Fig; see S18 Fig for specificity of the antibody). A positive live-staining control with a prion protein (PrP)-antibody showed extensive labeling of hippocampal primary neurons (S17 Fig). Hence, prominent Jacob immunofluorescence was evident following permeabilization of cells with a conventional TritonX-100 buffer (S17 Fig). Collectively, these data do not support an extracellular localization of Jacob in rat hippocampal primary neurons and rather point to a function of Jacob in NMDAR-to-nucleus signaling, as shown previously [2].

**Fig 8:**
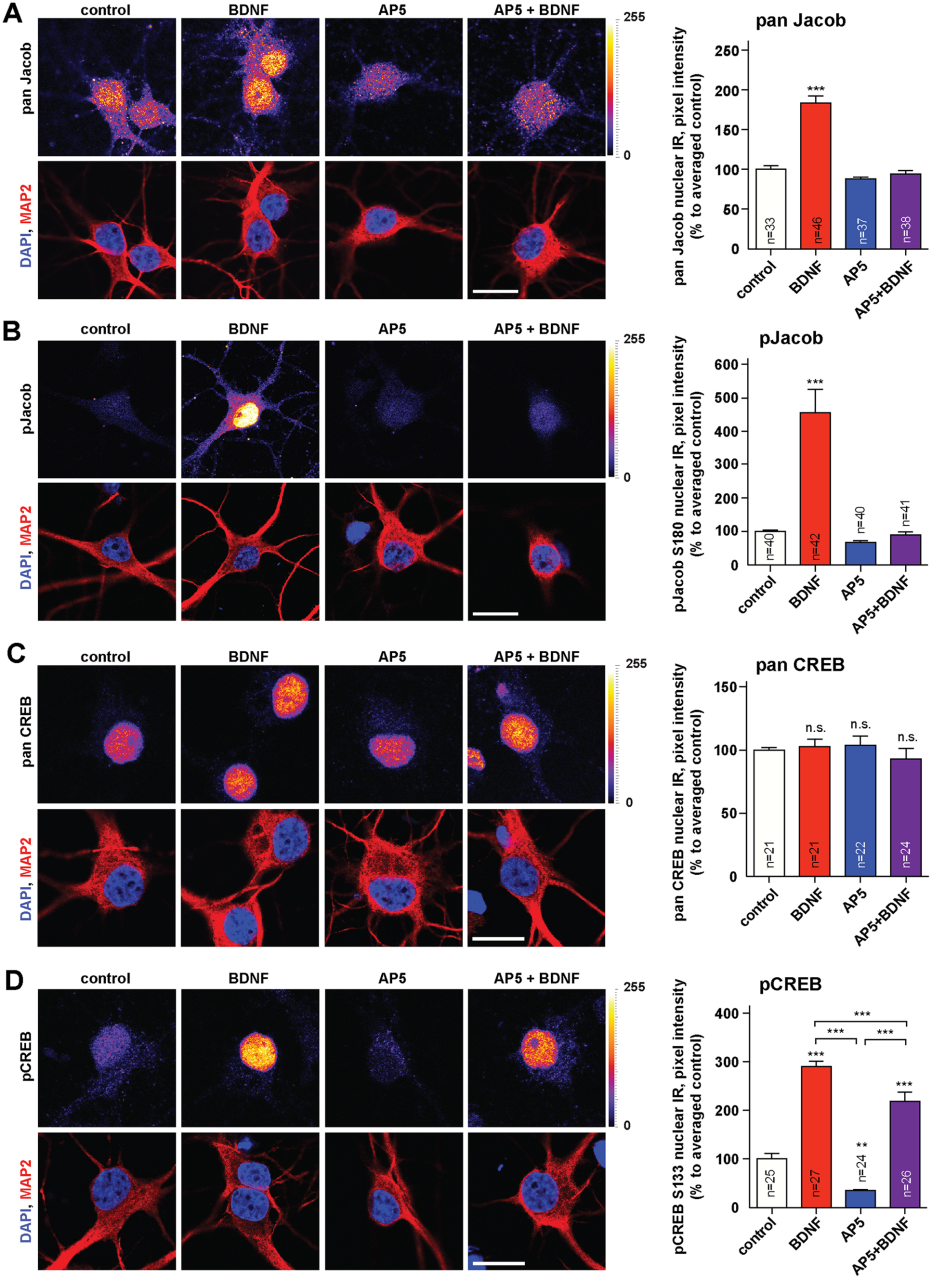
BDNF induces the nuclear import of pJacob and increase in pCREB in a NMDAR-dependent manner. Rat dissociated hippocampal cultures were treated at DIV10 with BDNF, AP5 or both, fixed and stained for panJacob (A), pJacob (B), panCREB (C), and pCREB (D). The BDNF-induced accumulation of pJacob in the nucleus is abolished by NMDAR blocking with AP5. BDNF application caused an increase in nuclear pCREB levels, which is partially abolished by NMDAR blocking (C) with total unchanged levels of nuclear CREB (D). Confocal images averaged from two confocal sections of the nucleus. Lookup table indicates original pixel intensities from 0 to 255. Graphs represent mean +/-SEM staining intensity within nuclear plane normalized to control. One-way ANOVA with Tukey posttest ***p<0.001; **p<0.01, *p<0.05. Scale bar indicate 20μm.

We next wondered whether the low BDNF-levels in *Jacob/Nsmf* ko mice in early development might relate to the nuclear import of the protein and whether exogenous BDNF itself can drive Jacob into the nucleus. In the first set of experiments we found that acute bath application of BDNF (100ng/ml) indeed results in nuclear accumulation of Jacob in rat hippocampal primary neurons at DIV10 and that this increase was completely blocked when the NMDAR antagonist AP5 was co-applied (Fig 8A). In accordance with previous observations, we found a prominent increase in nuclear pJacob immunofluorescence in response to acute BDNF application at DIV10 (Fig 8B). Additionally, BDNF application at DIV 10 substantially increased nuclear pCREB immunoreactivity (Fig 8D) whereas CREB immunofluorescence remained unaltered (Fig 8C). This increase in pCREB was partially blocked by AP5 (Fig 8D). BDNF-induced CREB activation from NMDAR-independent pathways likely accounts for the remaining increase.

### BDNF-induced nuclear increase in pERK/ERK is attenuated in *Jacob/Nsmf* ko neurons and basal pCREB levels are reduced

**Fig 9:**
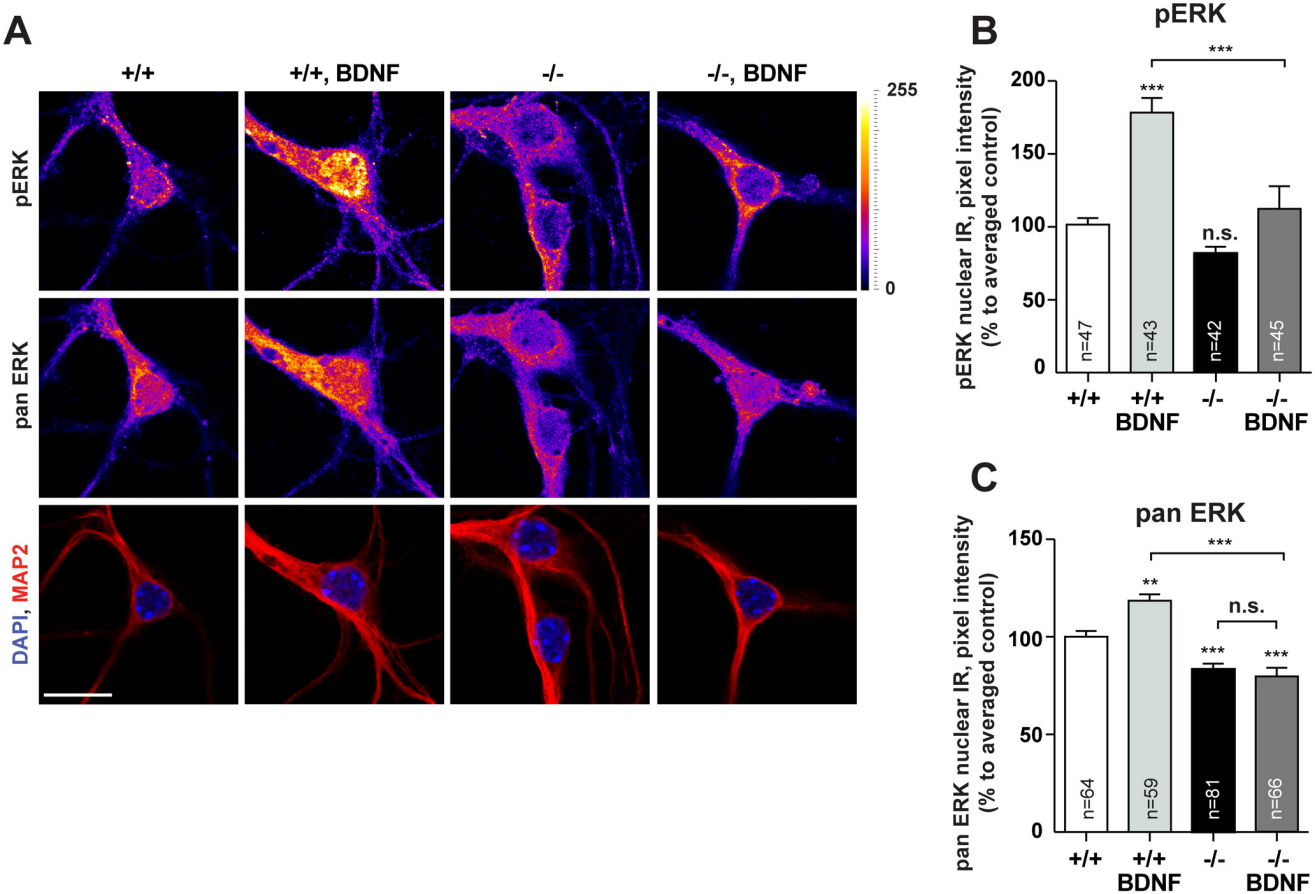
Changes in nuclear levels of pERK and ERK after BDNF treatment in *Jacob/Nsmf* ko and wt hippocampal neurons. (A) DIV 10 hippocampal neurons were treated with BDNF for 30 minutes, fixed and stained for pERK and pan ERK. (B, C) BDNF application caused an increase in nuclear pan ERK and pERK immunofluorescence levels in WT but not *Jacob/Nsmf* ko neurons. Two-way ANOVA with Tukey posttest ***p<0.001; **p<0.01, *p<0.05. Scale bar indicates 20μm.

**Fig 10:**
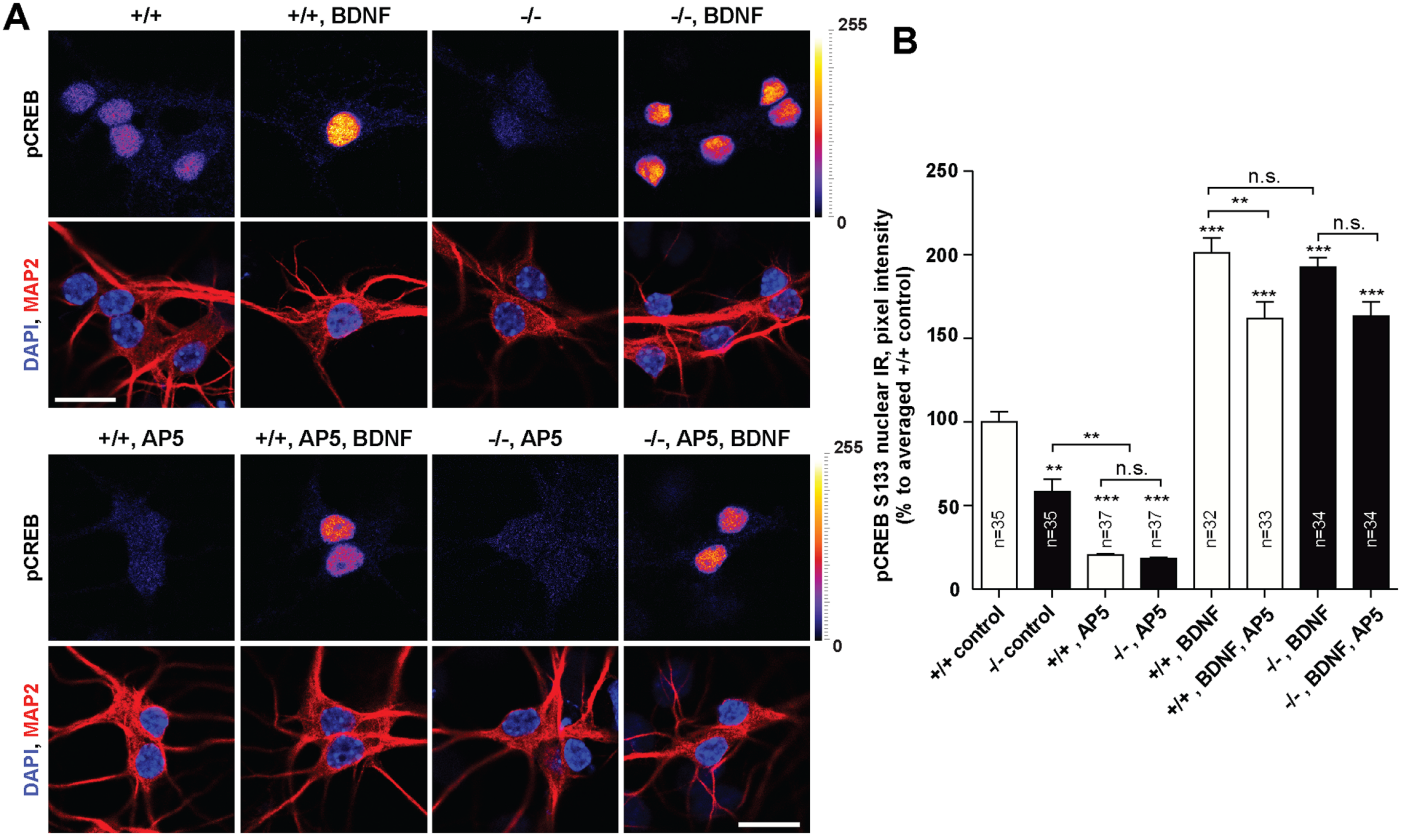
Changes in nuclear levels of pCREB after BDNF treatment in *Jacob/Nsmf* ko and wt hippocampal neurons. (A) DIV 10 hippocampal neurons were treated and stained as indicated. (B) Quantification revealed that in Jacob-deficient neurons the nuclear pCREB level is lower under basal conditions. After application of BDNF, nuclear amounts of pCREB significantly increased in neurons from both genotypes. Interestingly, application of the NMDAR-antagonist AP5 normalizes the differences in nuclear pCREB immunostaining intensity in wt and ko neurons. Co-application of AP5 with BDNF had only in wt neurons a statistically significant effect. Two-way ANOVA with Tukey posttest ***p<0.001; **p<0.01, *p<0.05. Scale bars indicate 20μm.

Collectively these data suggest a scenario where BDNF drives pJacob during dendritogenesis into the nucleus which then results in docking of pERK to the CREB complex [2], increased serine 133 phosphorylation of CREB and CREB-dependent enhanced *Bdnf* gene transcription. Newly synthesized BDNF would then positively feedback to dendrite growth and synaptogenesis and concomitantly increase nuclear import of Jacob. In the next set of experiments we analyzed whether exogenous BDNF application increases pERK and pCREB in the nucleus of hippocampal primary neurons of *Jacob/Nsmf* ko mice. We found that the nuclear expression of pERK 30 minutes after the onset of stimulation with BDNF was significantly blunted in Jacob-deficient neurons at DIV10 following BDNF application for 30 minutes (Fig 9A–9C). Moreover, basal nuclear ERK levels were greatly reduced in Jacob-deficient neurons as compared to wt neurons, indicating a prominent role of Jacob for BDNF-induced nuclear import of ERK/pERK during the development of mouse hippocampal primary neurons (Fig 9A–9C). Additionally, under basal conditions pCREB but not panCREB immunofluorescence levels were significantly reduced in hippocampal neurons devoid of Jacob (Fig 10 and S19 Fig). Application of the NMDAR antagonist AP5 to these cultures normalized the difference in pCREB staining intensity (Fig 10), suggesting that NMDAR in wt neurons are more efficiently coupled to pCREB. Interestingly, however, acute application of BDNF results in an increase of pCREB immunofluorescence that is relative to basal levels even more pronounced in Jacob-deficient than in wt neurons (Fig 10), indicating sensitization of BDNF-triggered signaling pathways coupled to activation of CREB. These pathways appear to be NMDAR-independent since co-application of AP5 resulted in a statistically significant effect only in wt neurons (Fig 10).

## Discussion

In the present study we addressed whether *Jacob/Nsmf* null mutant mice exhibit phenotypes related to KS and investigated the impact of Jacob on neuronal development with emphasis on the hippocampus. We found no evidence for anosmia or a major migration deficit of GnRH neurons in these mice. Sexual development, fertility hallmarks and reproductive capacity appear to be to a large extent normal. The results of the present work are in contrast to a recent report that claims subfertility and impaired puberty in female ko mice [22]. Our detailed analysis of a broad number of reproductive parameters clearly excludes subfertility and hypogonadotropic hypogonadism in both males and females. The reason for this discrepancy is not apparent but the mild phenotypes observed by us in knockout animals (i.e. irregularities in folliculogenesis and in the estrus cycle that might be estradiol driven, lower testosterone levels in male mice) clearly do not result in subfertility.

The findings herein are also incongruent with previous reports claiming that Jacob/NELF acts on growth cones as an extracellular guidance molecule that enables GnRH neurons to reach their destination in the hypothalamus [5,6]. On the ground of the present and previous work [16] it is also unlikely that Jacob/NELF is a secreted molecule. It is therefore tempting to speculate that, if Jacob has any function in neuronal cell migration it will be related to the regulation of gene expression. Interestingly, at later stages of migration, NMDAR activation slows down the migration of GnRH neurons [23] and Jacob has been shown to translocate to the nucleus following NMDAR stimulation [1,2]. However, deficits in cell migration were not apparent in any of the brain regions examined in *Jacob/Nsmf* ko mice and Jacob protein levels are relatively low at birth and prominently increase only in the second postnatal week [1,24]. Moreover, it is important to emphasize that, in recent years, there has been extensive documentation of a high degree of heterogeneity within the GnRH neuronal population and it is unlikely that a single genetic mutation could completely prevent these neurons from reaching their destination [11,25]. Thus, GnRH deficiency in humans might only occur when the majority of GnRH neurons are affected by mutations in more than one gene. Along these lines it has been proposed that mainly digenic mutations in genes that are important for migration of GnRH neurons will result in IHH [9,11,25]. Taken together, the studies performed in null mutant mice provided no evidence for an involvement of Jacob in KS, although the experiments conducted in the present study do not rule out subtle defects in GnRH migration.

The most compelling developmental phenotype that we observed in *Jacob/Nsmf* null mutant mice was hippocampal dysplasia. We could show that Jacob deficiency during development results in shorter dendrites, less dendritic branching, fewer synapses, an increased catecholaminergic innervation, an altered CA3-DG projection as well as reduced pCREB and BDNF levels. In consequence, *Jacob/Nsmf* ko mice show behavioral deficits in contextual fear conditioning and object recognition memory, two hippocampus-dependent learning tasks and impairments of synaptic plasticity in classical NMDAR-dependent Schaffer-collateral CA1 LTP. We surprisingly found no alteration in fEPSP size despite the fact that the number of CA1 synapses is reduced in *Jacob/Nsmf* knockout mice. There are a number of possible explanations for this unexpected finding, which includes a shift in the excitation/inhibition balance, changes in intrinsic excitability and synaptic surface expression of NMDA/AMPA receptors as well as others. Interestingly, BDNF application during tetanization could not rescue late LTP. This might be due to the impaired nuclear import of pERK and reduced CREB-dependent gene expression following activation of synaptic NMDAR. Since this import is already impaired in development it will affect synaptic function permanently. Although no obvious differences in TrkB and pTyr550TrkB levels were found in hippocampi of knockout mice we can therefore not exclude the possibility that synaptic changes occur that are either directly or indirectly related to BDNF/TrkB signaling in Jacob deficient neurons. Thus, the preliminary analysis does not exclude alterations in downstream TrkB signaling and further research should address these issues. In addition, the present study suggests that loss-of-function mutations in the *Jacob/Nsmf* gene are likely to cause selective cognitive dysfunction in humans. Of interest in this regard is a report on hippocampal dysplasia in two cases of KS [26].

Cortex- and amygdala-dependent auditory learning, in contrast, was not significantly affected in the mutants. We therefore focused our mechanistic analysis mainly on hippocampal CA1 pyramidal neurons. Nonetheless it will be interesting to see whether similar structural and functional deficits are also present in other brain regions. Initial experiments with cortical primary neurons revealed no clear-cut differences in dendrito- and synaptogenesis between both genotypes at DIV10 and DIV15. The cortex on the other hand is very heterogeneous in terms of cellular architecture in different cortices and one should be therefore cautious to conclude that the effects of Jacob deficiency are specific for CA1 pyramidal neurons.

The main novel mechanistic insight that we gained from the present work is that the underlying mechanism for the defect in synapto- and dendritogenesis in *Jacob/Nsmf* null mutant mice is probably due to an interrupted positive feedback loop between BDNF-signaling, subsequent nuclear import of pJacob in a complex with pERK, activation of CREB and enhanced BDNF gene transcription. Thus, the present study shows that the previously documented nuclear import of pJacob and subsequent regulation of CREB-dependent BDNF gene expression [2] seems to be also relevant for neuronal development in the hippocampus. Jacob-deficient neurons are more responsive to BDNF application, a fact that is reflected by higher relative increase in pCREB and because BDNF administration normalizes the number of synapses and dendrite complexity at a concentration and treatment regime that has no effect on wt neurons. Interestingly, a previous report has shown that BDNF also regulates dendritic development *via* co-activation of NMDARs, nuclear import of the synapto-nuclear messenger CRTC1 and binding of CRTC1 to CREB in neuronal primary neurons [27]. Thus, long-distance transport of proteins from the plasma membrane to the nucleus and docking to CREB seems to be a more common mechanism.

Three main intracellular signaling cascades are activated by tropomyosin-receptor kinase B (TrkB): the Ras-ERK, the PI3K-Akt and the PLCγ-Ca^2+^ pathway [28]. BDNF-induced nuclear import of Jacob requires co-activation of NMDAR and crosstalk with TrkB receptors and one possibility is that co-activation might directly result in sustained ERK-activation and thereby enhanced nuclear trafficking of Jacob [2]. Jacob preferentially associates with GluN2B-containing NMDAR and nuclear import essentially requires activation of this receptor subtype [1,2]. On the postsynaptic side a functional interaction of BDNF/TrkB signaling with the GluN2B subunit of NMDAR is well documented and this link might depend on activation of Src-family tyrosine kinases by PI3K-Akt [29–31]. The C-terminus of GluN2B contains a clathrin adaptor AP-2-binding site and the internalization motif YEKL [32–34]. The tyrosine 1472 within the YEKL motif of GluN2B is phosphorylated by the Src family of kinases [35,36], and phosphorylation of Y1472 inhibits the binding of AP-2 and promotes surface expression of GluN2B [32,36]. BDNF and GluN2B-containing NMDARs contribute to long-term synaptic potentiation [37,38] and the BDNF/TrkB enhancement of GluN2B signaling is likely dependent on CaMKII phosphorylation, CaMKII/GluN2B binding [39,40] and subsequent AMPA receptor modifications [41–46] as well as activation of ERK [47]. It is tempting to speculate that in *Jacob/Nsmf* mutant mice this signaling might be affected in adulthood.

Finally, the role of BDNF in dendritogenesis is rather controversial also because different dose and administration regimens were used and different cell types and developmental stages were analyzed. Exogenous application of BDNF has been shown in most studies to promote dendrite outgrowth in development and spine density and morphology in mature primary neurons (reviewed in [21,37]). However, while a complete gene knockout is lethal heterozygous, *Bdnf* +/-, mouse mutants with reduced brain BDNF levels show only a mild phenotype [37,38] [48,49]. The analysis of conditional gene targeted mouse lines has revealed that the morphological effects of a *Bdnf* gene ko are relatively mild if one compares these effects to those of exogenous BDNF application *in vitro* [50–58]. Rauskolb et al. [58] reported a modest dendritic phenotype of CA1 pyramidal neurons in conditional *Bdnf* -/- mice, that was much less prominent than those observed in the striatum in the same study. Apart from methodological differences it is plausible that the complete knockout of the *Bdnf* gene in neurons already very early in neuronal development might have a different effect as compared to the situation in the present work. The Jacob pathway only comes into play later in postnatal development when Jacob levels start to increase. Thus, it is possible that wiring of hippocampal circuitry in the absence of BDNF is different than in the presence of lower levels in a restricted time window. Compensatory mechanisms are plausibly different in both scenarios and it should also be emphasized that altered gene expression in *Jacob/Nsmf* ko mice might not only affect BDNF levels but also other factors involved in dendritogenesis. Another issue is a possible sensitization to BDNF signaling in the mice that might account for the compensation seen after BDNF application in primary neurons. Along these lines TrkB signaling might be altered in Jacob null mutant mice and it is very likely that Jacob will have a synaptic function (see above). Moreover, interruption of the positive feedback loop that we describe in the paper might interfere with synaptogenesis independent of dendritogenesis and will likely not account for all deficits in the null mutant mice.

In summary, we could show that Jacob regulates dendrite growth in hippocampal pyramidal neurons in CA1 and that protein transport of Jacob from TrkB/NMDAR to the nucleus in development is part of a positive feedback loop that promotes both dendrito- and synaptogenesis. Later in development Jacob's nuclear transport is probably under increasing control of synaptic NMDAR as compared to earlier time points when the number of spines is low and we assume that the functional deficits in adult mice at least in part reflect dysplasia and structural deficits in CA1 that are imposed by a gene knockout early in development.

## Materials and Methods

### Ethics statement

All experiments were carried out in accordance with the European Communities Council Directive (2010/63/EU) and approved by the local authorities of Sachsen-Anhalt/Germany / Regierungspräsidium Halle Sachsen-Anhalt/Germany (reference number 42502-2-987IfN).

### Animals

All animals used in this study were bred and maintained in the animal facility of the Leibniz Institute for Neurobiology, Magdeburg, Germany. Animals were housed in groups of up to 5 in individually ventilated cages (IVCs; Green line system, Tecniplast) with free access to food and tap water under controlled environmental conditions (22° +/- 2°C, 55% +/- 10% humidity, 12h, light—dark cycle, with lights on at 06:00 a.m.).

### Generation of *Jacob/Nsmf* ko mice

*Jacob/Nsmf* ko mice were generated with help from Ozgene Pty. For conditional targeting exons 1 to 3 of the *Jacob/Nsmf* locus (GenBank ID: 56876; RefSeq NM_001039386.1; NP_001034475.1) were flanked with loxP sites for Cre-mediated deletion *via* the design of two loxP arms (0,7 and 0,9 kb). The 5´loxP site was inserted into the 5´UTR of exon 1 whereas the 3´loxP site was inserted downstream of exon 3 to ensure deletion of the first 3 exons. Three loxP sites were used to reduce the size of the regions being floxed on either side of the PGK-Neomycin selection cassette (see S1 Fig).

*Jacob/Nsmf* constitutive ko mice were generated using standard procedures from targeted C57BL/6-derived *Bruce-4* embryonic stem (ES) cells. For removal of the Neo cassette flanked by two FRT sites mice were bred to a FLPe recombinase line (Ozgene) resulting in wt/Jac-loxPΔneo/Flp mice. *Jacob/Nsmf* constitutive ko mice were obtained after breeding wt/Jac-loxPΔneo/Flp mice to a CMV-Cre deleter line (Ozgene) for excision in all tissues. In further crossing steps to wt mice the FLPe and Cre transgenes were removed. All breeding steps were performed with mice on C57BL/6J background. For most of the experiments mice from heterozygous breeders were used. Further information can be found in S1 Methods.

### Analysis of mouse fertility parameters

To compare the litter size of Jacob-deficient breeders with heterozygous and wt breeders four males of each genotype were mated with the corresponding females. The mean litter sizes (± SEM) between genotypes were compared over a period of 15 weeks. For analysis of the Mendelian ratio the genotypes of 573 animals (offspring of heterozygous breeding pairs, both sexes) were compared. Body weights of *Jacob/Nsmf* ko, heterozygous and wt littermates were taken after weaning (20–22 days) and at the age of four month.

#### Analysis of the estrous cycle

Virgin wt (n = 18), *Jacob/Nsmf* heterozygous (n = 16) and homozygous (n = 18) females mice were analyzed for the time duration of each phase of their estrous cycle. Therefore, vaginal lavage was performed from all three groups with 50 μl sterile PBS (PAN-Biotech) every day at the same time for two consecutive cycles. The murine estrous cycle lasts for approximately four days and can be divided into four different phases: diestrus, proestrus, estrus (receptive phase) and metestrus. Each phase was defined by its specific cellular composition and appearance under an inverse light microscope as described elsewhere [59] (Zeiss Axiovert 40C).

#### Analysis of ovarian morphology in the estrus phase

Virgin wt (n = 5), *Jacob/Nsmf* heterozygous (n = 4) and ko (n = 5) female mice were sacrificed by cervical dislocation in the estrus phase of the estrous cycle. Ovaries were removed from all animals, fixed in ethanol and embedded in paraffin. Paraffin cuts (5 μm) were performed from entire ovarian tissue. For hematoxilin and eosin staining tissue slides were dewaxed, rehydrated, stained with the corresponding solution, dehydrated, treated with xylol and embedded in Roti-Histokitt II (Roth) embedding medium. Morphological analysis was performed under an inverse light microscope (Zeiss Axiovert 40C). To determine the quantity of follicles and corpora lutea, entire ovarian tissue was analysed and the number of follicles and corpora lutea was compared between all genotypes. Representative pictures were taken from ovaries of each genotype.

#### Enzyme-linked-immunosorbent assay (ELISA) to determine hormone plasma levels of male and female mice

Virgin wt, *Jacob/Nsmf* heterozygous and ko females were sacrificed in different estrous cycle phases; diestrus (n = 3), proestrus (n = 3), estrus (n = 8) and metestrus (n = 3). Blood was obtained by retroorbital puncture and collected in heparinized tubes. After a centrifugation step the plasma was separated from the cellular part and stored at -80°C until hormonal analysis by ELISA. Progesterone plasma levels were determined using the Progesterone ELISA KIT (rat/mouse, DRG Instruments). Estradiol and Luteinizing hormone plasma levels were determined using the E2 ELISA KIT and LH ELISA KIT (USCN Life Science, Hölzel diagnostic). Testosterone plasma levels were determined from serum of adult male wt and *Jacob/Nsmf* ko mice using the Testosteron ELISA KIT (rat/mouse) (DRG Instruments). All steps were performed according to the instructions of the manufacturer.

#### Testicular weights and morphology

Testes were prepared from *Jacob/Nsmf* ko mice (n = 7), heterozygous (n = 9) and wt (n = 7) littermates at the age of 3–4 month and weights were taken. For testicular morphology samples were obtained from *Jacob/Nsmf* ko mice and wt littermates (n = 2) used for social interaction and odor experiments. Testes were immediately fixed in 4% paraformaldehyde, incubated in distilled water, thereafter in an ascending alcohol series and finally embedded in paraffin. Samples were cut into 5 μm thick sections on a HM 355S rotation microtom (Microm International GmbH), mounted on glass slides (Super Frost Plus, ThermoScientific) and subsequently stained for hematoxylin and eosin (Sigma Aldrich) to enable histomorphological analysis.

### Behavioral tests

For behavioral experiments different cohorts of mice were used, as defined in the corresponding paragraphs. However, Rotarod and Open Field test were performed with the same group of mice of both sexes (n = 12 ko, n = 12 wt, half of each sex) at the age of 4 month. Secondly, in odor exposure and social interaction test one group of male *Jacob/Nsmf* ko mice (n = 16) and wt littermates (n = 15) at the age of 4–5 months was used. One week prior to behavior experiment animals were separated and further kept individually.

#### Fear conditioning task

Fear conditioning took place in a conditioning chamber (TSE-Systems, Bad Homburg, Germany) and was performed according to Bergado-Acosta and coworkers [60], with minor modifications. Briefly, the task consisted of 2 adaptation, 2 training, contextual fear retrieval and auditory fear retrieval sessions. Adaptation consisted of presenting a set of three acoustic stimuli (2.5 kHz sinus tone; 85 dB SPL; 10 s; spaced at 30 s inter-stimuli intervals, ISIs) used as CS-. Twenty-four hours later animals were trained in the apparatus, where they received three conditioned stimuli (CS+, 10 kHz sinus tone; 85dB SPL; 9 s; with 30 s ISIs) that were followed by an unconditional stimulus (US) electrical foot shock (1 s; scramble 0.4 mA). On the next day animals were placed in the apparatus for 6 minutes and long-term contextual fear memory retrieval was assessed. On the next day, in order to test cued fear memory retrieval, animals were placed in a different context and after 2 minutes were presented with 4 CS- (30 s ISIs) and 4 CS+ (30 s ISIs), and left for additional 2 minutes before returning to the home cage. Freezing duration (≥ 1 s) was determined using infrared (LED; at x, y and z axis) light-barriers 14 mm apart (sampling rate of up to 100 Hz; TSE-Systems). All behavioral experiments and data analyses were done by an experimenter blind to the genotype.

#### Novel object location and novel object recognition

Object recognition task was performed in a square arena (50 x 50 x 50 cm) under bright light conditions. The objects used were plastic mounting bricks and throughout the experiments objects were used in a counterbalanced manner. Chambers and objects were thoroughly cleaned with 10% ethanol before and after each animal was tested. On the first day training session took place, where animals were exposed to two equal objects (object A). Twenty-four hours later, novel object location test was performed, with one of the familiar objects displaced to a novel location in the arena. On the next day, novel object recognition was performed, were one of the familiar objects (i.e. object A) was replaced by a novel object (i.e. object B). Exploration was video-recorded in all 3 sessions using ANY-maze software (ANY-maze 4.50, Stoelting Co. Wood Dale). Exploration was considered only when the animal touched or reached the object with the nose at a distance of less than 2 cm. The time mice spent exploring the familiar and the novel location/object was used to calculate the discrimination index ([(T_novel_ − T_familiar_)/(T_novel_ + T_familiar_)] × 100). An experimenter blind to genotype conducted experiment and data analysis.

Further information about behavioral tests can be found in S1 Methods.

### Hippocampal slice preparation and electrophysiology

Hippocampi from 8–11 weeks old male mice (*Jacob/Nsmf* ko and wt littermates) were cut using a vibratome (LeicaVT1000S) into 350 μm thick slices. Hippocampal slices were incubated for 2h in carbogenated (95% O_2_, 5% CO_2_) artificial cerebrospinal fluid (ACSF, containing in mM: 110 NaCl, 2.5 KCl, 2.5 CaCl_2_·2H_2_O, 1.5 MgSO_4_·7H_2_O, 1.24 KH_2_PO_4_, 10 glucose, 27.4 NaHCO_3_, pH 7.3) at 31±1°C. fEPSPs were evoked by stimulation of CA1 Schaffer-collateral fibers with biphasic rectangular current pulses (200 μs/polarity, frequency 0.033Hz) in a range of 4-5V through ACSF filled glass capillary microelectrodes (3–5 MΩ). fESPSs were recorded using ACSF filled capillary microelectrodes and amplified by an Extracellular Amplifier (EXT-023, npi) and digitized at a sample frequency of 5 kHz by Digidata 1401plus AD/DA converter (CED). Stimulation strength was adjusted to 40%~50% of the maximum fEPSP-slope values. Late-Long-term potentiation (L-LTP) was induced by tetanization consisting of three 1s stimulus trains at 100 Hz with a 6 min inter-train interval. Paired-pulse facilitation was measured by different interpulse interval (ms). Input-output curves relating the fEPSP slopes with the afferent volleys were generated from field recordings in an interface chamber. In our experimental conditions, we could more precisely quantify the afferent volley in this configuration. The general procedure was similar to what was described above. Data are represented as mean ± SEM.

### Primary mouse hippocampal cell culture and stimulation

Dissociated hippocampal neurons were prepared from P0-P1 *Jacob/Nsmf* ko and wt mice. Neurons were plated on glass coverslips coated with poly-L-lysine (Sigma-Aldrich) at a density of 40,000 for immunocytochemistry or 60,000 cells per well for transfection, in DMEM medium (Gibco, Thermo Fisher Scientific) supplemented with 8% FBS, 1% penicillin/streptomycin. Following attachment, cells were kept in Neurobasal medium (Gibco) supplemented with 0,5 mM Glutamax, B27, and 1% penicillin/streptomycin (all from Gibco), at 37°C, 5% CO_2_ and 95% humidity. Cells were divided into three groups, chronic BDNF treatment (100 ng/ml, Tocris) (DIV2 and DIV6), acute BDNF treatment for 30 min or no treatment. All cells were fixed at DIV10 or DIV15 in PBS containing 4% paraformaldehyde for 10 min at room temperature.

### Primary rat hippocampal cell culture and stimulation

Hippocampal neurons were dissected from E18 Sprague Dawley rats as previously described [2]. Neurons were plated on 18 mm glass coverslips coated with poly-D-lysine (Sigma-Aldrich) at a density of 30,000 per well (12 well plates) in 1 ml of DMEM medium supplemented with 10% FBS, 0,5 mM Glutamine and 1% penicillin/streptomycin (Gibco). After 1 DIV the medium was exchanged to 1 ml of Neurobasal including B27 and 0,2 mM Glutamine (Gibco). Cultures were incubated at 37°C, 5% CO_2_ and 95% humidity. At DIV11 neurons were divided into 4 groups, untreated, treated for 30 min with BDNF (100 ng/ml, Tocris), AP5 (20 μM, Sigma-Aldrich) or both. After treatment cultures were fixed with 4% paraformaldehyde for 10 min.

### BDNF reporter assay

To dissect the role of BDNF in *Bdnf* gene transcription in hippocampal neurons from Jacob ko and wt animals we employed a *Bdnf*-promoter (exonI and exonII) driven GFP reporter system (*Bdnf I and II*-eGFP, [2, 61]). Dissociated wt and *Jacob/Nsmf* ko DIV 8 hippocampal neurons were co-transfected with plasmids expressing *Bdnf I and II*-eGFP and plasmids expressing mRFP under the actin promoter as a volume marker and transfection control using Lipofectamine 2000 (Invitrogen) and kept for 48 hr. Following transfection, cells were divided into two groups, treated with BDNF (100 ng/ml, Tocris) or non-treated. Activity of the promoter was estimated by GFP-fluorescence levels upon indicated treatments in neuronal somata by measuring the GFP pixel intensity in the same ROI of maximal projections of two focal planes.

### Quantitative real time-PCR

P10 control and *Jacob/Nsmf* ko mice were sacrificed and tissue from CA1 and CA3 regions of the hippocampus was collected in PCR clean tubes (Eppendorf), freshly frozen in liquid N_2_ and stored at -80°C. Total RNA was isolated (RNeasy plus mini kit, Qiagen). 50 nanograms of RNA were reverse transcribed using random nonamers (Sigma-Aldrich) according to the manufacturer’s instructions (Sensiscript, Qiagen). *Bdnf exon IV* and glyceraldehyde 3-phosphate dehydrogenase (*Gapdh*) mRNA (as a reference gene) were amplified using the iScript RT-PCR iQ SYBR Green Supermix (BIORAD) in a real-time quantitative PCR (qPCR) detection system (LC480, Roche) using the following primers: *Bdnf exon IV* forward 5’-GCAGCTGCCTTGATGTTTAC-3’ and reverse 5’-CCGTGGACGTTTACTTCTTTC-3’, and forward *Gapdh* 5’-TGCTGAGTATGTCGTGGAG-3’ and reverse 5’-GTCTTCTGGGTGGCAGTGAT-3’. Each sample reaction was run in duplicate and Ct values of the reference genes from the samples were subjected to Grubbs’ outlier test. The relative expression levels were analyzed using the 2-ΔΔCt-method with normalization relative to GAPDH. Data were expressed as mean ± SEM. Two-tailed unpaired Student’s t-test was performed for comparison between two groups.

### BDNF ELISA assay

P10 and P45 mice were sacrificed and bilateral hippocampi regions of CA1 and CA3, were dissected and frozen with liquid N_2_ followed by storage at -80°C. Tissue was defrosted, scaled and lysed with lysis buffer (100 mM PIPES pH = 7, 500 mM NaCl, 0.2% Triton X-100, 0.1% NaN_3_ 2% BSA and Complete protease inhibitor cocktail with EDTA, Roche). Hippocampi were sonicated and centrifuged at 16,000 g for 30 min at 4°C. 100 μl of supernatant was diluted in 4 volumes of DPBS buffer and acid treated with 10 μl of 1N HCl to decrease pH below 3.0. After 15 min samples were neutralized with 10 μl of 1N NaOH. The BDNF Elisa was performed with BDNF Emax ImmunoAssay System (Promega) according to manufacturer’s protocol. Briefly, 96-well plate was coated with 100 μl of anti-BDNF antibody (1:1000) over night at 4°C. Plates were blocked with 200 μl 1x Promega Block and Sample buffer for 1 hour at room temperature. Following blocking samples were incubated at room temperature together with standard dilutions for 2 hours with shaking (400 rpm). After washing with TBST, samples were incubated with 100 μl of anti human BDNF polyclonal antibody (1:500) for 2 hours at room temperature. Following washing, 100 μl of anti-IgY horseradish peroxidase conjugate was added and incubated for 1 hour at room temperature. Plates were emptied again, washed with TBST buffer and developed with 100 μl of TMB One Solution. Reaction was stopped with 100 μl of 1N HCl. Absorbance was measured at 450 nm. Data were expressed as mean ± SEM. Two-tailed unpaired Student’s t-test was performed.

Information about histological techniques, measurement of neuron number and fiber density, Golgi-Cox staining, TIMM staining, volumetric analysis of mouse brain by means of manganese-enhanced MRI, confocal laser-scanning and immunocytochemistry can be found in S1 Methods.

## Supporting Information

S1 Methods Supplemental Material and Methods. (PDF)

S1 Table Results of different behavioral parameters during the exposition of *Jacob/Nsmf* ko and wt mice to TMT or DEP. Data are presented as mean ± SEM, data analysis of individual mice from both genotypes was performed using multivariate analyses of variance (MANOVA’s) with GENOTYPE and ODOR as the between-subject factors.
(PDF)

S2 Table Social interaction test with *Jacob/Nsmf* ko and wt mice. Data are presented as mean ± SEM, using unpaired t-tests (Welch’s test); data collected from pairs of mice of each genotype were analyzed.
(PDF)

S1 Fig Generation of *Jacob/Nsmf* ko mice. (A) *Jacob/Nsmf* gene structure and targeting construct. Exons 1–3 were flanked by loxP sites to enable Cre-mediated deletion. (B) Genotyping strategy to differentiate between wild-type (wt), heterozygous (het) and homozygous (ko) mice with specific primers. (C) RT-PCR on wt (lane 1, 2) and *Jacob/Nsmf* ko mouse mRNA (lane 4, 5) shows that all known splice isoforms are absent in ko mice. (D) Western blots on brain tissue from different areas of wt, *Jacob/Nsmf* het and ko mice (Cx, Cortex, Hc, hippocampus, Bo, olfactory bulb, Hyp, hypothalamus, Str, striatum). (E) Litter size of homozygous breeders is comparable to heterozygous and wt breeding pairs (n = 4). The mendelian ratio of litters from heterozygous breeders is as expected. Body weights (age of 3 weeks and 4 months) of Jacob-deficient mice do not differ from wt mice.
(TIF)

S2 Fig Chromosome preparation of *Jacob/Nsmf* ko and wt mice testis stained with Giemsa. (A, E) The chromosome preparation shows the normal 40 acrocentric chromosomes (arrowhead 1, mitosis chromosomes during cell division, 2, immature sperm cell, 3, spermatogonia). (B, F) Arrowhead 4 shows condensation of chromosomes during Metaphasis I—Pachytene. (C, G) Arrowhead 5 shows separation of the homologous chromosomes after exchanges, 6 show chromosomal crossovers. A tetrad consists of the two homologous chromosomes with their four chromatides. 7 displays a Prophasis I cell. (D, H) Arrowhead 8 indicates Metaphasis II chromosomes. Scale bar, 5 μm.
(TIF)

S3 Fig *Jacob/Nsmf* ko mice do not show signs of anosmia or hyposmia. To test anosmia or hyposmia in *Jacob/Nsmf* ko mice and wt controls (n = 8, males) the animals were singly placed into the center of the box. Freezing behavior was analyzed for 15 min either to TMT or DEP that was pipetted on filter papers and administered through a side lid. Odor exposition experiments were analyzed using multivariate analyses of variance (MANOVA) with ODOR (two levels: TMT and DEP) and GENOTYPE (two levels: +/+ and -/-) as the between-subject factors. Data are presented as mean ± SEM.
(TIF)

S4 Fig General brain morphology of *Jacob/Nsmf* ko mice is normal. Microphotographs of Nissl-stained coronal sections displaying the brain morphology of wt (+/+) and *Jacob/Nsmf* ko (-/-) mice at this level (Bregma -1.94 mm). There are no differences in general morphology between both genotypes (scale bar 1 mm).
(TIF)

S5 Fig Volumetric analysis of various brain structures using manganese-enhanced MRI. (A) Comparison of the total brain volume and the volume of different brain structures of *Jacob/Nsmf* ko (-/-)and wt (+/+) littermates. Significant difference between the two groups (p<0.05) is indicated by an asterisk. (B) 3D surface rendering of the striatum in wt (+/+) and ko (-/-) mice.
(TIF)

S6 Fig *Jacob/Nsmf* ko mice display hyperactive behavior in the open field. (A) Motor behavior of Jacob-deficient mice and wt littermates (n = 12, each genotype, both sexes) was analyzed on the rotarod. (B, C) Spontaneous behavior was tested in the open field and revealed significant differences between wt and *Jacob/Nsmf* ko mice towards speed and distance covered (n = 12, each genotype, both sexes; two-way ANOVA, *p ≤ 0.05).
(TIF)

S7 Fig *Jacob/Nsmf* ko mice do not show impaired auditory cortex-dependent discrimination learning in the shuttle box compared to wt mice. Male mice (*Jacob/Nsmf* ko: n = 10, wt littermates: n = 8) were trained in a two-way shuttle box GO/NO-GO task to discriminate between sequences of rising (4–8 kHz, CS+) and falling (8–4 kHz, CS-) frequency modulated tones. Mice had to respond the presentation of CS+ by a hurdle crossing (hit), while they had to remain in the current compartment during 6s of CS- presentation (correct rejection). Errors (misses, false alarms) were punished by a mild foot-shock. Each of the 13 daily training sessions consisted of 60 trials with 30 randomized presentations of CS+ and CS-, respectively. (Two-way ANOVA, values are mean ± SEM).
(TIF)

S8 Fig *Jacob/Nsmf* ko mice have normal base line, paired pulse facilitation and basal synaptic transmission. (A) Baseline stability experiments revealed no difference between wt and *Jacob/Nsmf* ko mice. (B) Paired-pulse facilitation ratio (PPF ratio, P2/P1) was plotted against different inter-pulse intervals as second divided by first fEPSP. (C) Input-output curves showing the relationship between the fEPSP slope and the afferent volley in wild-type (wt) or Jacob/Nsmf knockout mice (ko). There was a trend to smaller fEPSP slopes in the knockout animals, but there were no statistically significant differences between the input-output curves. Field potential traces for both groups are shown as inset (grey traces: wt, black traces: ko). Scale bars 5mV/1ms. Data are represented as mean ± SEM.
(TIF)

S9 Fig *Jacob/Nsmf* ko mice display an enlargement of the dentate gyrus projection compared to wt mice. Representative TIMM-stained hippocampal area in wt (A) and *Jacob/Nsmf* ko mice (B). In *Jacob/Nsmf* ko mice the complete DG is significant enlarged compared to wt mice. (C-E) Morphometry in the hippocampus. (C) Significant differences could be detected in complete DG and DG + CA3 area. The TIMM-positive structures in hippocampus (D) and also the ratio of suprapyramidal mossy fibers (SPMF) and infra- and intrapyramidal mossy fibers (IIPMF) (E) did not show any differences. (ANOVA * p<0.05; scale bar in B = 500 μm). (F, G) Microphotographs of coronal sections showing the distribution of tyrosine hydroxylase (TH)-IR fibers in dorsal hippocampus of wt and *Jacob/Nsmf* ko mice. (H-J) A significant difference between genotypes was found in CA3 (I), but not in CA1 (H) and DG (J). Two way repeated measures ANOVAs were performed using LAYER (three levels in CA1 and CA3: Or, stratum oriens, Rad, stratum radiatum, LMol, stratum lacunosum moleculare; two levels in DG: Mol, stratum moleculare, ML, stratum multiforme) as within-subject factor and MOUSE LINE (two levels: wt and *Jacob/Nsmf* ko mice) as between-subject factor. Scale bar in G = 100 μm.
(TIF)

S10 Fig Distribution of tyrosine hydroxylase immunoreactivity fibers in the dorsal hippocampus of wt and *Jacob/Nsmf* ko mice. For overview and quantification see S8F–S8J Fig. From top down images from CA1, DG and CA3 layers (CA1 and CA3: Or, Rad, LMol; DG: Mol, ML) of each genotype are shown (scale bar 50 μm). The exemplary images are taken from the same mouse, respectively.
(TIF)

S11 Fig Distribution of tyrosine hydroxylase immunoreactivity in the striatal complex and ventral tegmental area (VTA) of wt (+/+) and *Jacob/Nsmf* ko (-/-) mice. (A, B) In the striatum (Bregma 1.18 mm) the dotted lines indicate the boundaries of the core (AcbCore) and shell (AcbShell) regions of the nucleus accumbens. Square sample fields (200 x 200 μm) were used for grey scale analysis. Scale bar 200 μm. (E) No significant effect of MOUSE LINE was shown for the TH-IR fiber densities in nucleus accumbens. Also, no differences were found in the caudate putamen (CPu) (n.s.). Data are reported as mean±SEM. For analysis of the nucleus accumbens a two way repeated measures ANOVA was performed using REGION (two levels: AcbCore, AcbShell) as within-subject factor and MOUSE LINE (two levels: +/+ and -/- mice) as between-subject factor. Post hoc analyses were performed using unpaired t-tests with Bonferroni-Holm adjustment. The CPu was analyzed using unpaired t-tests (Welch’s tests). * p<0.05. (C, D) Distribution of tyrosine hydroxylase immunoreactivity in the ventral mesencephalon of wt (+/+) and ko (-/-) mice. The dopaminergic areas comprise the ventral tegmental area (VTA) and the substantia nigra subregions pars compacta (SNC), pars reticulata (SNR) and pars lateralis (SNL) (Bregma -3.28 mm, scale bar 200 μm). (F) Neuron numbers of TH-IR neurons (n), volume (mm^3^) and neuron densities (n/mm^3^) of the VTA. No significant differences were found. Statistics: data are reported as mean±SEM. Analyses were performed using unpaired t-tests (Welch’s test).
(TIF)

S12 Fig *Jacob/Nsmf* ko mouse cortical primary neurons show no alternations in dendritic tree complexity and synapse number. (A) Representative micrographs of wt and *Jacob/Nsmf* ko cortical neurons immuno-stained with MAP2 at DIV10. For sholl analysis the number of dendritic intersections of wt and *Jacob/Nsmf* ko cortical neurons was plotted against the distance. (B) *Jacob/Nsmf* ko neurons do not display a reduced number of synaptic contacts as compared to wt controls. Representative micrographs of DIV15 wt and *Jacob/Nsmf* ko cortical neurons primary distal dendrites immuno-stained with MAP2, Homer1 and Synaptophysin. Co-localization of synaptic puncta per 10 μm was quantified. A Student´s t-test did not show significant differences. Scale bar in (A) = 50 μm, panels in (B) = 20 μm.
(TIF)

S13 Fig Differences in expression of GFP under control of the *Bdnf* I+II promoter in *Jacob/Nsmf* ko and wt hippocampal neurons. (A) DIV10 hippocampal neurons were transfected with construct overexpressing GFP under *Bdnf* I+II promoter. After 24h of expression and treatment with BDNF, cultures were fixed and confocal images were acquired. (B) In *Jacob/Nsmf* ko neurons as well as wt there was significant increase after of GFP intensity after BDNF treatment, however in case of ko neurons the increase in GFP intensity was lower. Two-way ANOVA with Bonferroni posttest, ***p<0.001; **p<0.01 (B, left side) and two-tailed unpaired t-test (B, right side) ***p<0.001. Scale bar is 20 μm.
(TIF)

S14 Fig Simplification of Jacob-deficient mouse neurons can be rescued by BDNF but not by NGF or boiled BDNF. *Jacob/Nsmf* ko mouse hippocampal neurons and wt neurons were treated with BDNF, boiled BDNF or NGF (all 100 ng/ml) at DIV2 and DIV6, fixed at DIV10 and stained for MAP2. (A) Graphs representing the number of dendritic intersections of wt and *jacob/nsmf* ko hippocampal neurons plotted against the distance. At DIV10 *Jacob/Nsmf* ko hippocampal neurons display a simplified dendritic tree as compared to wt neurons. (B) *Jacob/Nsmf* ko neurons display a reduced number of synaptic contacts as compared to wt controls. Representative micrographs of DIV15 wt and *Jacob/Nsmf* ko hippocampal neurons primary distal dendrites immuno-stained with MAP2, Homer1 and Synaptophysin, untreated, treated with BDNF, with boiled BDNF or NGF. Co-localizing synaptic puncta were quantified per 10 μm. Statistical differences were analysed with two-way ANOVA and Post-hoc Bonferroni tests (* p<0.05, **p<0.01, ***p<0.001). Panels in B = 20 μm.
(TIF)

S15 Fig TrkB and pTrkB levels are unchanged in the hippocampus of *Jacob/Nsmf* ko and wt mice. Immunoblots of hippocampal lysates from 6 wt and 6 *Jacob/Nsmf* ko animals: (A) Anti Trk-B antibody detects both full-length (FL) and truncated (Trunc) forms of TrkB. (B) Anti phosphoTrk antibody. The quantification of levels of TrkB (C), pTrk (D) normalized to actin did not reveal statistically significant changes.
(TIF)

S16 Fig LTP cannot be rescued following BDNF application in slices of *Jacob/Nsmf* ko mice. Application of 100ng/ml BDNF during tetanus has no effect on LTP in ko mice. The arrows indicate high frequency stimulus (HFS) lasting 1s at 100Hz, 3x HFS. The horizontal bar indicates the period during BDNF was added into the bath solution. Data are represented as mean ± SEM.
(TIF)

S17 Fig Primary hippocampal neurons do not show extracellular Jacob localization. (A) Primary neurons (DIV7) express Jacob intracellularly (permeabilized), but not extracellularly (B) (Jacob live) as there is no higher staining than in secondary antibody controls (C). MAP2 staining was included to outline dendrites. (D) Live-staining of Prion protein (DIV7 and DIV21) proves reliability of the live-staining protocol. Scale bar is 20μm.
(TIF)

S18 Fig Characterization of the pan-Jacob antibody employed. (A) pan-Jacob antibodies were generated against rat N-terminal peptide aa 187–203 corresponding to rat Jacob sequence which is slightly different from the mouse Jacob sequence. Antibodies do recognize heterologously expressed rat WT-Jacob-GFP, but not the mouse WT-Jacob-GFP protein. (B) Antibodies recognize only the part of Jacob where the amino acid sequence used for rabbit immunization is represented. (C) Plasmid based shRNA knockdown dramatically reduced endogenous Jacob levels in mature hippocampal neurons. Scale bar is 40μm.
(TIF)

S19 Fig Acute BDNF administration has no effect CREB immunofluorescence levels in mouse hippocampal primary neurons. (A, B) Following bath application of BDNF (100ng/ml) at DIV10 the total nuclear CREB levels are unchanged in *Jacob/Nsmf* ko and wt hippocampal neurons. Scale bar is 20μm.
(TIF)
