# Supplementary material for: A Jacob/Nsmf Gene Knockout Results in Hippocampal Dysplasia and Impaired BDNF Signaling in Dendritogenesis
Source: PLoS Genet. 2016 Mar 15;12(3):e1005907. doi: 10.1371/journal.pgen.1005907 (PMC4792503; doi:10.1371/journal.pgen.1005907)
Supplement: S1 Methods — (PDF) [file pgen.1005907.s001.pdf]

Supporting Information

Supplemental Material and Methods

Genotyping. Genotypes were confirmed by standard PCR protocols using the following primers: F-Jac_5574 CTGAGGCTGAGACCTAGCGC; R-Jac_6032 CAGCCTCCAATACTGGCAAGAC; R-Jac_7329 GGAAGGGACTTCATCCTGACTG resulting in bands of 459 bp for wt and 187 bp for ko condition. PCRs were performed on tail biopsy after lysis in tail-cut buffer (10 mM Tris HCl pH 8.0, 100 mM NaCl containing Proteinase K at a final concentration of 0,4 mg/ml).

RT-PCR. RNA was extracted from wt and *Jacob/Nsmf* ko mouse hippocampus using a rotating tissue homogenizer and the Oligotex Direct mRNA Kit (Qiagen) following the manufacturer´s instructions. Reverse transcription was performed with Omniscript (Qiagen) using Oligo(dT) primers for 2 h at 37°C. Jacob cDNA was amplified in two PCRs using nested primers in the second. In detail the following primers were used: 1. PCR, 5´ex1_223 ATGGGTGCCGCCGCCTCCAG and 3´ex16_1798 CAGGACGTCATCAAAGTCCAGCAG (30 cycles; 95°C for 30 sec; 62°C for 30 sec, 72°C for 1 min); 2. PCR, using as template 1 µl of first PCR reaction, 5´ex2_295 GCAGCCCGAGCGTTTGGAGAG or 5ex6_950 GCTACGCGGAGAGGAAGCGTC and a common reverse primer 3ex12_1442 CTGGCAGAAAATCAGCATCTTCC (30 cycles; 95°C for 30 sec; 60°C for 30 sec, 72°C for 1 min). As control primers specific for mouse GAPDH were used (5´ ACCACAGTCCATGCCATCAC; 3´ TCCACCACCCTGTTGCTGTA).

Preparation of mouse brain tissue lysates and immunoblotting. Freshly dissected tissue from adult *Jacob/Nsmf* ko and wt mice was shortly rinsed in PBS and then snap frozen in liquid nitrogen and stored at -80°C. Homogenization was done in buffer containing 10 mM Tris/HCl pH 7.5, 0,5% TritonX-100 and Complete protease inhibitor cocktail (Roche

Diagnostics) using a hand homogenizer in a ratio of 10ml/g wet weight. Tissue homogenates were then mixed with 4x SDS sample buffer (250 mM Tris pH 8.5, 8% SDS, 40% glycerol, 2 mM EDTA, 0.01% bromphenol blue, 100 mM DTT) in a ratio of 2:1, boiled for 10 min and centrifuged at 17000 x g for 5 min. Total protein concentration of supernatants was determined by Amidoblack protein assay and same amounts of tissue extracts (20 µg per lane) were loaded for SDS-PAGE and blotted onto nitrocellulose (Schleicher and Schuell).

To compare TrkB and phospho Trk protein content in wt and ko mice, hippocampi were dissected from p10 animals, snap frozen in liquid nitrogen and homogenized in Tris buffer containing phosphatase inhibitor (PhosSTOP, Roche) and protease inhibitors (Complete tablets Mini, EDTA-free, Roche). Samples were run on 4-12% gradient gels and immunoblotted. After blocking in 5% milk powder in Tris-buffered saline / Tween (TBS-T, 20 mM Tris, 150 mM NaCl, pH 7.4, 5% Tween-20) or 5% BSA in TBS-T membranes were incubated with primary antibodies diluted in TBS-T.

The following primary antibodies were used for immunodetection: A guinea pig anti-panJacob antibody raised against a GST fusion protein (GST-Jacob-253–404), a rabbit anti-panJacob peptide antibody raised against the rat peptide sequence MKADTSHDSRDSSDLQ (aa 299-314) [2], mouse anti-Actin (Sigma-Aldrich), rabbit anti-TrkB (Millipore) or rabbit anti-pTrkA (Tyr490, Cell Signaling). Immunoreactivity was visualized using HRP-coupled goat anti-rabbit (GE Healthcare or Dianova), goat anti-mouse or goat anti-guinea pig secondary antibodies (Dako) and the ECL detection system (Thermo Fisher Scientific). Images were acquired with Fujifilm LAS-300 system (Thermo Fisher Scientific). Blots were quantified using Fiji software.

Chromosome preparation. The preparation followed the method described by Schleiermacher [62], which allows to evaluate meiotic as well as mitotic cells at the same slide. Male mice were deeply anesthetised with sodium pentobarbital (180 mg/kg i.p.; Merial GmbH) to remove the testes. The dissected parenchymal tissue of the testes was stored in 2.2% sodium citrate solution and chopped to small pieces. Afterwards, the tissue was

immersed for 20 min in a solution of sodium citrate (1.1 %) and hyaluronidase (0.005%, Sigma-Aldrich) at 37°C in order to obtain a single-cell suspension, followed by centrifugation at 1200 x g for 10 min. After fixation for 10 min in a methanol / glacial acetic acid solution (3:1 ratio) and subsequent centrifugation at 1200 x g for 10 min the supernatant was removed. Samples were re-suspended in the same fixative and let to rest for one hour at 4°C. Cells were then spread on uncoated glass slides, air dried and stained with the Giemsa method. Microscopic analysis was performed using a Zeiss Axioplan2-microscope (Carl Zeiss Light Microscopy) equipped with an AxioCam HRc microscope camera (Carl Zeiss MicroImaging GmbH) and 100x oil immersion objective. Spermatogonia were checked for chromatid-type and the following chromosome-type aberrations: gaps, breaks, chromatid exchanges, fragments, atypical monocentric and dicentric chromosomes.

Behavioral tests

Rotarod. Animals received two training sessions (3 h interval) on a rota-rod apparatus (TSE) with increasing speed from 4 to 40 rpm for 5 min. After 4 days, mice were tested at 16, 24, 32, and 40 rpm constant speed for 5 min. maximum duration as described [63]. The latency to fall off the rod was measured. Analysis of Variance (ANOVA, factors genotype, sex), repeated measures ANOVA (genotype, session) and post hoc analysis (Scheffe's or Fisher PLSD) were conducted using Statview software (SAS Institute Inc., Cary, NC). p-values smaller than 0.05 ($p<0.05$) were considered significant.

Auditory discrimination learning (shuttle box). Male mice (*Jacob/Nsmf* ko: n=10, wt littermates: n=8, age 4-5 month) were trained in a two-way shuttle box GO/NO-GO task to discriminate between sequences of rising (4–8 kHz, CS+) and falling (8-4 kHz, CS-) frequency modulated tones to avoid mild foot-shock, as described earlier [64]. A training session consisted of 60 trials, i.e. 30 presentations of each CS+ and CS− in pseudo-randomized order, and lasted ≈25 min. The mean (± SEM) inter-trial interval lasted 20 ± 4 s.

Open Field. Exploration was assessed by placing mice in the middle of a 50 x 50 cm arena (for 15 min) [63]. Using the VideoMot 2 system (TSE), tracks were analysed for path length, visits, walking speed, and relative time spent in the central area (infield, 30 x 30 cm), in the area close to the walls (<10 cm, outfield), and in the corners (10 x 10 cm). Statview (SAS Institute Inc.) software was used for Analysis of Variance (ANOVA, factors genotype, sex) and post hoc analysis (Scheffe's or Fisher PLSD). p-values smaller than 0.05 ($p<0.05$) were considered significant.

Odor exposure. 2,5-Dihydro-2,4,5 trimethylthiazoline (TMT, PheroTech), a synthetic compound originally isolated from fox faeces was used as an ethologically relevant olfactory stressor and diethylphthalate (DEP; Sigma-Aldrich) served as an odorless control substance. Odor exposure experiments took place in a light gray small-enclosed PVC box (l/w/h: 30 × 30 × 30 cm) with a sheer roof made out of acrylic glass (Plexiglas) in order to allow recording of behavior by a camera installed above the box. One day before testing (= habituation), mice were familiarized with the odor exposure environment in a single 15 min session and then divided into treatment groups. On the following day (= exposition) 14 mice (*Jacob/Nsmf* ko: n = 7, wt: n = 7) were exposed to TMT and 16 mice (*Jacob/Nsmf* ko: n=8,wt: n=8) to DEP. Animals were singly placed into the centre of the box and then exposed for 15 min either to TMT (35µl) or DEP (35µl), which was pipetted on filter papers and administered through a side lid. To avoid odor interferences after the end of each experiment the odor exposure chamber was carefully cleaned with Incidin Foam (Ecolab GmbH & Co. OHG). All odor experiments were conducted under dim light conditions and recorded on videotapes. Animal behavior was evaluated by self-compounded software (created by Dipl.-Ing. C. Kurtz). Odor exposition experiments were analyzed using multivariate analyses of variance (MANOVA) with ODOR (two levels: TMT and DEP) and GENOTYPE (two levels: *Jacob/Nsmf* ko and wt) as the between-subject factors.

Social interaction test. Mice were tested in a light gray upwardly open PVC box (l/w/h: 85 × 85 × 30 cm) with homogenous, shadow-free neon-illumination (300 lx) to generate the highest level of anxiety. Each trail was recorded with a ceiling mounted camera (Panasonic CCTV Camera; Mod. WVBL200/6, Panasonic SD430 video recorder) and analyzed using the VideoMot2 video tracking software (TSE Systems) on a standard computer. At the commencement of each trial (duration of 10 min), two mice with the same genotype were placed into opposite corners of the open field, recorded and automatically analyzed by the software for various parameters (S2 Table). Measurement of distance travelled was carried out as a sign of motor activity, anxiety-like behavior as time spent in the rim or center region. Afterwards, the coded video tracks were manually analyzed for a second set of parameters (S2 Table). The data of the individual pairs were used for statistics. With self-compounded software, using fuzzy logic, the collected raw data were additionally processed to compensate weaknesses of the video tracking software [65]. The social interaction test was analyzed using unpaired t-tests for unequal variances (Welch's test). Because of the dissimilarity of parameters, no alpha-adjustment was carried out.

Histological techniques

Brain preparation for immunohistochemical analyses. Mice were anesthetized with sodium pentobarbital (180 mg/kg i.p.; Merial GmbH), perfused intracardially with 50 ml of saline followed by 100 ml of fixative (4% paraformaldehyde and 15% saturated picric acid in phosphate buffer; 0.1 M, pH 7.4) and then decapitated. Brains were rapidly removed from the skull and post-fixed overnight in same fixative, followed by 24 h storage in 20% sucrose at 8°C. Subsequently, brains were frozen in carbon dioxide snow cooled liquid isopentane at −40°C and stored at −80°C. Brains (*Jacob/Nsmf* ko: n=8, wt: n=8) were cut coronally with a cryostat into 40 µm thick sections (five comparable series from one brain) beginning at the olfactory bulb. Two of the series were stained for: Cresyl violet (Nissl), and GnRH to analyze neuron number and fiber density in *Jacob/Nsmf* ko and wt mice.

To label GnRH-IR and TH-IR neurons and fibers, free floating sections were incubated with polyclonal rabbit anti-GnRH (dilution 1:2000; ab5617; Abcam) or rabbit TH polyclonal antibody (dilution 1:1000; ab112; Abcam). Incubations with primary antisera were carried out using phosphate buffer (0.1 M, pH 7.4) with 0.3% Triton X-100 (Sigma-Aldrich) to improve penetration of the antibodies. Unspecific bindings of polyclonal antibodies were blocked before incubation using 10% bovine serum albumin (BSA) in phosphate buffer (0.1 M, pH 7.4) and 10% normal goat serum (NGS) as the corresponding normal serum from the host of the secondary antibody. Secondary antibodies (biotinylated anti-rabbit IgG for GnRH and TH; Vector Laboratories) were used in a dilution of 1:200. Antibodies were visualized by incubation with Cy3 conjugated avidin (dilution 1:1000, Jackson ImmunoResearch Laboratories). To minimize experimental variability, all sections to be compared were stained in parallel, using the same solutions. After staining, sections were mounted on glass slides (Super Frost Plus, ThermoScientific) and coverslipped.

Measurement of neuron number and fibre density. Measurement of neuron number and fiber density was carried out manually and in a blinded manner. For microscopy and photographs a Zeiss AxioImager.Z2 fluorescence microscope equipped with an AxioCam MRm Rev. 3.1 camera (Carl Zeiss MicroImaging GmbH) and a 40x objective was used. The Axiovision 4.8.1 Software (Carl Zeiss Microscopy GmbH) served for taking scaled microphotographs (exposure time 250 ms). The Nissl stained series was used to identify anatomical landmarks [66]. The number of GnRH-IR neurons was estimated in all sections containing the areas of interest in one series of *Jacob/Nsmf* ko (n = 8) and wt (n = 8) mice. GnRH-labeled neurons were mainly found in stratum glomerulosum of olfactory bulb (Gl, starting at bregma 4.28 mm), stratum plexiforme externum of olfactory bulb (EPl, starting at bregma 4.28 mm), medial septum (MS, starting at bregma 1.18 mm), ventral diagonal band of Broca (VDB, starting at bregma 1.42 mm), horizontal diagonal band of Broca (HDB, 0.86 mm) and preoptic area (PA, 0.38 mm). The analysis of TH-IR neuronal number, volume and neuron density in ventral tegmental area (VTA) of the right hemisphere was carried out from

bregma -2.46 to -3.52 mm in a second series of *Jacob/Nsmf* ko (n = 6) and wt (n = 6) mice. All sections containing the area of interest were used. Only cell bodies with clear neuronal shape and well-defined nucleus were counted. Additionally, the neuron diameters were estimated and used for Abercrombie correction of neuron number [67].

Measurement of GnRH-IR fiber density was performed in stratum glomerulosum of olfactory bulb (Gl, starting at bregma 3.20 mm), stratum plexiforme externum of olfactory bulb (EPl, starting at bregma 3.20 mm), medial septum (MS, starting at bregma 1.18 mm), ventral diagonal band of Broca (VDB, starting at bregma 1.18 mm), horizontal diagonal band of Broca (HDB, starting at bregma 0.62 mm), anteroventral paraventricular hypothalamic nucleus (AVPe, starting at bregma 0.38 mm), nucleus arcuatus hypothalami (Arc, starting at bregma -1.22 mm) and preoptic area (PA, starting at bregma 0.02 mm). From five consecutive slices one image of each subregion was taken and analyzed, respectively. The density of TH-IR fibers in the dorsal hippocampal formation was investigated in the right hemisphere. The CA1 and CA3 were divided into stratum oriens (Or), stratum radiatum (Rad) and stratum lacunosum moleculare (LMol) and the DG into stratum moleculare (Mol) and stratum multiforme (ML). The measurements started at bregma -1.7 mm. From five consecutive sections of each subregion one image of each layer was taken. For analysis of microphotographs the software ImageJ (version 1.46m, http://rsb.info.nih.gov/ij/) was used. According to Russ and Dehoff [68] the fiber densities were estimated with a calibrated grid (mesh size of 10 μm) and a square (length of one side (L) was 80 μm). The number of planes (P = 9) that correspond to the vertical and horizontal lines of the grid and the margin of the square, were represented in two sets of planes (2×P). The area (A) was calculated by $A = L \times 2 \times P = 1440\ \mu m^2$. All intersections (n) of fibers with the grid within the square were counted. There is a relationship between the number of points per unit area (PA) and the specific line length per unit volume (LV): $PA = 1/2 \times LV$. If $LV = 2 \times PA$ ($\mu m/\mu m^3$) and $PA = n/A$ (counts/μm^2) the estimated length within a volume (fiber density) was gained.

For TH-staining of the striatum the right striatal complex of *Jacob/Nsmf* ko (n = 6) and wt (n = 6) mice was divided into caudate putamen (CPu), nucleus accumbens core (AcbCore) and

shell (AcbShell). Because of dense TH-IR innervation of the striatum a grey scale analysis was used. The measurements started at bregma 1.7 mm. In three consecutive slices images of each subfield were taken (5x objective). The mean grey values in the regions of interest (S3 Fig) were calculated with ImageJ using square sample fields (200 x 200 µm). The mean grey values were normalized to the grey value of the unstained anterior commissure (ac), respectively. Concerning of the patchy distribution of fibers caused by the striosome/matrix compartmental organization of the striatum, sample fields had to be carefully selected to match exact regions.

Data are presented as means ± SEM. Data were normally distributed (Shapiro Wilk test). The Levene test was performed to prove homogeneity of variance. If the sphericity assumption for ANOVA was rejected, the Greenhouse-Geisser correction was used. Differences in neuron number and fiber density of *Jacob/Nsmf* ko and wt mice were analyzed by repeated-measures ANOVAs with REGION (GnRH-IR neurons: six levels, GnRH-IR fibers: eight levels, TH-IR fibers: three or four levels, depending on the number of subregions or layers investigated) as the within-subject factor and GENOTYPE (two levels: *Jacob/Nsmf* ko and wt) as the between-subject factor. Post hoc analyses were performed using unpaired t-tests for unequal variances (Welch's test) and adjusted p-values were obtained *via* the Bonferroni-Holm method. TH-IR neuronal numbers, volume and neuron density in VTA (Fig. 7) and TH-IR of the CPu (S3 Fig) were analysed using unpaired t-tests (Welch's test). The alpha level was set at 0.05 for all main and interaction effects. The software package IBM® SPSS® Statistics Version 21 was used for statistical analysis.

Golgi-Cox staining. Histological procedures for the Golgi-impregnation of the mouse brains largely correspond to those described by Mylius and coworkers [69]. 6 mice of each genotype (age 12 weeks) were deeply anaesthetized with an overdose of pentobarbital (Sigma-Aldrich), exsanguinated and brains were removed and impregnated in the dark for fourteen days at room temperature in 50 ml of a Golgi-Cox solution [70]. After impregnation, brains were dehydrated in a graded series of ethanol, treated in a mixture of 100% ethanol

and anhydrous diethylether for four hours and embedded in a graded series of celloidin. Within the next days, brain-containing celloidin blocks were formed and dried in a desiccator under exposure to phosphorus pentoxide (Merck) and polymerized and hardened under exposure to chloroform (Merck). Afterwards, brains were cut on a sliding microtome (Microm) into serial horizontal sections of 150 µm thickness. Sections were collected in 70% ethanol and rinsed in destilled water. Then, they were treated in a 50% alkaline ammonia solution and then in 0.5% phenylen-diamine (Sigma-Aldrich). The staining was developed in 1% dectol (Kodak) and fixed in 5% tetenal (Calbe Fotochemie). Finally, sections were rapidly dehydrated in a graded series of ethanol and xylol (Roth) and mounted with Merckoglas (Merck).

Neuron selection criteria and spine density assay. The brain morphology of wt and *Jacob/Nsmf* ko mice was inspected and analyzed using a light microscope system (Leica DMRX) with motorised stage (Märzhäuser). Representative pyramidal neurons (2 to 5 neurons from at least 4 animals) from hippocampal CA1 region of the left hemisphere were reconstructed 3-dimenionally by Neurolucida software (Microbrightfield Europe). For the quantitative analysis of their branching patterns and spine density the Neuroexplorer software (Microbrightfield) and the Excel spreadsheet application (Microsoft) was used.
For statistical analysis GraphPad Prism 6 software (GraphPad Software, Inc) was used (two-way ANOVA or Student's t-test, depending on the experiment). A probability level of $P \leq 0.05$ was considered as statistically significant.

Zinc autometallography (Timm staining). Animals (*Jacob/Nsmf* ko: n=4; wt: n=5) were deeply anaesthetized (20 mg ketamine and 1.0 mg xylazine/100 g body weight, i.p.) and perfused transcardially with 200 ml of 0.1% sodium sulfide in 0.1 M phosphate buffer (PB, pH 7.4) followed by 200 ml of 4% paraformaldehyde (PFA) in 0.1M PB. Brains were removed, post-fixed overnight in 4% PFA, and then cryoprotected by placing them in 30% sucrose in PB for 48 hours at 4°C. After freezing brains at -50°C, 25 µm thick transversal

sections were made. Sections were mounted on gelatine-coated glass slides, dried at 30°C for 24 hours and then stained for Zn^{2+} by a modified Timm-staining as described by Danscher [70]. Slices were developed for approximately 150 minutes in a gum arabicum solution containing citric acid buffer (pH 3.8), hydroquinone, and silver nitrate. Development was stopped by washing the sections under running tap water for 10 minutes. After drying them overnight they were coverslipped by Entellan (Merck).

Volumetric analysis of mouse brain by means of manganese-enhanced MRI

Mice were anesthetised with 1.0-1.5% isoflurane (in 50:50 N_2:O_2, v:v) and fixed using a head-holder with bite bars to reduce motion artifacts. MRI experiments were performed on a Bruker Biospec 47/20 scanner at 4.7T (free bore of 20 cm) equipped with a BGA 09 (400 mT/m) gradient system. A 25-mm Litzcage small animal imaging system (DotyScientific Inc.) was used for RF excitation and signal reception. Two days before MRI measurement, animals were injected subcutaneously with an aqueous solution containing 1 µmol/g $MnCl_2$ (e.g. 200 µl of a 100 mM $MnCl_2$ solution for a 20 g mouse, which is equivalent to 125 mg/kg). A data set of T_1-weighted images was obtained using a 3D MDEFT (modified driven equilibrium Fourier transform) pulse sequence with the following parameters: TR 13.6 ms, TE 4.3 ms, flip angle 20º, FOV 30x30 mm, matrix 256x256 (yielding a nominal in plane resolution of 117 x 117 µm), slice thickness 300 µm, 24 averages; the total scanning time was 67 min.

All recognizable structures were manually segmented and the area measured using the public domain Java-based image processing and analysis program ImageJ (http://rsb.info.nih.gov/ij/). The volumes of individual brain structures were calculated by multiplying the areas with slice thickness of 300 µm.

Confocal laser scanning microscopy and quantitative immunocytochemistry

Following fixation, both murine and rat neurons were washed in PBS and blocked for 1 hour with blocking buffer (2% glycine, 2% BSA, 0.2% gelatine and 50 mM NH_4Cl). Next, neurons

were incubated overnight with the dilution of the following antibodies: rb anti-pCREB-Ser133 (1:500), rb anti-CREB (1:500, both from Cell Signaling), ms anti-pERK1/2 (1:500, Sigma-Aldrich), rb anti ERK1/2 (1:300, Enzo Life Sciences), rb anti-pJacob-Ser180 (1:300), rb anti-panJacob (1:300,), gp anti-synaptophysin (1:300 SySy) and ms anti-Homer1 (SySy). Affinity-purified phosphospecific Jacob antibody directed against phosphopeptide LVPGPSpPRAFG were characterized previously [2]. Characterization of affinity-purified panJacob antibodies raised against rat N-terminal Jacob peptide (aa187-203) EQPPLPEASGRHKKLER (custom service of Thermo Scientific) is provided in S18 Fig.

Following incubation with primary antibody cells were washed with PBS and incubated with secondary antibody anti-rabbit conjugated with Alexa Fluor 568 (Life Technologies) or anti-mouse conjugated with Alexa Fluor 647 (Life Technologies). After 2 hours incubation cells were washed and incubated for 1 hour with antibodies detecting MAP2 conjugated with Alexa Fluor 488 (1:1000, Life Technologies). After washing with PBS, cells were counterstained with DAPI (Sigma-Aldrich) and mounted. Images were acquired with Leica TCS SP5 (Leica) along Z-axis with 0.42 µm Z resolution. Scans were performed sequentially for every channel. For image quantification the FiJi software [72] was used. Two nuclear sections were merged and staining intensity was quantified within ROI defined by the DAPI channel.

Synapses were analyzed using FiJi software by identification of co-localizing puncta representing postsynaptic marker Homer1 (rb) and Synaptophysin (gp) by merging the channels of the images acquired and counted for 10 µm length of primary distal dendrites of both wt and *Jacob/Nsmf* ko DIV 10 neurons. Images of MAP2-positive neurons were acquired with Axio Imager.A2 fluorescent microscope (Zeiss). Concentric circles plugin based on FiJi software was used to measure the dendritic complexity.

Live-staining of rat hippocampal primary neurons

Live-staining of cells without preceding fixation and permeabilization was performed as follows. Hippocampal neurons were prepared and cultured as described in the manuscript at

a density of 20,000 per well (12 well plates) in 1 ml of Neurobasal including B27 and 0,2 mM Glutamine (NB+, Gibco). At DIV 7 and 21, respectively, primary antibodies were directly given into the culturing medium at dilutions of 1:250 (ms anti-Prion protein, Covance) and 1:200 (anti-panJacob, [16]), respectively, and neurons were incubated for 1h at 37°C, 5% CO_2 and 95% humidity. Afterwards, cells were washed 3 times with pre-warmed NB+ medium and fixed with 4% PFA. Anti-MAP2 antibody was added after permeabilization and blocking (see above). Secondary antibodies were applied for all IgG species and counterstaining was done with DAPI. Secondary antibody control was performed without addition of primary antibodies.

Supplemental references

62 Schleiermacher E. On the effects of trenimon and endoxan on meiosis in male mice. I. Method of preparation and analysis of meiotic divisions. Humangenetik 1966; 3: 127-133.

63 Montag-Sallaz M, Schachner M, Montag D. Misguided axonal projections, NCAM180 mRNA upregulation, and altered behavior in mice deficient for the Close Homolog of L1 (CHL1). Mol Cell Biol 2002; 22: 7967-7981.

64 Wolf R. Pharmakologische Beeinflussbarkeit von Verhaltensparametern in genetischen und läsionsinduzierten Tiermodellen der Schizophrenie. In: Hippius H, editor. Universitätskolloquien zur Schizophrenie, Band 2. Steinkopff Verlag, Darmstadt, 2004. pp. 460-467.

65 Ohl FW, Wetzel W, Wagner T, Rech A, Scheich H. Bilateral ablation of auditory cortex in Mongolian gerbil affects discrimination of frequency modulated tones but not of pure tones. Learn Mem 1999; 6: 347-362.

66 Paxinos G., Franklin KBJ. The Mouse Brain in Stereotaxic Coordinates: Compact Second Edition. Elsevier Academic Press, San Diego, CA. 2004.

67 Abercrombie, M. Estimation of nuclear population from microtome sections. Anat Rec 1946; 94: 239-247.

68 Russ JC, Dehoff RT. Classical stereological measures: Three Dimensional Microstructures: Line Length and the Area Point Count. In: Russ JC, Dehoff RT, editors. Practical Stereology. Plenum Press, New York, 1999. pp. 63-70.

69 Mylius J, Brosch M, Scheich H, Budinger E. The subcortical auditory structures in the Mongolian gerbil: I. Golgi-architecture. J Comp Neurol 2013; 521: 1289-1321.

70 Glaser EM, Van der Loos H. Analysis of thick brain sections by obverse-reverse computer microscopy: application of a new, high clarity Golgi-Nissl stain. J Neurosci Methods 1981; 4: 117-125.

71 Danscher G. Histochemical demonstration of heavy metals. A revised version of the sulphide silber method suitable for both light and electronmicroscopy. Histochemistry 1981; 71: 1-16.

72 Schindelin J, Arganda-Carreras I, Frise E, Kaynig V, Longair M, Pietzsch T, et al. Fiji: an open-source platform for biological-image analysis. Nat Methods 2012; 9: 676-682.
