# Supplementary material for: A Jacob/Nsmf Gene Knockout Results in Hippocampal Dysplasia and Impaired BDNF Signaling in Dendritogenesis
Source: PLoS Genet. 2016 Mar 15;12(3):e1005907. doi: 10.1371/journal.pgen.1005907 (PMC4792503; doi:10.1371/journal.pgen.1005907)
Supplement: S1 Table — Data are presented as mean ± SEM, data analysis of individual mice from both genotypes was performed using multivariate analyses of variance (MANOVA’s) with GENOTYPE and ODOR as the between-subject factors. (PDF) [file pgen.1005907.s002.pdf]

S1 Table. Results of different behavioral parameters during the exposition of *Jacob/Nsmf* ko and and wt mice to TMT or DEP. Data are presented as mean ± SEM, data analysis of individual mice from both genotypes was performed using multivariate analyses of variance (MANOVA's) with GENOTYPE and ODOR as the between-subject factors.

Parameter	***GENOTYPE x ODOUR***	***GENOTYPE***	***ODOUR***	***Up in***
Freezing [sec]	F(1,26)= 2.13, n.s.	F(1,26)= 2.59, n.s.	F(1,26)= 150.10, p<0.001	TMT
Sniffing [n]	F(1,26)= 0.08, n.s.	F(1,26)= 1.26, n.s.	F(1,26)= 1.68, p<0.001	DEP
Rearing [n]	F(1,26)= 0.13, n.s.	F(1,26)= 0.03, n.s.	F(1,26)= 23.40, p<0.001	DEP
Leaning [n]	F(1,26)= 0.27, n.s.	F(1,26)= 0.53, n.s.	F(1,26)= 14.58, p<0.01	DEP
Grooming [sec]	F(1,26)= 1.81, n.s.	F(1,26)= 3.92, n.s.	F(1,26)= 15.02, p<0.01	DEP
Jumping [n]	F(1,26)= 1.58, n.s.	F(1,26)= 0.29, n.s.	F(1,26)= 5.02, p<0.05	TMT
Rampage [sec]	F(1,26)= 1.76, n.s.	F(1,26)= 1.76, n.s.	F(1,26)= 1.76, n.s.	-
Scratching [sec]	F(1,26)= 1.07, n.s.	F(1,26)= 1.16, n.s.	F(1,26)= 15.21, p<0.01	DEP
Activity [sec]	F(1,26)= 0.14, n.s.	F(1,26)= 1.76, n.s.	F(1,26)= 21.25, **p<0.001**	DEP
Faeces [n]	F(1,26)= 2.56, n.s.	F(1,26)= 6.48, p<0.05	F(1,26)= 0.18, n.s.	-
