# Supplementary material for: A Jacob/Nsmf Gene Knockout Results in Hippocampal Dysplasia and Impaired BDNF Signaling in Dendritogenesis
Source: PLoS Genet. 2016 Mar 15;12(3):e1005907. doi: 10.1371/journal.pgen.1005907 (PMC4792503; doi:10.1371/journal.pgen.1005907)
Supplement: S2 Table — Data are presented as mean ± SEM, using unpaired t-tests (Welch’s test); data collected from pairs of mice of each genotype were analyzed. (PDF) [file pgen.1005907.s003.pdf]

S2 Table. Social interaction test with *Jacob/Nsmf* ko and and wt mice. Data are presented as mean ± SEM, using unpaired t-tests (Welch's test); data collected from pairs of mice of each genotype were analyzed.

Parameter	***-/- [n=8]***	***+/+ [n=7]***	***t-test (Welch-test)***
Latency to first social contact [s]	21.25 ± 5.11	22.86 ± 5.29	T=-0.22, df=12.84, n.s.
Number of contacts [n]	172.88 ± 29.33	127.00 ± 26.5	T=1.16, df=12.99, n.s.
Time spent in contact [s]	15.16 ± 2.75	9.33 ± 1.92	T=1.74, df=12.13, n.s.
Mean distance between animals [cm]	30.04 ± 1.89	34.03 ± 1.62	T=-1.60, df=12.92, n.s.
Active social interaction [%]	42.66±3.83	35.83 ± 3.03	T=1.40, df=12.70, n.s.
Passive social interaction [%]	0.07 ± 0.07	0.00 ± 0.00	T=1.00, df=7.00, n.s.
Stay in the center area [%]	11.76 ± 1.72	12.46 ± 1.69	T=-0.29, df=12.96, n.s.
Stay in the edge area [%]	87.89 ± 1.73	87.24 ± 1.72	T=0.27, df=12.93, n.s.
Travelling distance [m]	63.23 ± 7.21	69.52 ± 12.46	T=-0.44, df=9.76, n.s.
Aggressive behaviour [n]	6.13 ± 2.96	0.14 ± 0.14	T=2.02, df=7.03, p<0.05
Sniffing [n]	37.00 ± 2.57	34.86 ± 2.33	T=0.62, df=13.00, n.s.
Anogenital Sniffing [n]	42.25 ± 4.64	31.14 ± 3.29	T=1.95, df=12.21, p<0.05
Following [n]	17.63 ± 4.95	10.43 ± 2.51	T=1.30, df=10.26, n.s.
Defecation [n]	1.63 ± 1.00	3.14 ± 0.77	T=-1.20, df=12.60, n.s.
