# Supplementary figures and images for: A Jacob/Nsmf Gene Knockout Results in Hippocampal Dysplasia and Impaired BDNF Signaling in Dendritogenesis
Source: PLoS Genet. 2016 Mar 15;12(3):e1005907. doi: 10.1371/journal.pgen.1005907 (PMC4792503; doi:10.1371/journal.pgen.1005907)

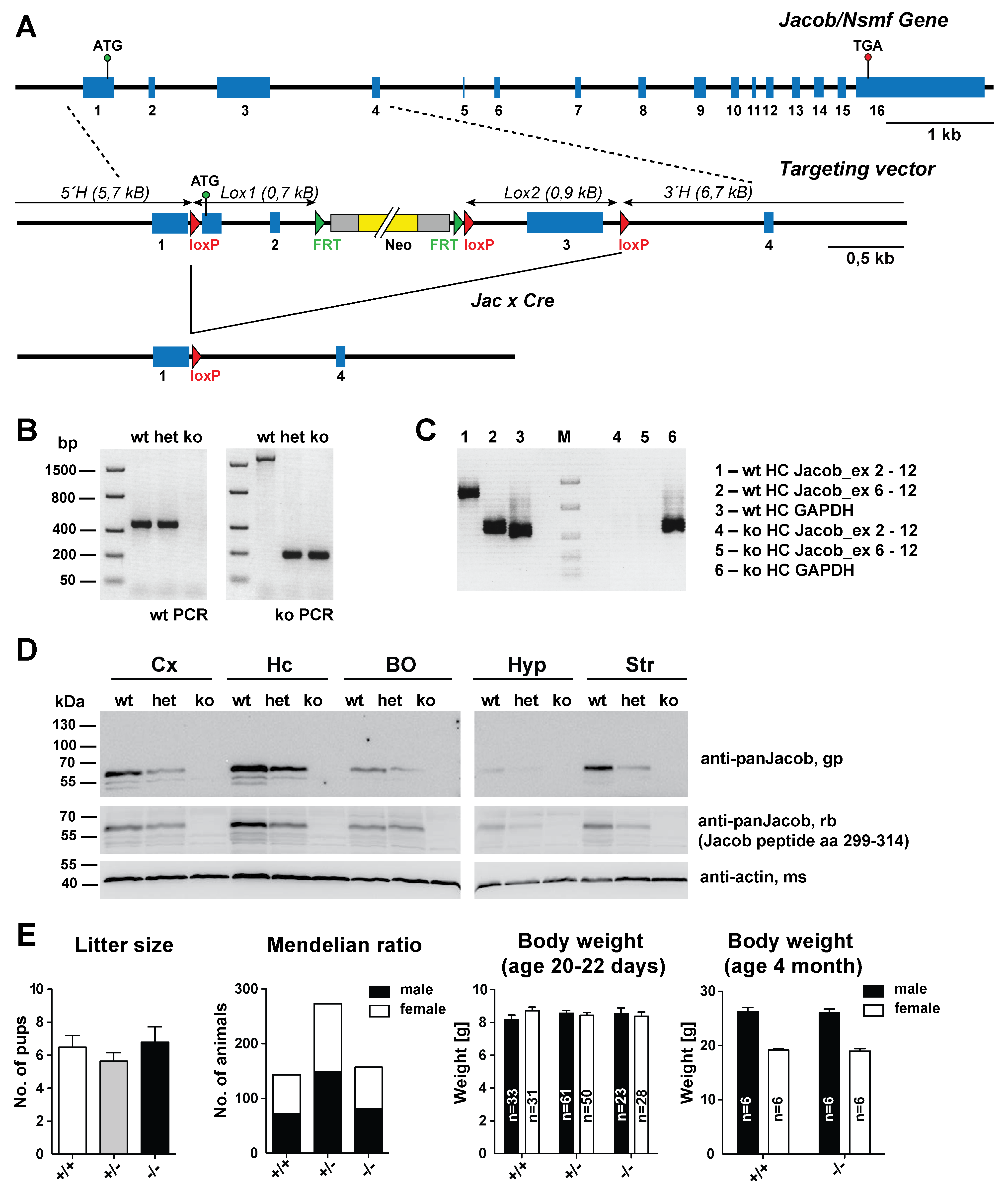

Supplement: S1 Fig — (A) Jacob/Nsmf gene structure and targeting construct. Exons 1–3 were flanked by loxP sites to enable Cre-mediated deletion. (B) Genotyping strategy to differentiate between wild-type (wt), heterozygous (het) and homozygous (ko) mice with specific primers. (C) RT-PCR on wt (lane 1, 2) and Jacob/Nsmf ko mouse mRNA (lane 4, 5) shows that all known splice isoforms are absent in ko mice. (D) Western blots on brain tissue from different areas of wt, Jacob/Nsmf het and ko mice (Cx, Cortex, Hc, hippocampus, Bo, olfactory bulb, Hyp, hypothalamus, Str, striatum). (E) Litter size of homozygous breeders is comparable to heterozygous and wt breeding pairs (n = 4). The mendelian ratio of litters from heterozygous breeders is as expected. Body weights (age of 3 weeks and 4 months) of Jacob-deficient mice do not differ from wt mice. (TIF) [file pgen.1005907.s004.tif]

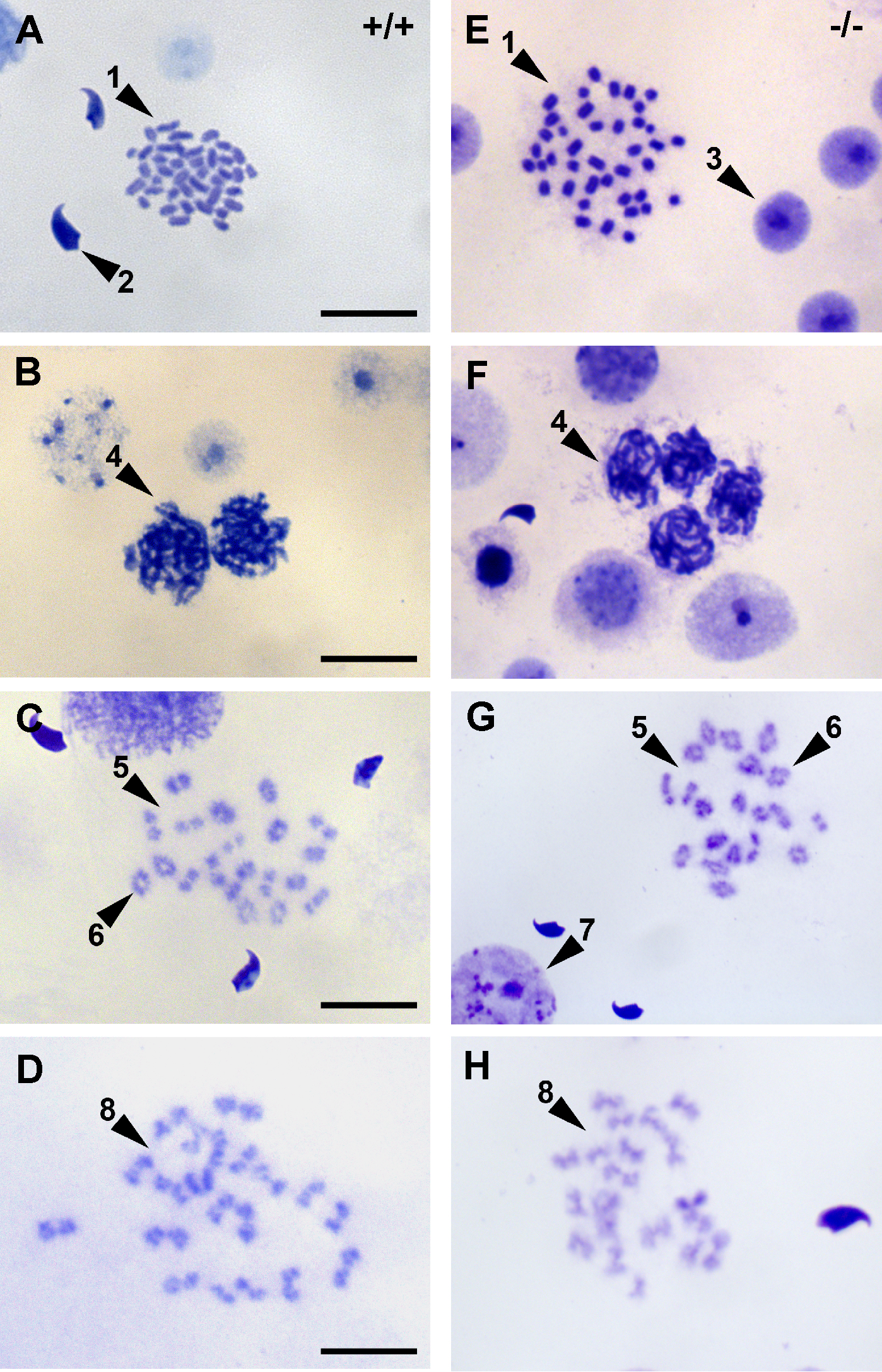

Supplement: S2 Fig — (A, E) The chromosome preparation shows the normal 40 acrocentric chromosomes (arrowhead 1, mitosis chromosomes during cell division, 2, immature sperm cell, 3, spermatogonia). (B, F) Arrowhead 4 shows condensation of chromosomes during Metaphasis I—Pachytene. (C, G) Arrowhead 5 shows separation of the homologous chromosomes after exchanges, 6 show chromosomal crossovers. A tetrad consists of the two homologous chromosomes with their four chromatides. 7 displays a Prophasis I cell. (D, H) Arrowhead 8 indicates Metaphasis II chromosomes. Scale bar, 5 μm. (TIF) [file pgen.1005907.s005.tif]

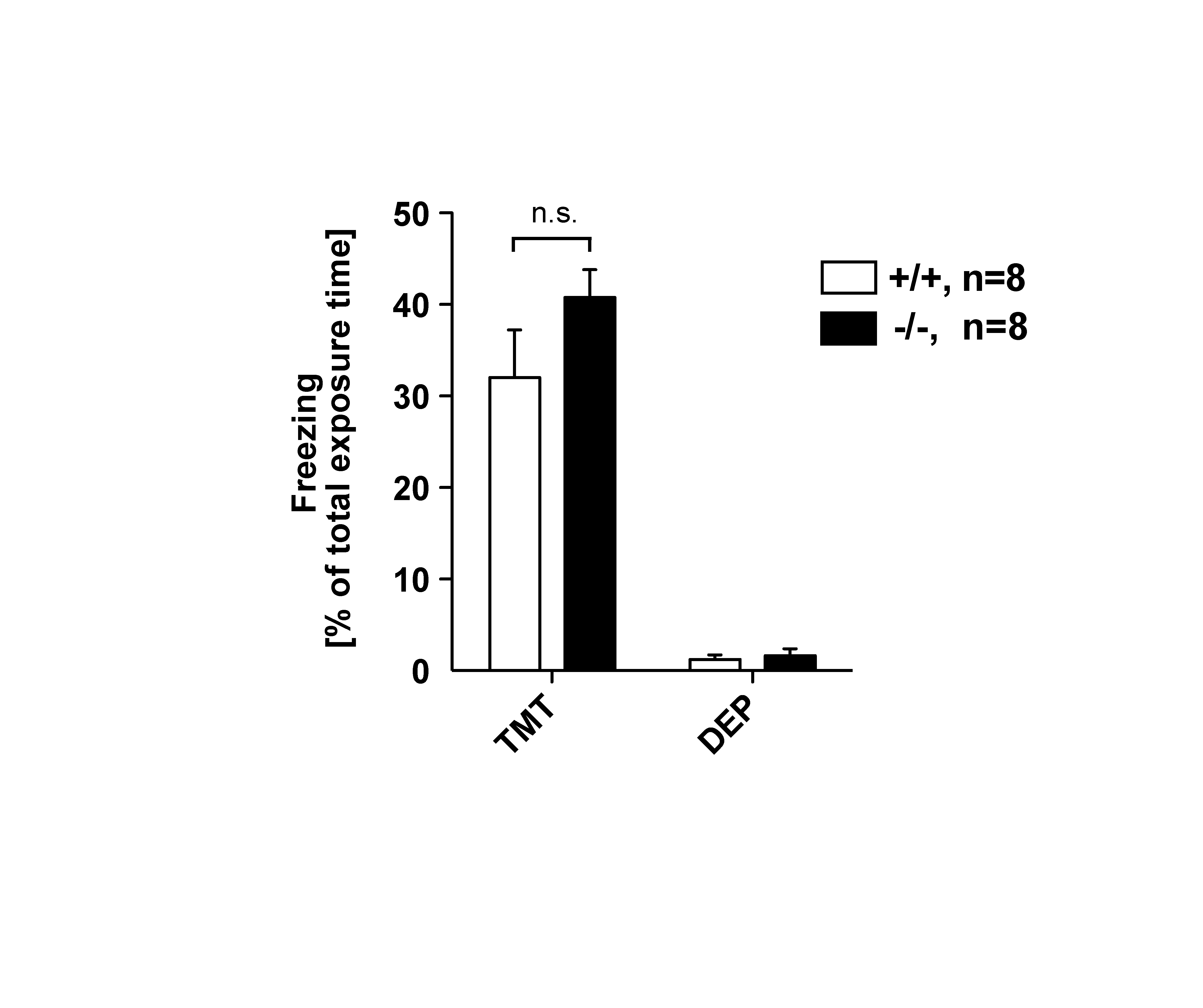

Supplement: S3 Fig — To test anosmia or hyposmia in Jacob/Nsmf ko mice and wt controls (n = 8, males) the animals were singly placed into the center of the box. Freezing behavior was analyzed for 15 min either to TMT or DEP that was pipetted on filter papers and administered through a side lid. Odor exposition experiments were analyzed using multivariate analyses of variance (MANOVA) with ODOR (two levels: TMT and DEP) and GENOTYPE (two levels: +/+ and -/-) as the between-subject factors. Data are presented as mean ± SEM. (TIF) [file pgen.1005907.s006.tif]

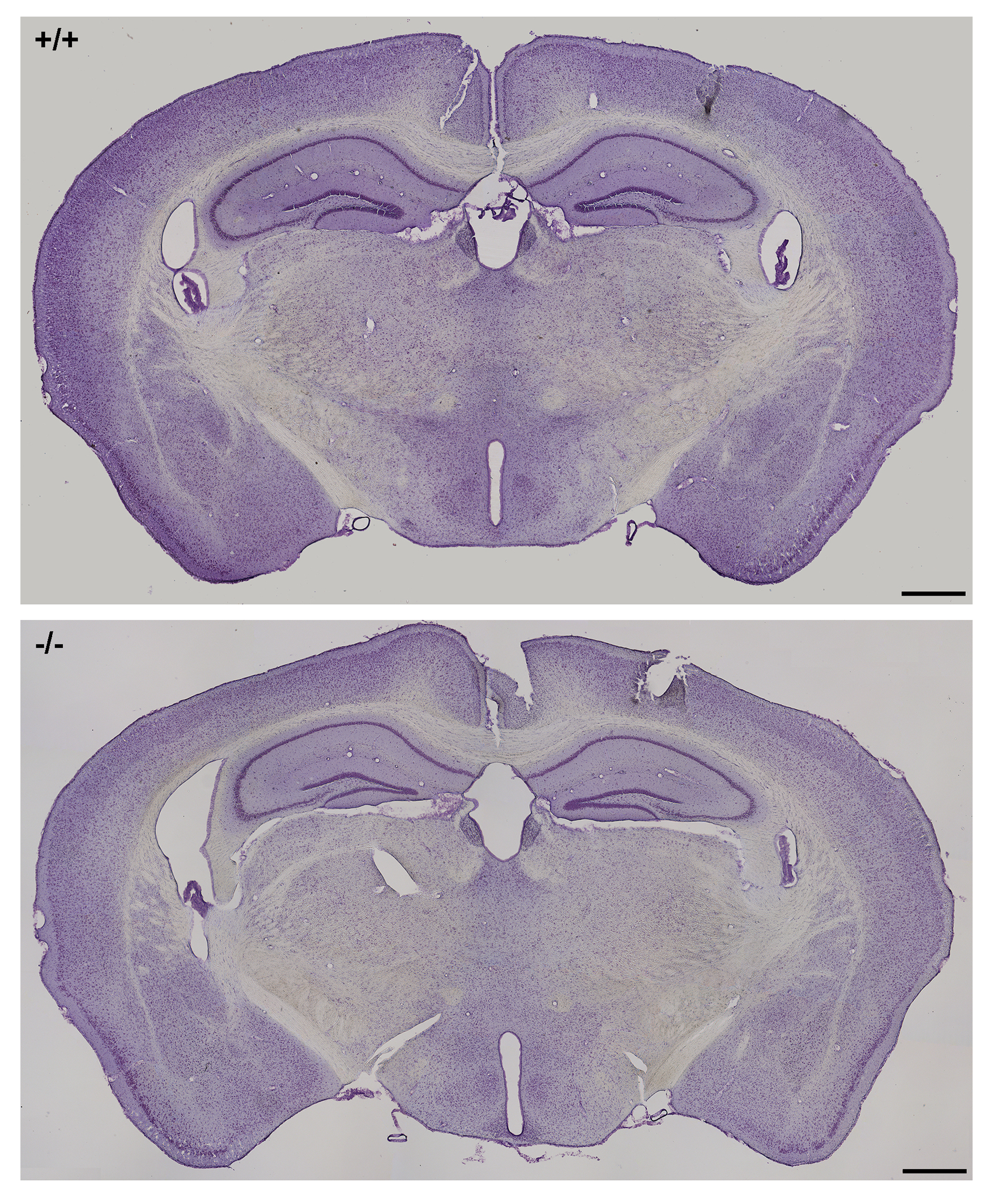

Supplement: S4 Fig — Microphotographs of Nissl-stained coronal sections displaying the brain morphology of wt (+/+) and Jacob/Nsmf ko (-/-) mice at this level (Bregma -1.94 mm). There are no differences in general morphology between both genotypes (scale bar 1 mm). (TIF) [file pgen.1005907.s007.tif]

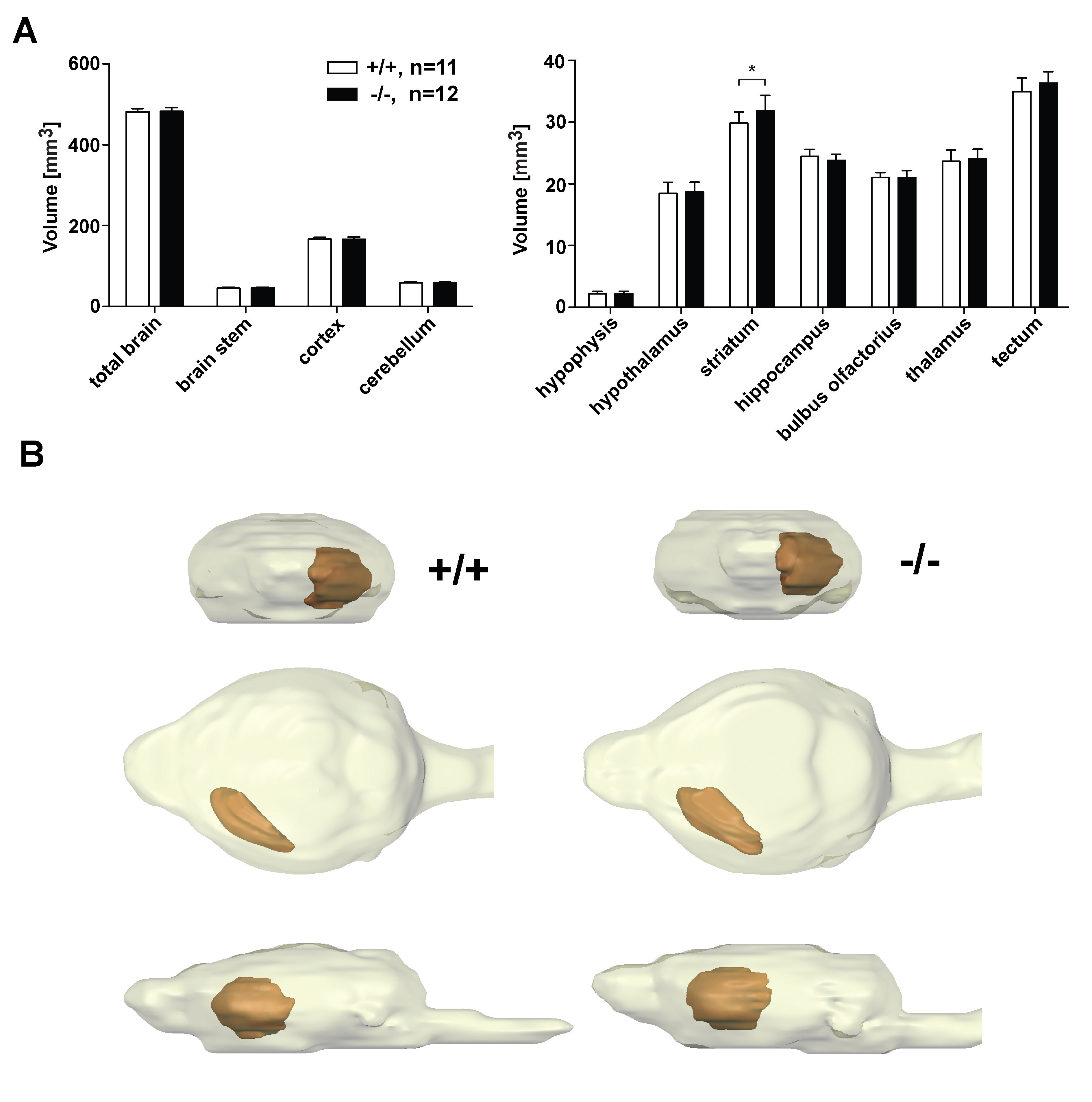

Supplement: S5 Fig — (A) Comparison of the total brain volume and the volume of different brain structures of Jacob/Nsmf ko (-/-)and wt (+/+) littermates. Significant difference between the two groups (p<0.05) is indicated by an asterisk. (B) 3D surface rendering of the striatum in wt (+/+) and ko (-/-) mice. (TIF) [file pgen.1005907.s008.tif]

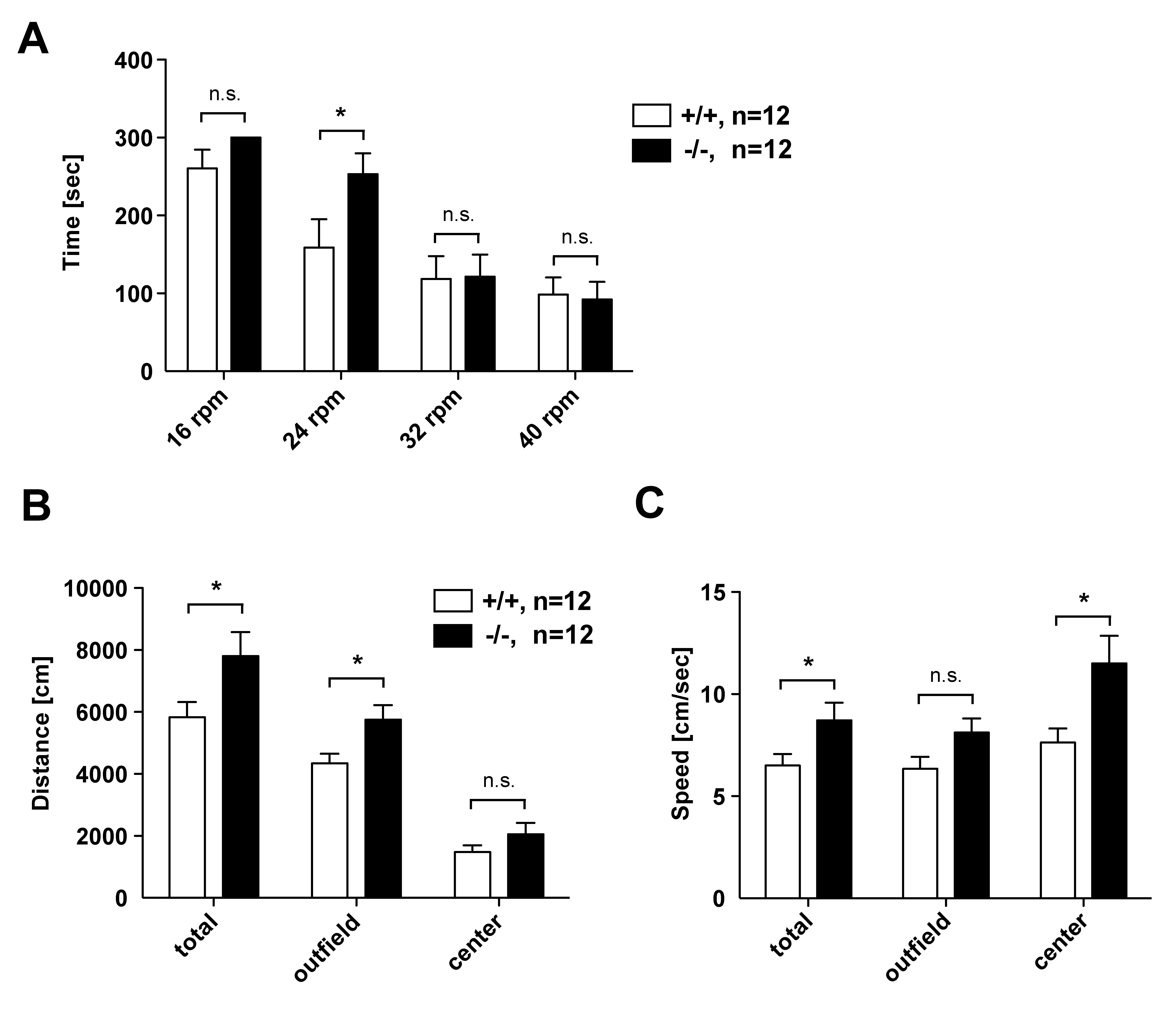

Supplement: S6 Fig — (A) Motor behavior of Jacob-deficient mice and wt littermates (n = 12, each genotype, both sexes) was analyzed on the rotarod. (B, C) Spontaneous behavior was tested in the open field and revealed significant differences between wt and Jacob/Nsmf ko mice towards speed and distance covered (n = 12, each genotype, both sexes; two-way ANOVA, *p ≤ 0.05). (TIF) [file pgen.1005907.s009.tif]

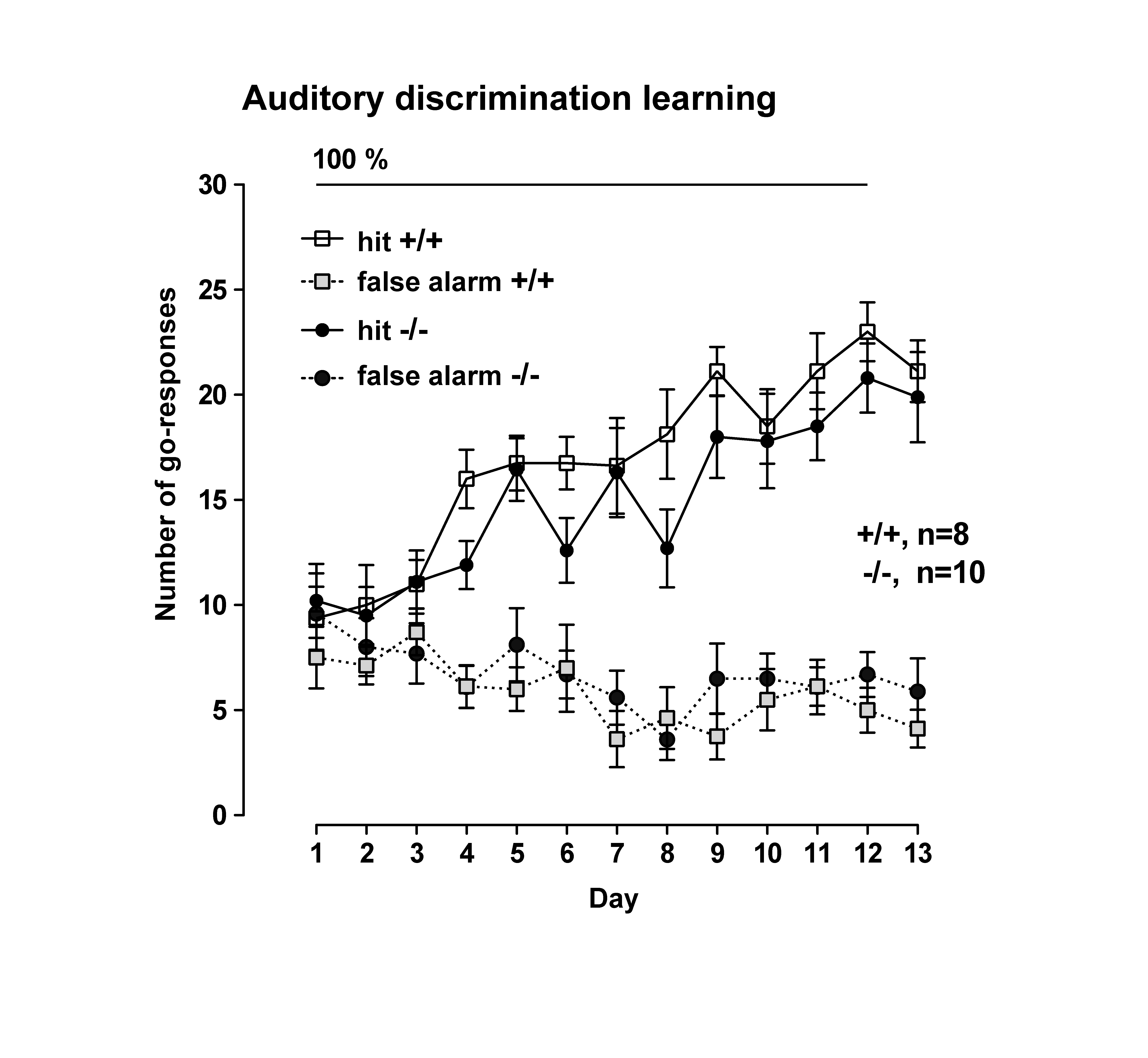

Supplement: S7 Fig — Male mice (Jacob/Nsmf ko: n = 10, wt littermates: n = 8) were trained in a two-way shuttle box GO/NO-GO task to discriminate between sequences of rising (4–8 kHz, CS+) and falling (8–4 kHz, CS-) frequency modulated tones. Mice had to respond the presentation of CS+ by a hurdle crossing (hit), while they had to remain in the current compartment during 6s of CS- presentation (correct rejection). Errors (misses, false alarms) were punished by a mild foot-shock. Each of the 13 daily training sessions consisted of 60 trials with 30 randomized presentations of CS+ and CS-, respectively. (Two-way ANOVA, values are mean ± SEM). (TIF) [file pgen.1005907.s010.tif]

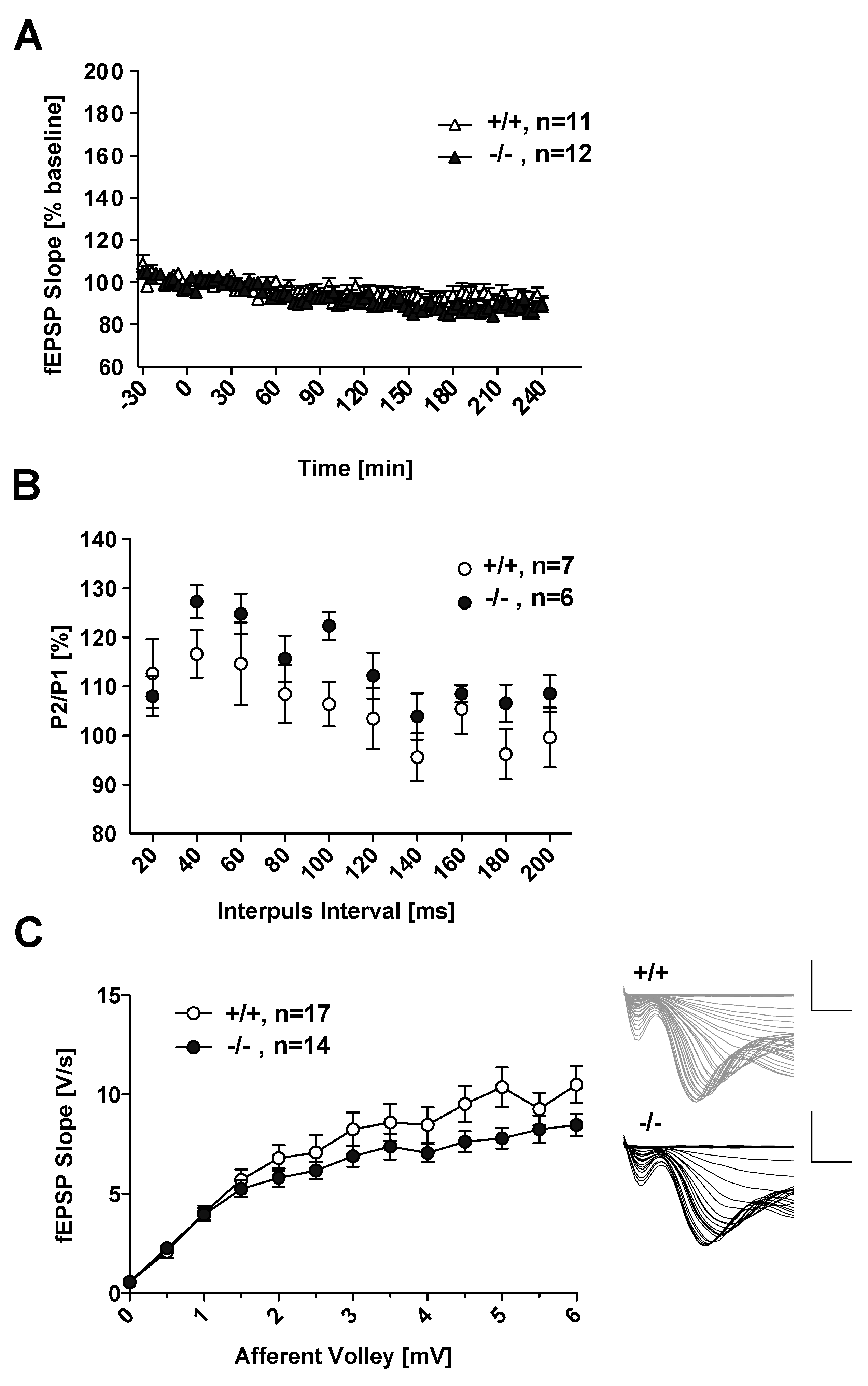

Supplement: S8 Fig — (A) Baseline stability experiments revealed no difference between wt and Jacob/Nsmf ko mice. (B) Paired-pulse facilitation ratio (PPF ratio, P2/P1) was plotted against different inter-pulse intervals as second divided by first fEPSP. (C) Input-output curves showing the relationship between the fEPSP slope and the afferent volley in wild-type (wt) or Jacob/Nsmf knockout mice (ko). There was a trend to smaller fEPSP slopes in the knockout animals, but there were no statistically significant differences between the input-output curves. Field potential traces for both groups are shown as inset (grey traces: wt, black traces: ko). Scale bars 5mV/1ms. Data are represented as mean ± SEM. (TIF) [file pgen.1005907.s011.tif]

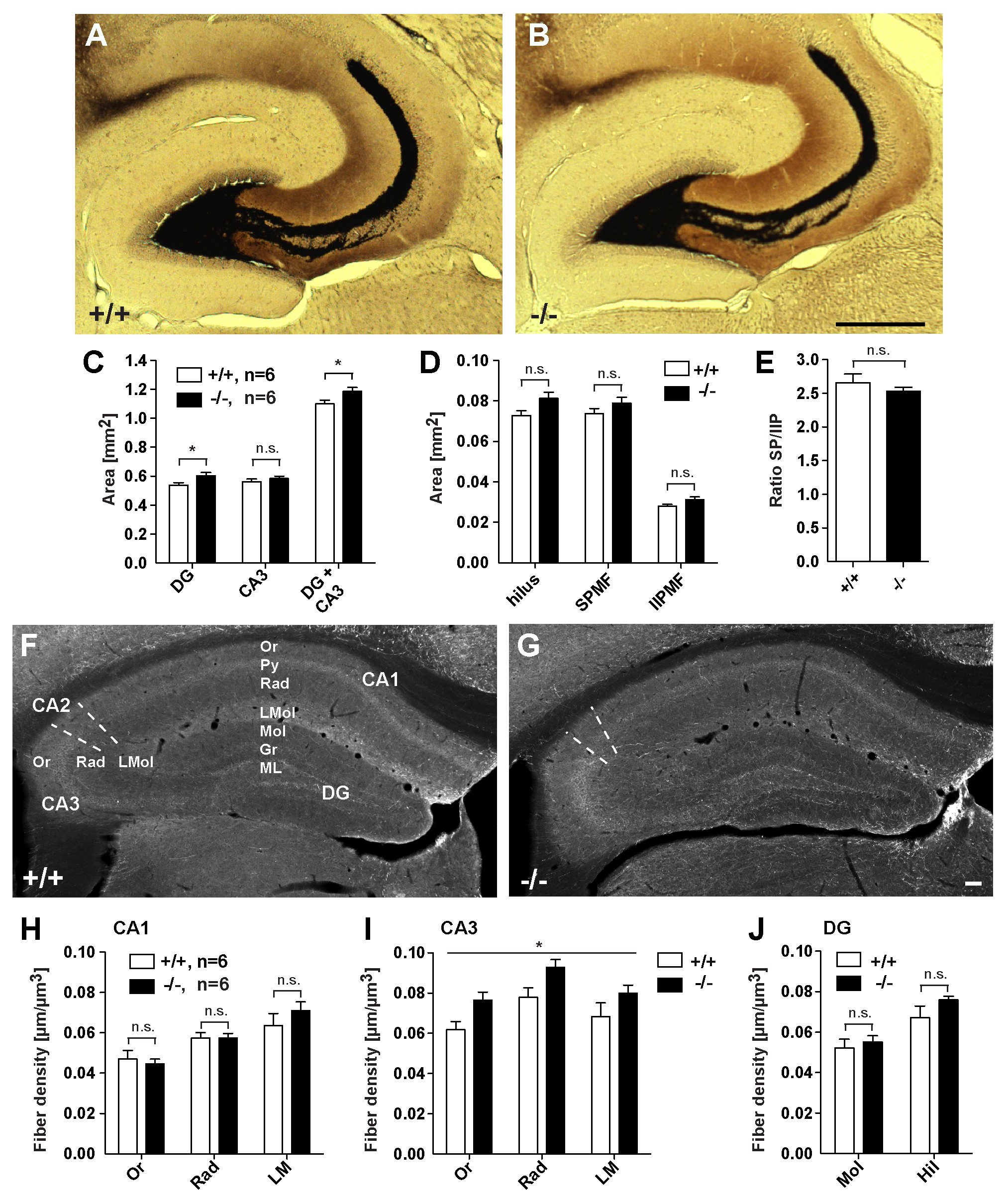

Supplement: S9 Fig — Representative TIMM-stained hippocampal area in wt (A) and Jacob/Nsmf ko mice (B). In Jacob/Nsmf ko mice the complete DG is significant enlarged compared to wt mice. (C-E) Morphometry in the hippocampus. (C) Significant differences could be detected in complete DG and DG + CA3 area. The TIMM-positive structures in hippocampus (D) and also the ratio of suprapyramidal mossy fibers (SPMF) and infra- and intrapyramidal mossy fibers (IIPMF) (E) did not show any differences. (ANOVA * p<0.05; scale bar in B = 500 μm). (F, G) Microphotographs of coronal sections showing the distribution of tyrosine hydroxylase (TH)-IR fibers in dorsal hippocampus of wt and Jacob/Nsmf ko mice. (H-J) A significant difference between genotypes was found in CA3 (I), but not in CA1 (H) and DG (J). Two way repeated measures ANOVAs were performed using LAYER (three levels in CA1 and CA3: Or, stratum oriens, Rad, stratum radiatum, LMol, stratum lacunosum moleculare; two levels in DG: Mol, stratum moleculare, ML, stratum multiforme) as within-subject factor and MOUSE LINE (two levels: wt and Jacob/Nsmf ko mice) as between-subject factor. Scale bar in G = 100 μm. (TIF) [file pgen.1005907.s012.tif]

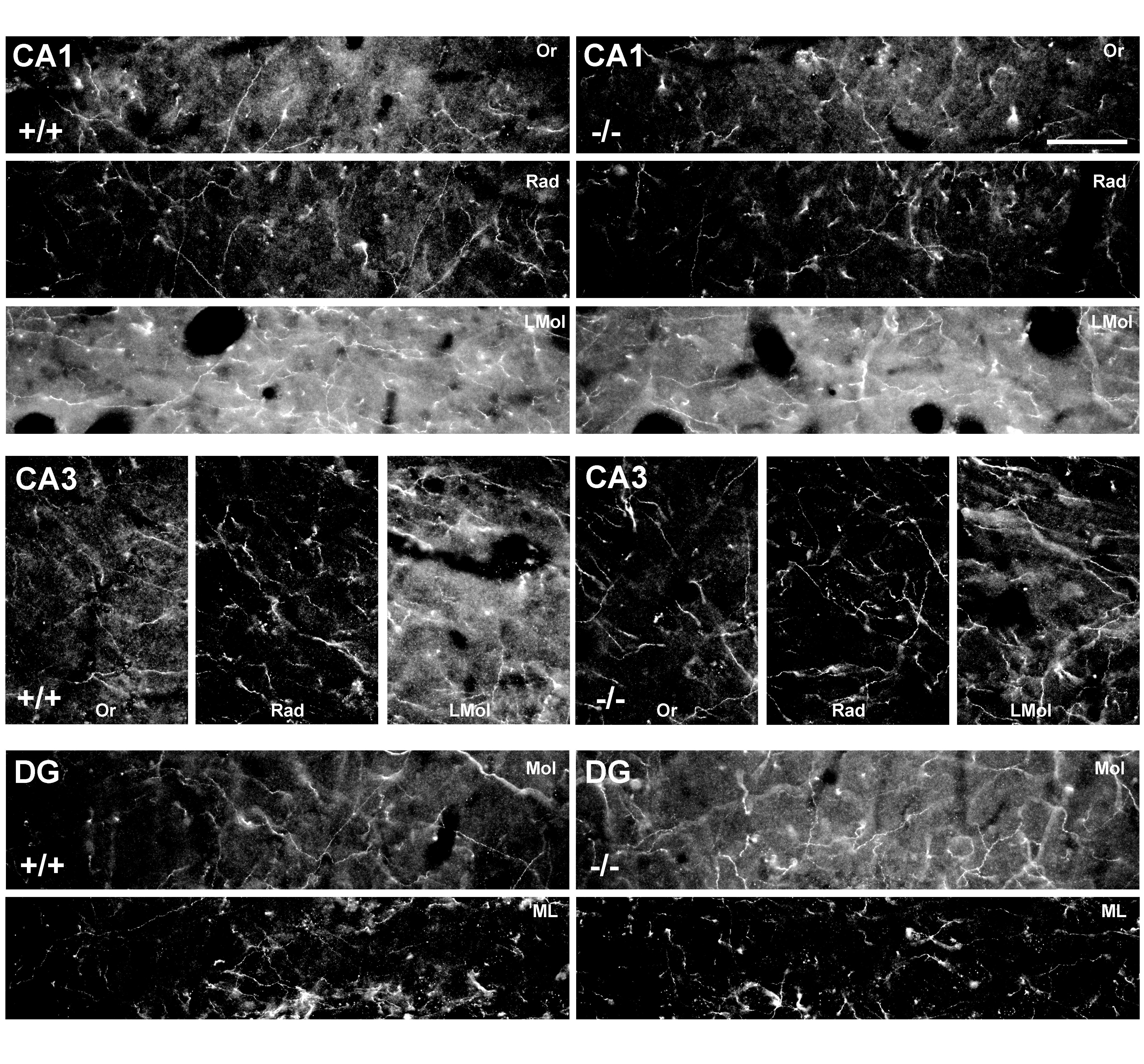

Supplement: S10 Fig — For overview and quantification see S8F–S8J Fig. From top down images from CA1, DG and CA3 layers (CA1 and CA3: Or, Rad, LMol; DG: Mol, ML) of each genotype are shown (scale bar 50 μm). The exemplary images are taken from the same mouse, respectively. (TIF) [file pgen.1005907.s013.tif]

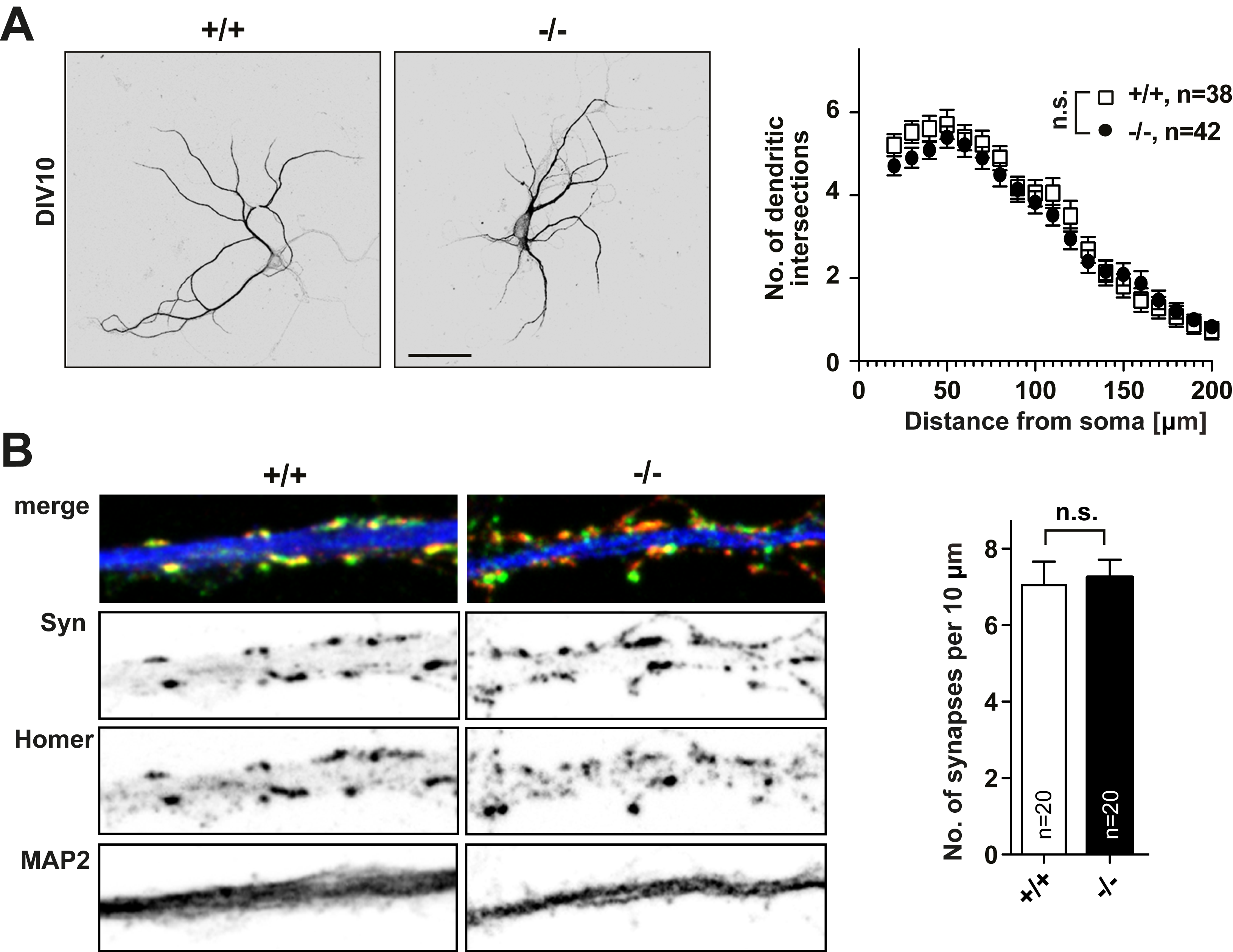

Supplement: S12 Fig — (A) Representative micrographs of wt and Jacob/Nsmf ko cortical neurons immuno-stained with MAP2 at DIV10. For sholl analysis the number of dendritic intersections of wt and Jacob/Nsmf ko cortical neurons was plotted against the distance. (B) Jacob/Nsmf ko neurons do not display a reduced number of synaptic contacts as compared to wt controls. Representative micrographs of DIV15 wt and Jacob/Nsmf ko cortical neurons primary distal dendrites immuno-stained with MAP2, Homer1 and Synaptophysin. Co-localization of synaptic puncta per 10 μm was quantified. A Student´s t-test did not show significant differences. Scale bar in (A) = 50 μm, panels in (B) = 20 μm. (TIF) [file pgen.1005907.s015.tif]

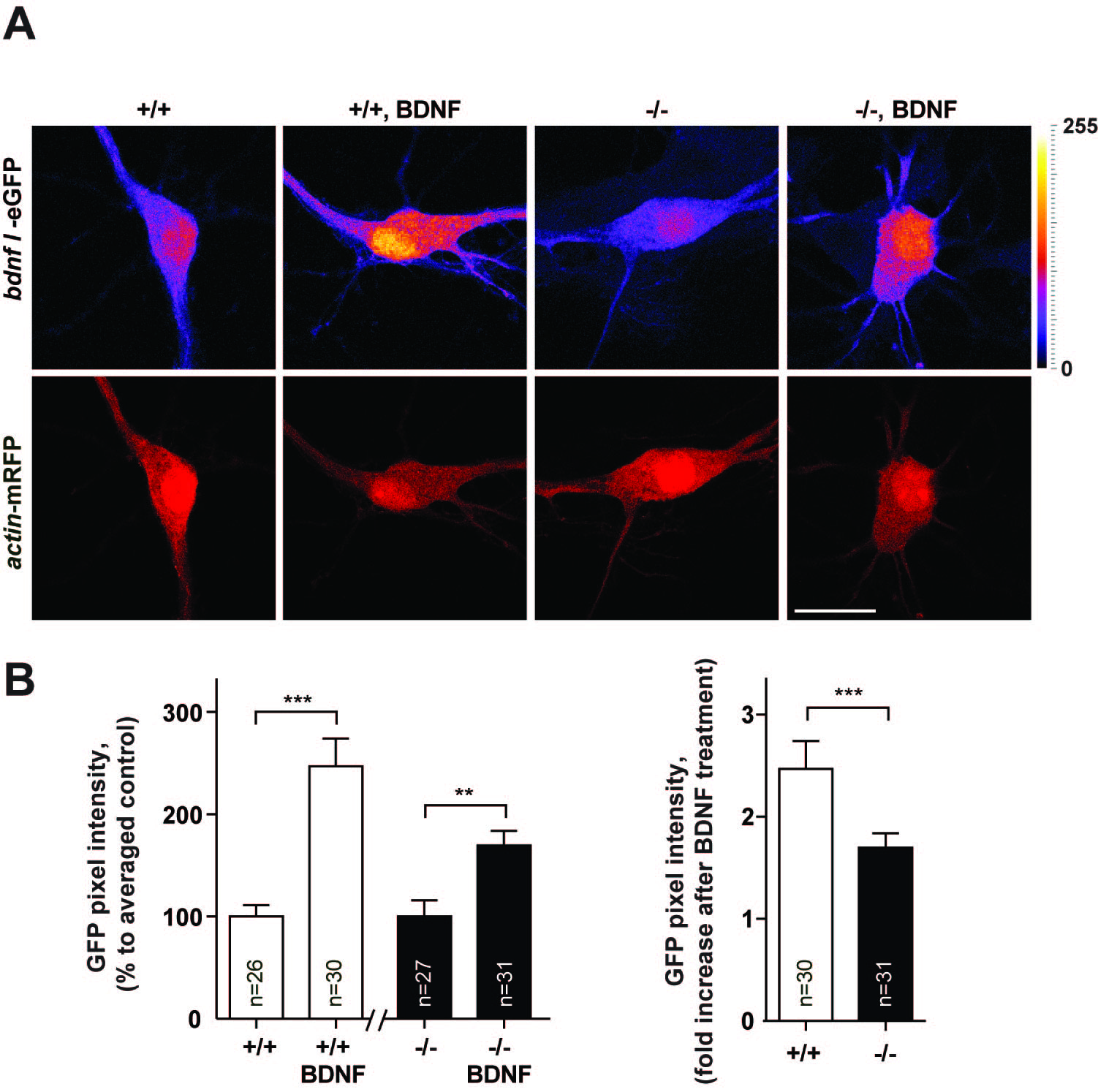

Supplement: S13 Fig — (A) DIV10 hippocampal neurons were transfected with construct overexpressing GFP under Bdnf I+II promoter. After 24h of expression and treatment with BDNF, cultures were fixed and confocal images were acquired. (B) In Jacob/Nsmf ko neurons as well as wt there was significant increase after of GFP intensity after BDNF treatment, however in case of ko neurons the increase in GFP intensity was lower. Two-way ANOVA with Bonferroni posttest, ***p<0.001; **p<0.01 (B, left side) and two-tailed unpaired t-test (B, right side) ***p<0.001. Scale bar is 20 μm. (TIF) [file pgen.1005907.s016.tif]

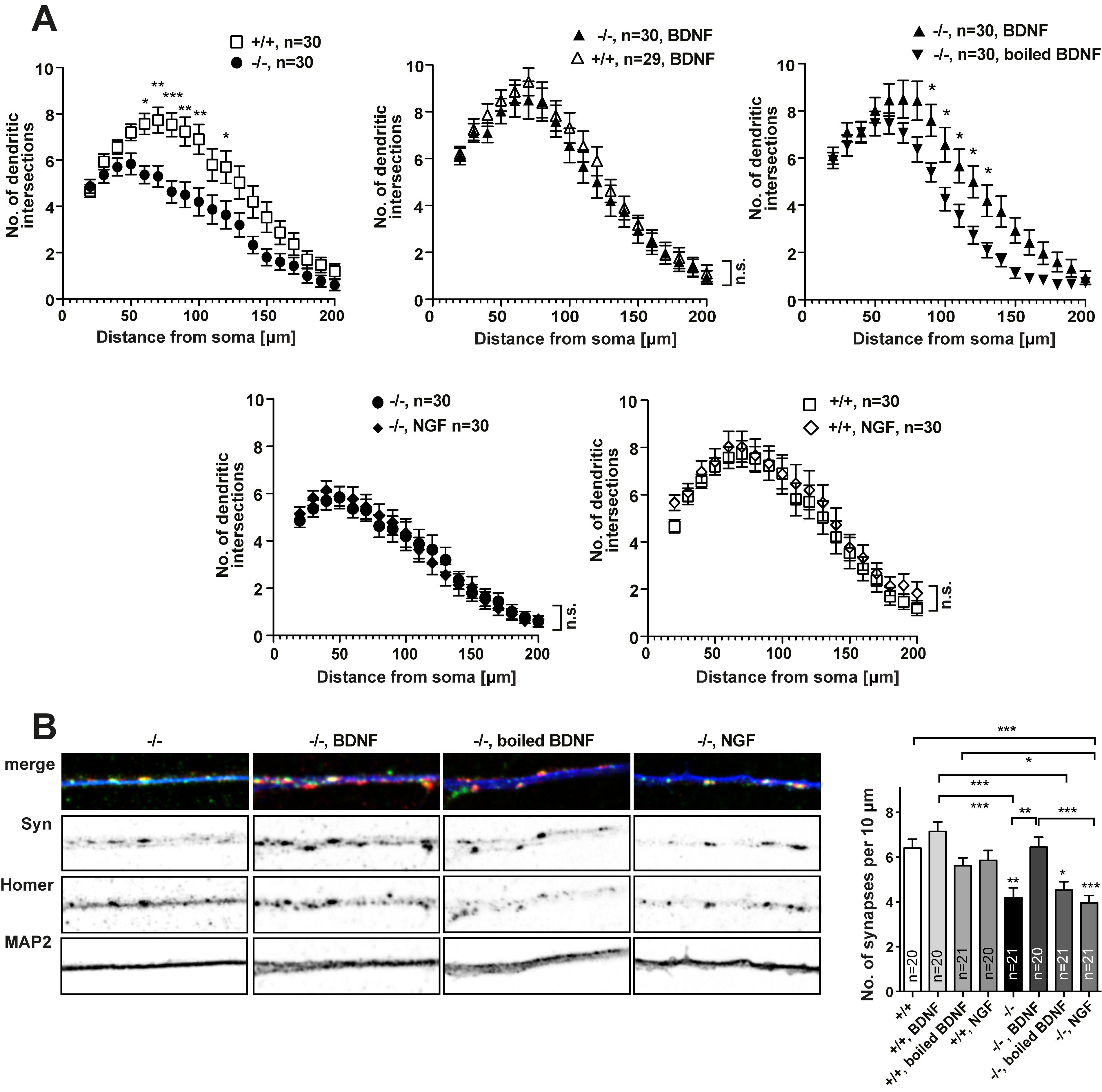

Supplement: S14 Fig — Jacob/Nsmf ko mouse hippocampal neurons and wt neurons were treated with BDNF, boiled BDNF or NGF (all 100 ng/ml) at DIV2 and DIV6, fixed at DIV10 and stained for MAP2. (A) Graphs representing the number of dendritic intersections of wt and jacob/nsmf ko hippocampal neurons plotted against the distance. At DIV10 Jacob/Nsmf ko hippocampal neurons display a simplified dendritic tree as compared to wt neurons. (B) Jacob/Nsmf ko neurons display a reduced number of synaptic contacts as compared to wt controls. Representative micrographs of DIV15 wt and Jacob/Nsmf ko hippocampal neurons primary distal dendrites immuno-stained with MAP2, Homer1 and Synaptophysin, untreated, treated with BDNF, with boiled BDNF or NGF. Co-localizing synaptic puncta were quantified per 10 μm. Statistical differences were analysed with two-way ANOVA and Post-hoc Bonferroni tests (* p<0.05, **p<0.01, ***p<0.001). Panels in B = 20 μm. (TIF) [file pgen.1005907.s017.tif]

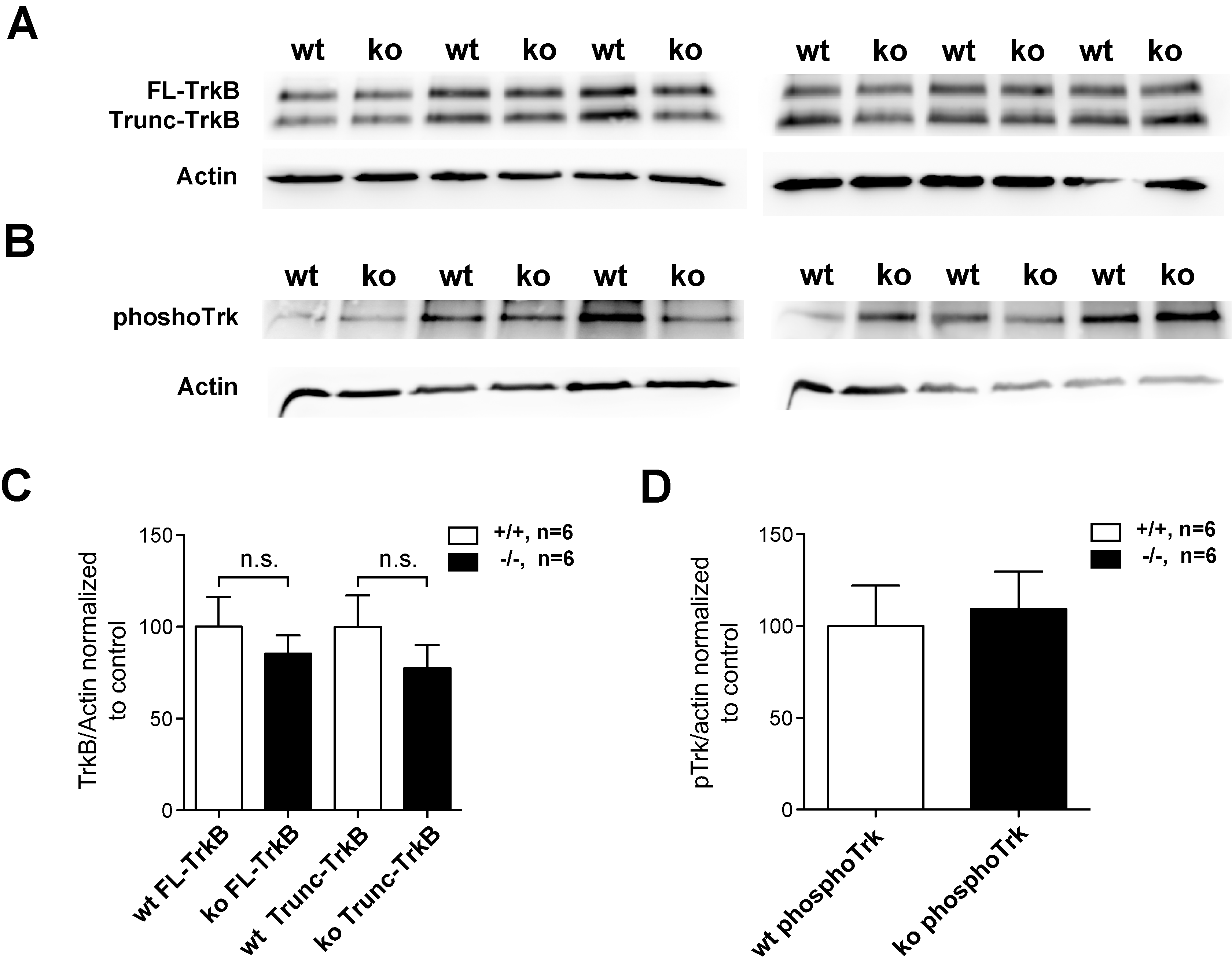

Supplement: S15 Fig — Immunoblots of hippocampal lysates from 6 wt and 6 Jacob/Nsmf ko animals: (A) Anti Trk-B antibody detects both full-length (FL) and truncated (Trunc) forms of TrkB. (B) Anti phosphoTrk antibody. The quantification of levels of TrkB (C), pTrk (D) normalized to actin did not reveal statistically significant changes. (TIF) [file pgen.1005907.s018.tif]

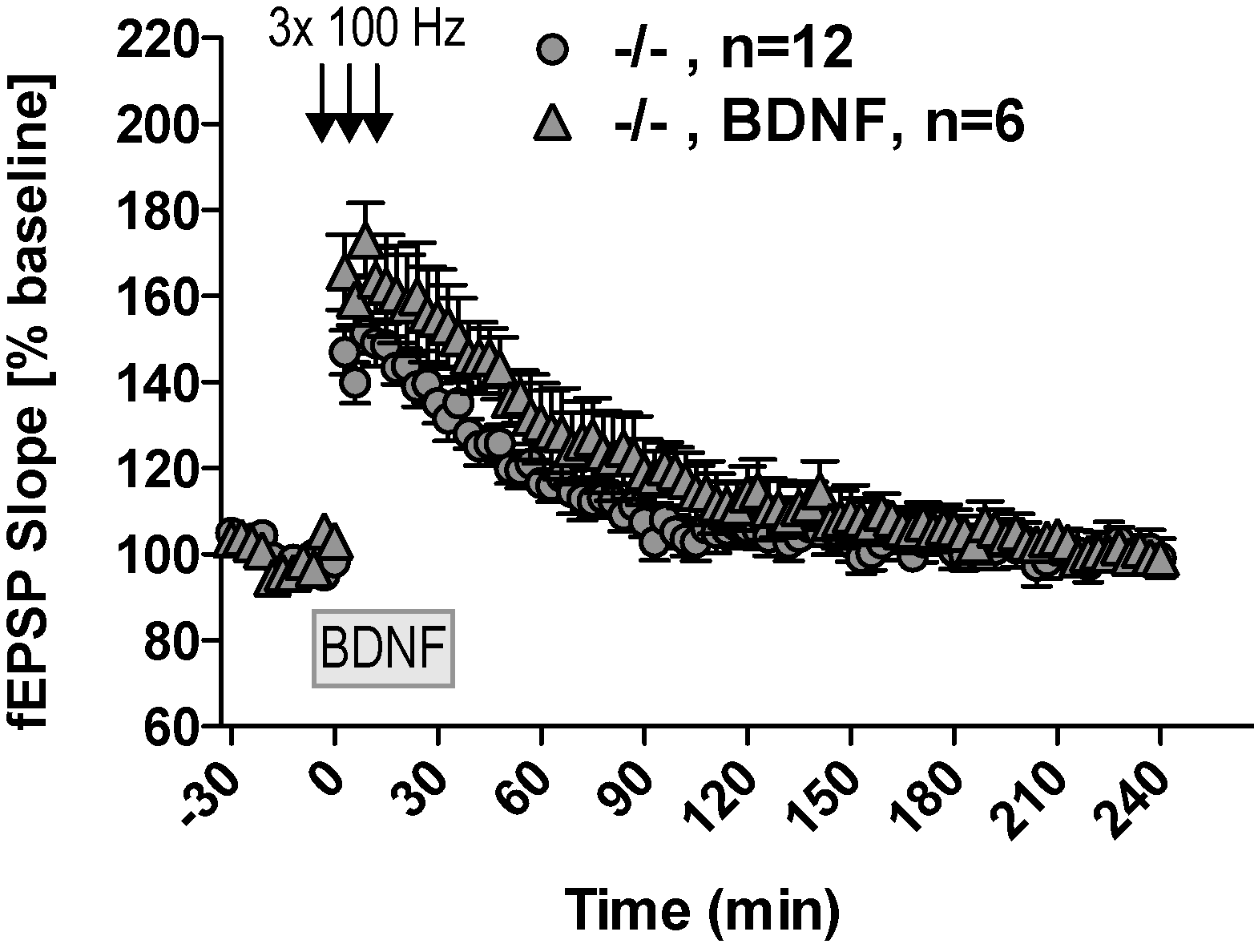

Supplement: S16 Fig — Application of 100ng/ml BDNF during tetanus has no effect on LTP in ko mice. The arrows indicate high frequency stimulus (HFS) lasting 1s at 100Hz, 3x HFS. The horizontal bar indicates the period during BDNF was added into the bath solution. Data are represented as mean ± SEM. (TIF) [file pgen.1005907.s019.tif]

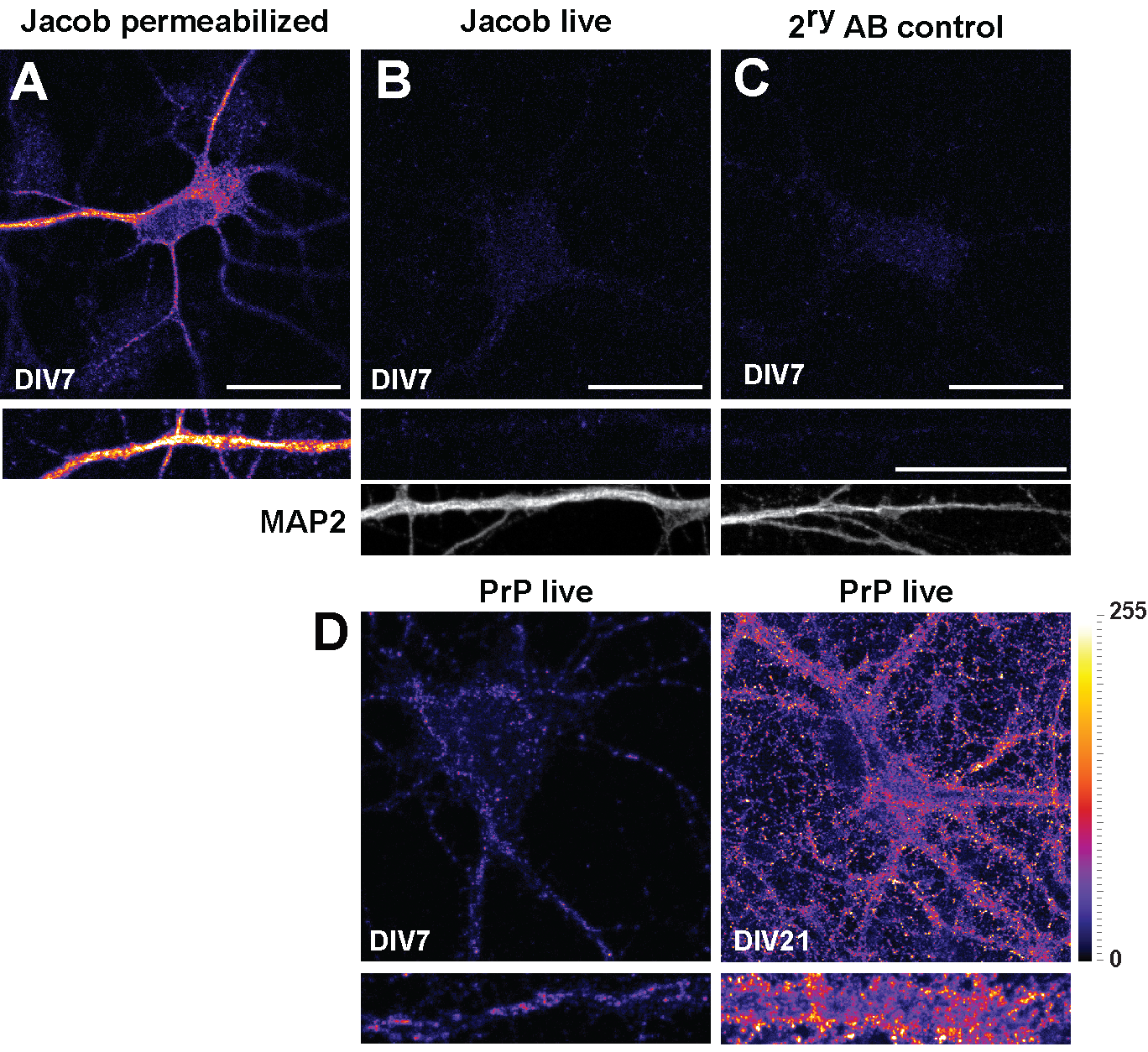

Supplement: S17 Fig — (A) Primary neurons (DIV7) express Jacob intracellularly (permeabilized), but not extracellularly (B) (Jacob live) as there is no higher staining than in secondary antibody controls (C). MAP2 staining was included to outline dendrites. (D) Live-staining of Prion protein (DIV7 and DIV21) proves reliability of the live-staining protocol. Scale bar is 20μm. (TIF) [file pgen.1005907.s020.tif]

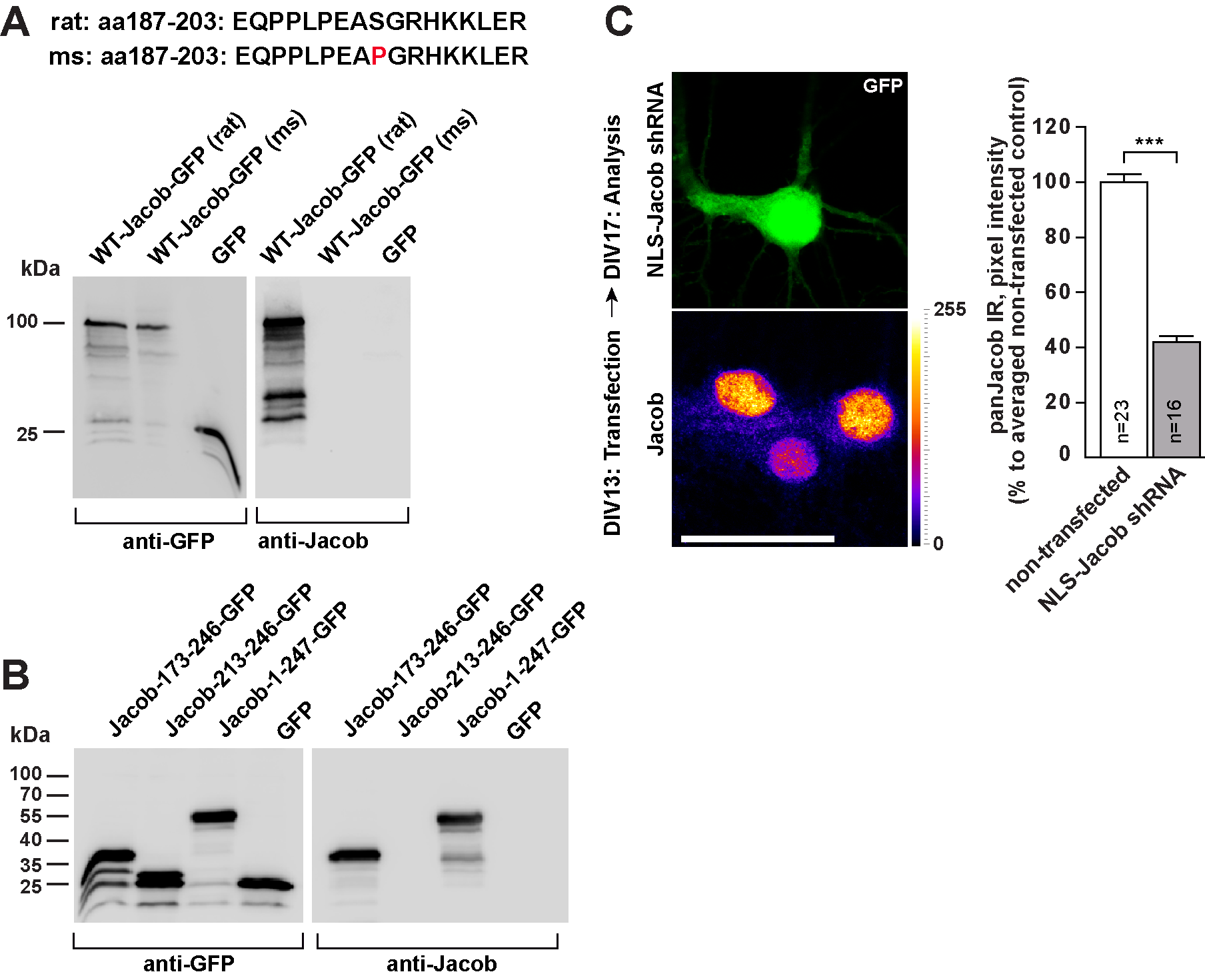

Supplement: S18 Fig — (A) pan-Jacob antibodies were generated against rat N-terminal peptide aa 187–203 corresponding to rat Jacob sequence which is slightly different from the mouse Jacob sequence. Antibodies do recognize heterologously expressed rat WT-Jacob-GFP, but not the mouse WT-Jacob-GFP protein. (B) Antibodies recognize only the part of Jacob where the amino acid sequence used for rabbit immunization is represented. (C) Plasmid based shRNA knockdown dramatically reduced endogenous Jacob levels in mature hippocampal neurons. Scale bar is 40μm. (TIF) [file pgen.1005907.s021.tif]

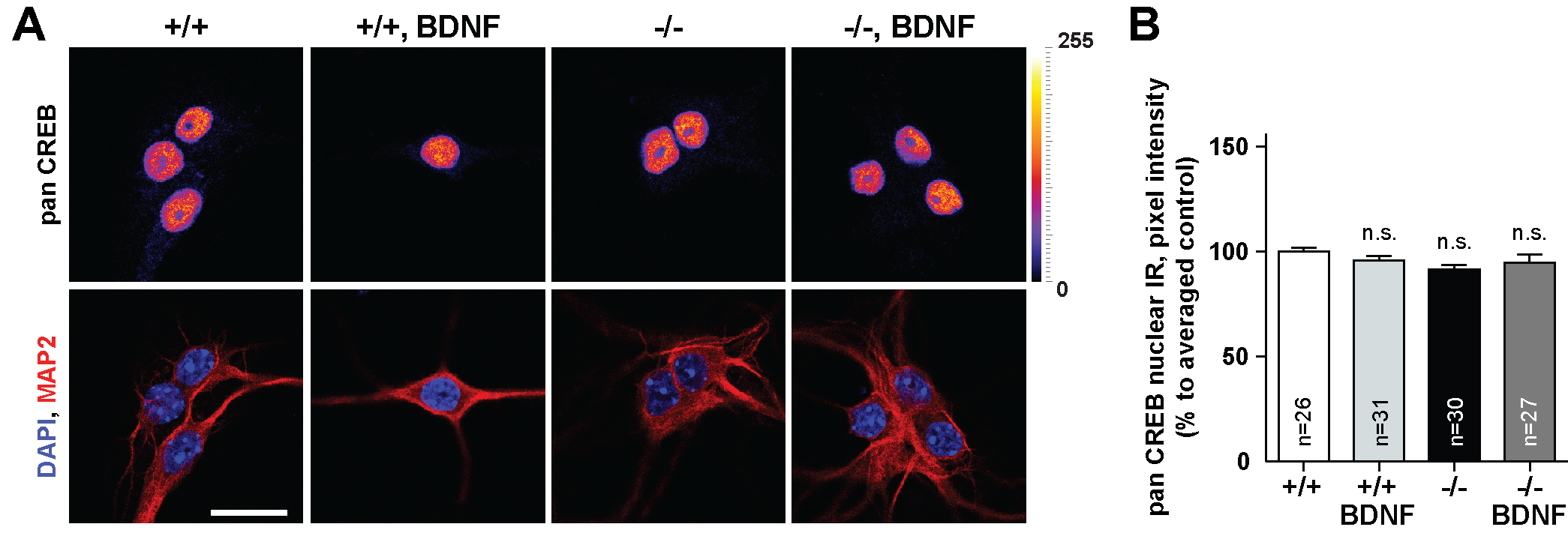

Supplement: S19 Fig — (A, B) Following bath application of BDNF (100ng/ml) at DIV10 the total nuclear CREB levels are unchanged in Jacob/Nsmf ko and wt hippocampal neurons. Scale bar is 20μm. (TIF) [file pgen.1005907.s022.tif]
